# Telenursing contributions in Primary Health Care in the COVID-19 pandemic context: an integrative review

**DOI:** 10.1590/0034-7167-2024-0093

**Published:** 2024-11-22

**Authors:** Patrícia Amidianski, Evangelia Kotzias Atherino dos Santos, Alacoque Lorenzini Erdmann, Carmem Regina Delziovo, Maria Solange Ferreira Alves, Marli Terezinha Stein Backes

**Affiliations:** I Universidade Federal de Santa Catarina. Florianópolis, Santa Catarina, Brasil; II Secretaria de Estado da Saúde de Santa Catarina. Florianópolis, Santa Catarina, Brasil

**Keywords:** Telenursing, Remote Consultation, Nursing, Primary Health Care, COVID-19, Telenfermería, Consulta Remota, Enfermería, Atención Primaria de Salud, COVID-19

## Abstract

**Objective:**

to identify telenursing contributions in Primary Health Care during the COVID-19 pandemic.

**Methods:**

an integrative literature review, conducted between January and August 2022 in the PubMed, CINAHL, LILACS, BDENF, Scopus, WoS, EMBASE and SciELO databases. A total of 493 studies was found, 62 were read in full, and of these, 16 were selected. For analysis, a dynamic reading of the studies and synthesis of the main results were carried out.

**Results:**

the main results highlighted telenursing practice as a challenge for professionals and the population. Among the contributions and positive points and aspects that require improvement, practice showed promise when considered in a post-pandemic scenario.

**Final considerations:**

through telenursing, the population’s access to Primary Health Care was guaranteed in the face of the COVID-19 pandemic. However, a critical look at current technological advances in healthcare is necessary.

## RESUMEN

**Objetivo:**

identificar los aportes de la teleenfermería en la Atención Primaria de Salud en el contexto de la pandemia COVID-19.

**Métodos:**

revisión integrativa de la literatura, realizada entre enero y agosto de 2022 en las bases de datos PubMed, CINAHL, LILACS, BDENF, Scopus, WoS, EMBASE y SciELO. Se encontraron un total de 493 estudios, 62 fueron leídos en su totalidad y de estos, 16 fueron seleccionados. Para el análisis se realizó una lectura dinámica de los estudios y síntesis de los principales resultados.

**Resultados:**

los principales resultados destacaron la práctica de la teleenfermería como un desafío para los profesionales y la población. Entre los aportes y puntos positivos y aspectos que requieren mejora, la práctica se mostró prometedora cuando se la considera en un escenario pospandemia.

**Consideraciones finales:**

a través de la teleenfermería se garantizó el acceso de la población a la Atención Primaria de Salud ante la pandemia de COVID-19. Sin embargo, es necesaria una mirada crítica a los avances tecnológicos actuales en la atención sanitaria.

## INTRODUCTION

Telehealth practice has been encouraged by the World Health Organization (WHO) since 2005 as a strategic means of improving health systems, including the Brazilian Health System (SUS - *Sistema Único de Saúde*)^(1)^. Globally, it is important to highlight the current digital transformation in healthcare, since the “Global strategy on digital health 2020-2025”, proposed by the WHO, appears to strengthen health systems through the use of digital technologies, thus strengthening the concept of universal health^(2)^. In Brazil, the Ministry of Health developed the “Digital Health Strategy for Brazil 2020-2028”, which seeks to update the Brazilian National Health Information and Informatics Policy (PNIIS - *Política Nacional de Informação e Informática em Saúde*), in addition to reaffirming policies, guidelines, ordinances, actions and initiatives within the scope of digital health in SUS^(3)^.

Starting from technological evolution in relation to health, it is necessary to highlight the advent of the COVID-19 pandemic, which considerably accelerated digital health evolution. Declared in March 2020 by the WHO, the COVID-19 pandemic was accompanied by numerous restrictions as a way to combat the spread of the SARS-CoV-2 virus among the population, implementing measures such as social distancing and isolation. With the rapid advancement of the pandemic, health systems collapsed and emergency strategies were needed to ensure the population’s access to healthcare services^(4)^.

From this point on, telenursing was widely encouraged and practiced on a global scale as a way of dealing with the high demand for healthcare services. It is a care modality in electronic/digital format that makes it possible to carry out nursing consultations, monitoring, consultations, inter-consultations, health education and reception actions, mediated by Information and Communication Technologies (ICTs)^(5)^.

In Brazil, among the different areas of health, nursing gained ethical and legal support through Resolution COFEN (Federal Nursing Council - *Conselho Federal de Enfermagem*) 634/2020 to carry out nursing teleconsultation, which was authorized and standardized as a strategy to combat the COVID-19 pandemic, including the use of ICTs in professional skills^(6)^. Due to the persistence of the COVID-19 pandemic, in 2022, COFEN standardized telenursing through COFEN Resolution 696/2022, amended by COFEN Resolution 707/2022^(7)^.

In this situation, it is important to highlight the role of nursing in PHC, considered the gateway to SUS. Nursing gained prominence, mainly, with the emergence of the pandemic and the need to reinvent the environment and workflows digitally. Health guidelines, qualified reception and digital consultations have become valuable practices for combating the pandemic, in addition to being an important strategy for continuity of healthcare^(8)^.

## OBJECTIVE

To identify telenursing contributions in PHC during the COVID-19 pandemic.

## METHODS

### Ethical aspects

This study was carried out on public domain bases, which does not require submission to a Research Ethics Committee.

### Methodological framework

The Mendes, Silveira and Galvão^(9)^ methodological framework was used, who consider the literature review to be a methodology capable of providing scientific support for decision-making and clinical practices in health.

### Study design

This is an integrative literature review that sought to identify, analyze and synthesize results of scientific evidence, in order to generate knowledge regarding the topic and enable new contributions to improving clinical practice^(9)^.

### Methodological procedures

For this study, the six methodological stages proposed by Mendes, Silveira and Galvão were followed^(9)^, namely: research question formulation; establishment of study inclusion and exclusion criteria; extraction of data from studies; assessment and critical analysis of studies to be included in the review; interpretation of results; and presentation of review/synthesis of knowledge.

Firstly, the following guiding question was formulated: what are telenursing contributions in PHC during the COVID-19 pandemic? For the appropriate formulation, the acronym PICO^(9)^ was used as follows: P (Population) = general population; I (topic of Interest) = telenursing in PHC in the COVID-19 pandemic scenario; C (Comparison) = not applicable; O (Outcome) = contributions from practice.

Primary scientific articles with a qualitative and quantitative approach, published from January 2020 to August 2022, in Portuguese, English and Spanish, that answered the research question, were included. Reviews, editorials, letters, opinion articles, comments, abstracts of annals, duplicate publications, dossiers, course completion works, official documents from national and international programs, experience reports, reflection studies, theoretical studies, theses, dissertations, epidemiological bulletins, management reports and books were excluded.

### Data source

As for data sources, the Public Medical Literature Analysis and Retrieval System Online (PubMed), Cumulative Index to Nursing & Allied Health Literature (CINAHL), Latin American and Caribbean Literature in Health Sciences (LILACS), Nursing Database (BDENF), Scopus, Web of Science (WoS) and EMBASE as well as the virtual library of Scientific Electronic Library Online (SciELO) scientific journals databases were included. Furthermore, the research was supported by the Coordination for the Improvement of Higher Education Personnel (CAPES - *Coordenação de Aperfeiçoamento de Pessoal de Nível Superior*) Journal Portal and the Virtual Health Library (VHL).

The terms used for the research were selected using Health Sciences Descriptors (DeCS) and Medical Subject Headings (Mesh), namely: *Consulta Remota, Telemedicina, Telenfermagem* (*Teleenfermería,* Remote Consultation, Telemedicine, Telenursing); *Enfermagem* (*Enfermeria, Nursing, Nurses*); *Atenção Primária à Saúde* (*Atención Primaria de Salud,* Primary Health Care, *Enfermagem de Atenção Primária, Enfermería de Atención Primaria,* Primary Care Nursing, *Enfermagem Primária, Enfermería Primaria,* Primary Nursing); and COVID-19. To combine the descriptors, the Boolean operators AND and OR were used.

Once the data sources and descriptors were defined, search strategies were developed according to the databases (Chart 1). To this end, the research had the professional assistance of a librarian. Furthermore, it should be noted that the search and selection of articles included in this review were carried out by two reviewers independently.

**Chart 1:** Search strategies for the databases included in the review, 2022

Database	Search strategy
PubMed	((“Remote Consultation”[Mesh] OR “Remote Consultation” OR “Teleconsultation” OR “Asynchronous Teleconsultation” OR “Synchronous Teleconsultation” OR “Telemedicine”[Mesh] OR “Telemedicine” OR “Telenursing”[Mesh] OR “Telenursing” OR “Telecare” OR “Telecure” OR “Telehealth” OR Tele^*^[Title/Abstract]) AND (“Nursing”[Mesh] OR “Nursing”[Title/Abstract] OR “Nurse”[Title/Abstract] OR “Nurses”[Mesh] OR “Nurses”[Title/Abstract] OR Nurs^*^[Title/Abstract]) AND (“Primary Health Care”[Mesh] OR “Primary Health Care” OR “Primary Healthcare” OR “Primary Care” OR “Basic Health Care” OR “Basic Care” OR “Basic Service” OR “Primary Care Nursing”[Mesh] OR “Primary Care Nursing” OR “Primary Nursing”[Mesh] OR “Primary Nursing”) AND (“Coronavirus Infections”[Mesh] OR “Coronavirus Infections” OR “Coronavirus”[Mesh] OR “Coronavirus” OR “SARS Virus”[Mesh] OR “SARS Virus” OR “SARS-CoV” OR “COVID-19”[Mesh] OR “COVID-19” OR “SARS-CoV-2”[Mesh] OR “SARS-CoV-2” OR “SARSCoV2” OR “SARS2” OR “COVID19” OR “COVID-2019” OR “COVID 2019” OR “SARS COV 2” OR “2019-nCoV” OR “2019ncov” OR “nCoV 2019”))
CINAHL	(((MH “Remote Consultation”+) OR “Remote Consultation” OR “Teleconsultation” OR “Asynchronous Teleconsultation” OR “Synchronous Teleconsultation” OR (MH Telemedicine+) OR “Telemedicine” OR (MH “Telenursing”+) OR “Telenursing” OR “Telecare” OR “Telecure” OR “Telehealth” OR (TI Tele^*^ OR AB Tele^*^)) AND ((MH “Nursing”+) OR (TI “Nursing” OR AB “Nursing”) OR (TI “Nurse” OR AB “Nurse”) OR (MH “Nurses”+) OR (TI “Nurses” OR AB “Nurses”) OR (TI Nurs^*^ OR AB Nurs^*^)) AND ((MH “Primary Health Care”+) OR “Primary Health Care” OR “Primary Healthcare” OR “Primary Care” OR “Basic Health Care” OR “Basic Care” OR “Basic Service” OR (MH “Primary Care Nursing”+) OR “Primary Care Nursing” OR (MH “Primary Nursing”+) OR “Primary Nursing” ) AND ((MH “Coronavirus Infections”+) OR “Coronavirus Infections” OR (MH “Coronavirus”+) OR “Coronavirus” OR (MH “SARS Virus”+) OR “SARS Virus” OR “SARS-CoV” OR (MH “COVID-19”+) OR “COVID-19” OR (MH “SARS-CoV-2”+) OR “SARS-CoV-2” OR “SARSCoV2” OR “SARS2” OR “COVID19” OR “COVID-2019” OR “COVID 2019” OR “SARS COV 2” OR “2019-nCoV” OR “2019ncov” OR “nCoV 2019” ))
LILACS	((“Consulta Remota” OR “Consulta à Distância” OR “Teleconsulta” OR “Teleconsultoria” OR “Telemedicina” OR “Saúde Digital” OR “e-Saúde” OR “Telessaúde” OR “Teleassistência” OR “Telenfermagem” OR “Teleenfermería” OR “Telesalud” OR “Teleasistencia” OR “eSalud” OR “Telecuidado” OR “Remote Consultation” OR “Remote Consultation” OR “Teleconsultation” OR “Asynchronous Teleconsultation” OR “Synchronous Teleconsultation” OR “Telemedicine” OR “Telemedicine” OR “Telenursing” OR “Telenursing” OR “Telecare” OR “Telecure” OR “Telehealth” OR Tele^*^) AND (“Enfermagem” Enfermeir^*^ OR “Enfermeria” OR Enfermer^*^ OR “Nursing” OR “Nurse” OR “Nurses” OR Nurs^*^) AND (“Atenção Primária à Saúde” OR “Atenção Básica” OR “Atenção Primária” OR “Atendimento Básico” OR “Atendimento Primário” OR “Cuidado de Saúde Primário” OR “Cuidado Primário” OR “Cuidado de Saúde Básico” OR “Cuidado Básico” OR “Enfermagem de Atenção Primária” OR “Enfermagem Primária” OR “Enfermaria Primária” OR “Atención Primaria de Salud” OR “Atención Primaria” OR “Atención Básica” OR “Cuidado de la Salud Primarios” OR “Servicio Básico” OR “Servicios Básicos” OR “Enfermería de Atención Primaria” OR “Enfermería Primaria” OR “Primary Health Care” OR “Primary Healthcare” OR “Primary Care” OR “Basic Health Care” OR “Basic Care” OR “Basic Service” OR “Primary Care Nursing” OR “Primary Nursing”) AND (“Infecções por Coronavirus” OR “Vírus da SARS” OR “Infecciones por Coronavirus” OR “Virus del SRAS” OR “Coronavirus Infections” OR “Coronavirus” OR “SARS Virus” OR “SARS-CoV” OR “COVID-19” OR “SARS-CoV-2” OR “SARSCoV2” OR “SARS2” OR “COVID19” OR “COVID-2019” OR “COVID 2019” OR “SARS COV 2” OR “2019-nCoV” OR “2019ncov” OR “nCoV 2019”))
BDENF	((“Consulta Remota” OR “Consulta à Distância” OR “Teleconsulta” OR “Teleconsultoria” OR “Telemedicina” OR “Saúde Digital” OR “e-Saúde” OR “Telessaúde” OR “Teleassistência” OR “Telenfermagem” OR “Teleenfermería” OR “Telesalud” OR “Teleasistencia” OR “eSalud” OR “Telecuidado” OR “Remote Consultation” OR “Remote Consultation” OR “Teleconsultation” OR “Asynchronous Teleconsultation” OR “Synchronous Teleconsultation” OR “Telemedicine” OR “Telemedicine” OR “Telenursing” OR “Telenursing” OR “Telecare” OR “Telecure” OR “Telehealth” OR Tele^*^) AND (“Enfermagem” Enfermeir^*^ OR “Enfermeria” OR Enfermer^*^ OR “Nursing” OR “Nurse” OR “Nurses” OR Nurs^*^) AND (“Atenção Primária à Saúde” OR “Atenção Básica” OR “Atenção Primária” OR “Atendimento Básico” OR “Atendimento Primário” OR “Cuidado de Saúde Primário” OR “Cuidado Primário” OR “Cuidado de Saúde Básico” OR “Cuidado Básico” OR “Enfermagem de Atenção Primária” OR “Enfermagem Primária” OR “Enfermaria Primária” OR “Atención Primaria de Salud” OR “Atención Primaria” OR “Atención Básica” OR “Cuidado de la Salud Primarios” OR “Servicio Básico” OR “Servicios Básicos” OR “Enfermería de Atención Primaria” OR “Enfermería Primaria” OR “Primary Health Care” OR “Primary Healthcare” OR “Primary Care” OR “Basic Health Care” OR “Basic Care” OR “Basic Service” OR “Primary Care Nursing” OR “Primary Nursing”) AND (“Infecções por Coronavirus” OR “Vírus da SARS” OR “Infecciones por Coronavirus” OR “Virus del SRAS” OR “Coronavirus Infections” OR “Coronavirus” OR “SARS Virus” OR “SARS-CoV” OR “COVID-19” OR “SARS-CoV-2” OR “SARSCoV2” OR “SARS2” OR “COVID19” OR “COVID-2019” OR “COVID 2019” OR “SARS COV 2” OR “2019-nCoV” OR “2019ncov” OR “nCoV 2019”))
SciELO	((“Consulta Remota” OR “Consulta à Distância” OR “Teleconsulta” OR “Teleconsultoria” OR “Telemedicina” OR “Saúde Digital” OR “e-Saúde” OR “Telessaúde” OR “Teleassistência” OR “Telenfermagem” OR “Teleenfermería” OR “Telesalud” OR “Teleasistencia” OR “eSalud” OR “Telecuidado” OR “Remote Consultation” OR “Remote Consultation” OR “Teleconsultation” OR “Asynchronous Teleconsultation” OR “Synchronous Teleconsultation” OR “Telemedicine” OR “Telemedicine” OR “Telenursing” OR “Telenursing” OR “Telecare” OR “Telecure” OR “Telehealth” OR Tele^*^) AND (“Enfermagem” Enfermeir^*^ OR “Enfermeria” OR Enfermer^*^ OR “Nursing” OR “Nurse” OR “Nurses” OR Nurs^*^) AND (“Atenção Primária à Saúde” OR “Atenção Básica” OR “Atenção Primária” OR “Atendimento Básico” OR “Atendimento Primário” OR “Cuidado de Saúde Primário” OR “Cuidado Primário” OR “Cuidado de Saúde Básico” OR “Cuidado Básico” OR “Enfermagem de Atenção Primária” OR “Enfermagem Primária” OR “Enfermaria Primária” OR “Atención Primaria de Salud” OR “Atención Primaria” OR “Atención Básica” OR “Cuidado de la Salud Primarios” OR “Servicio Básico” OR “Servicios Básicos” OR “Enfermería de Atención Primaria” OR “Enfermería Primaria” OR “Primary Health Care” OR “Primary Healthcare” OR “Primary Care” OR “Basic Health Care” OR “Basic Care” OR “Basic Service” OR “Primary Care Nursing” OR “Primary Nursing”) AND (“Infecções por Coronavirus” OR “Vírus da SARS” OR “Infecciones por Coronavirus” OR “Virus del SRAS” OR “Coronavirus Infections” OR “Coronavirus” OR “SARS Virus” OR “SARS-CoV” OR “COVID-19” OR “SARS-CoV-2” OR “SARSCoV2” OR “SARS2” OR “COVID19” OR “COVID-2019” OR “COVID 2019” OR “SARS COV 2” OR “2019-nCoV” OR “2019ncov” OR “nCoV 2019”))
Scopus	((“Remote Consultation” OR “Teleconsultation” OR “Asynchronous Teleconsultation” OR “Synchronous Teleconsultation” OR “Telemedicine” OR “Telenursing” OR “Telecare” OR “Telecure” OR “Telehealth” OR Tele^*^) AND (“Nursing” OR “Nurse” OR “Nurses”[Mesh] OR “Nurses” OR Nurs^*^) AND (“Primary Health Care” OR “Primary Healthcare” OR “Primary Care” OR “Basic Health Care” OR “Basic Care” OR “Basic Service” OR “Primary Care Nursing” OR “Primary Nursing”) AND (“Coronavirus Infections” OR “Coronavirus” OR “SARS Virus” OR “SARS-CoV” OR “COVID-19” OR “SARS-CoV-2” OR “SARSCoV2” OR “SARS2” OR “COVID19” OR “COVID-2019” OR “COVID 2019” OR “SARS COV 2” OR “2019-nCoV” OR “2019ncov” OR “nCoV 2019”))
WoS	((“Remote Consultation” OR “Teleconsultation” OR “Asynchronous Teleconsultation” OR “Synchronous Teleconsultation” OR “Telemedicine” OR “Telenursing” OR “Telecare” OR “Telecure” OR “Telehealth” OR Tele^*^) AND (“Nursing” OR “Nurse” OR “Nurses”[Mesh] OR “Nurses” OR Nurs^*^) AND (“Primary Health Care” OR “Primary Healthcare” OR “Primary Care” OR “Basic Health Care” OR “Basic Care” OR “Basic Service” OR “Primary Care Nursing” OR “Primary Nursing”) AND (“Coronavirus Infections” OR “Coronavirus” OR “SARS Virus” OR “SARS-CoV” OR “COVID-19” OR “SARS-CoV-2” OR “SARSCoV2” OR “SARS2” OR “COVID19” OR “COVID-2019” OR “COVID 2019” OR “SARS COV 2” OR “2019-nCoV” OR “2019ncov” OR “nCoV 2019”))
EMBASE	((‘Remote Consultation’/exp OR ‘Remote Consultation’ OR ‘Teleconsultation’ OR ‘Asynchronous Teleconsultation’ OR ‘Synchronous Teleconsultation’ OR ‘Telemedicine’/exp OR ‘Telemedicine’ OR ‘Telenursing’/exp OR ‘Telenursing’ OR ‘Telecare’ OR ‘Telecure’ OR ‘Telehealth’ OR Tele^*^:ti,ab) AND (‘Nursing’/exp OR ‘Nursing’:ti,ab OR ‘Nurse’:ti,ab OR ‘Nurses’/exp OR ‘Nurses’:ti,ab OR Nurs^*^:ti,ab) AND (‘Primary Health Care’/exp OR ‘Primary Health Care’ OR ‘Primary Healthcare’ OR ‘Primary Care’ OR ‘Basic Health Care’ OR ‘Basic Care’ OR ‘Basic Service’ OR ‘Primary Care Nursing’/exp OR ‘Primary Care Nursing’ OR ‘Primary Nursing’/exp OR ‘Primary Nursing’) AND (‘Coronavirus Infections’/exp OR ‘Coronavirus Infections’ OR ‘Coronavirus’/exp OR ‘Coronavirus’ OR ‘SARS Virus’/exp OR ‘SARS Virus’ OR ‘SARS-CoV’ OR ‘COVID-19’/exp OR ‘COVID-19’ OR ‘SARS-CoV-2’/exp OR ‘SARS-CoV-2’ OR ‘SARSCoV2’ OR ‘SARS2’ OR ‘COVID19’ OR ‘COVID-2019’ OR ‘COVID 2019’ OR ‘SARS COV 2’ OR ‘2019-nCoV’ OR ‘2019ncov’ OR ‘nCoV 2019’))

### Data collection and organization

Data collection took place in August 2022, and the studies found were exported, organized and stored with the help of Microsoft Excel^®^. Furthermore, this review used the Preferred Reporting Items for Systematic Reviews and Meta-Analyses (PRISMA) Flow Diagram model in an adapted form^(10)^.

**Figure 1:**
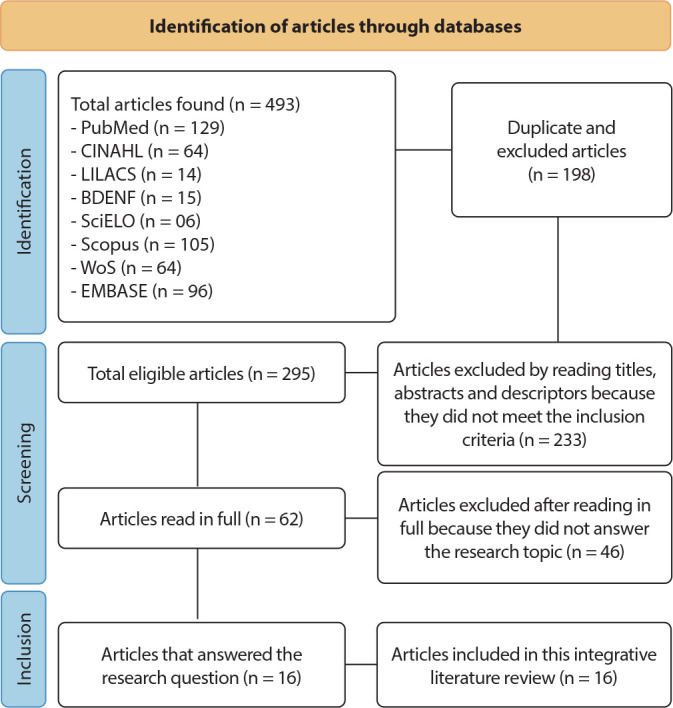
Flowchart of study selection steps anchored in PRISMA, 2022

In the first selection, 493 articles were found. Of this total, 198 duplicates were excluded, leaving 295 articles for reading titles, abstracts and descriptors. After reading, a further 233 articles were excluded as they did not meet the inclusion criteria. At the end of this process, 62 articles were selected as eligible, which were read in full. Finally, another 46 articles were excluded for not answering the research topic, with 16 articles being selected for analysis (Figure 1).

From now on, in the third stage, data was extracted from the selected studies, which were synthesized and will be presented in a synoptic table during this review. In the fourth stage, for assessment and critical analysis of studies included in this review, explanations were sought for the data obtained, using questions such as: what is the objective of the study? Who were the participants and what environment they were in? Did the study achieve its objectives? What are the main results? What are the contributions of telenursing?

In the fifth stage, the data obtained was interpreted with a focus on its main contributions to the topic of this review, thus enabling synthesis of findings and categorization of data, at which point they were divided into three thematic categories, mentioned later in the results.

Finally, in the sixth and final stage, to present synthesis of knowledge, the data were presented descriptively, according to their categories, and discussed in light of relevant scientific literature.

### Data analysis

For data analysis, the articles were read in full, thus allowing organized and coherent data synthesis and presentation in relation to the objectives proposed for this review. During this process, as a way of organizing the data during reading, two charts and a figure were created, highlighting in their content the main results relevant to the respective topics emerging from the studies analyzed.

## RESULTS

### Synthesis of selected studies

The 16 articles selected for this review were distributed in the Scopus (4; 25%), PubMed/MEDLINE (3; 18.75%), CINAHL (3; 18.75%), WoS (2; 12.5%), EMBASE (2; 12.5%), BDENF (1; 6.25%), SciELO (1; 6.25%) and LILACS (0) databases. Furthermore, the countries in which the studies were published included the United States of America (USA) (6; 37.5%), Brazil (3; 18.75%), Belgium (1; 6.25%), Spain (1; 6.25%), Australia (1; 6.25%), Canada (1; 6.25%), Portugal (1; 6.25%), United Kingdom (1; 6.25%) and England (1; 6.25%).

In relation to study language, the majority were published in English (12; 75%), three in Portuguese (18.75%), and one in Spanish (6.25%). Regarding the years of publication, one article was published in 2020 (6.25%), ten in 2021 (62.5%), and five in 2022 (31.25%). Referring to study design, eight are of qualitative (50%), six are quantitative (37.5), one is mixed-methods (6.25%), and one of the studies used both a quantitative and a qualitative approach (6.25%).

With regard to research participants, in their entirety, they were divided between healthcare professionals, patients (children, adults, women and older adults, aged 0 to 110 years, men and women) and caregivers. Thus, nine studies had healthcare professionals as participants (56.25%); four studies included patients as participants (25%); two studies included both healthcare professionals and patients (12.5%); and one study included patients and caregivers (6.25%).

In all the articles selected, it is worth noting that the majority referred to the PHC multidisciplinary team. In a comprehensive description of participants, healthcare professionals comprised nurses, physicians, nutritionists, nursing coordinators, medical directors, social workers, social work coordinators, physical therapists, midwives, physician assistants, nursing assistants, program managers, advanced practice professionals, and healthcare assistants.

Furthermore, a chart was created to present the results of the reviews as a way of synthesizing information and gathering the main evidence, organized into four topics, namely: title; year/country; design/number of participants; and interventions in telenursing practice (Chart 2).

**Chart 2:** Description of studies included in the integrative review, 2022

Title	Year / country	Design/number of participants	Interventions in telenursing practice^(5)^
Telenursing in the home care service in COVID-19 pandemic: a cross-sectional study^(11)^	2021 Brazil	Qualitative and quantitative of cross-sectional type n=246	Consultation via voice call in synchronous format.
*Intervenção multiprofissional e telenfermagem no tratamento de obesos na pandemia de COVID-19: ensaio clínico pragmático* ^(12)^	2021 Brazil	Quantitative of pragmatic intervention clinical trial type n=22	Interconsultation, monitoring and health education actions via text, audio and video messages in asynchronous format.
The role of telehealth and clinical informatics in data driven primary care redesign^(13)^	2022 USA	Quantitative of observational type n=12,299	Consultation and interconsultation via voice call in synchronous format.
Patient experience of telemedicine for headache care during the COVID-19 pandemic: An American Migraine Foundation survey study^(14)^	2021 USA	Quantitative of descriptive-exploratory type n=1,160	Consultation and interconsultation via voice call in synchronous format.
The impact of COVID-19 on chronic care according to providers: a qualitative study among primary care practices in Belgium^(15)^	2020 Belgium	Qualitative of descriptive-exploratory type n=21	Consultation and interconsultation via voice call in synchronous format.
“At home, with care”: lessons from New York City home-based primary care practices managing COVID-19^(16)^	2021 USA	Qualitative of cross-sectional type n=13	Consultation, interconsultation and monitoring via voice call in synchronous format.
Using telehealth to deliver primary care to adolescents during and after the COVID-19 pandemic: national survey study of us primary care professionals^(17)^	2021 USA	Quantitative of descriptive-exploratory type n=1,047	Consultation via voice and video call in synchronous format.
*La vídeo-consulta en atención primaria de salud: una experiencia de implantación* ^(18)^	2021 Spain	Qualitative of exploratory-descriptive type n=324	Consultation via voice and video call in synchronous format.
Nursing interventions increase influenza vaccination quality measures for home telehealth patients^(19)^	2022 USA	Quantitative of concurrent cohort type n=513	Interconsultation and monitoring via text messages in asynchronous format.
Meeting the challenges of COVID-19: evaluation of nurse-led changes to telephonic assessment^(20)^	2022 USA	Qualitative of exploratory-descriptive type n=15	Consultation via voice and video call in synchronous format.
*Gestão no cuidado às pessoas com HIV na Atenção Primária à Saúde em tempos do novo coronavírus* ^(21)^	2022 Brazil	Qualitative of exploratory-descriptive type n=12	Consultation via voice call in synchronous format and interconsultation and monitoring via text message in asynchronous format.
Experiences of Australian primary healthcare nurses in using telehealth during COVID-19: a qualitative study^(22)^	2021 Australia	Qualitative of exploratory-descriptive type n=25	Consultation via voice and video call in synchronous format.
*Changes to telehealth practices in primary care in New Brunswick (Canada): a comparative study pre and during the COVID-19 pandemic* ^(23)^	2021 Canada	Quantitative of comparative type n=114	Consultation via voice and video call in synchronous format and consultation via text message in asynchronous format.
Implementation of digital monitoring services during the COVID-19 pandemic for patients with chronic diseases: Design Science Approach^(24)^	2021 Portugal	Qualitative of technological prospecting type n=53	Consultation via voice and video call in synchronous format and monitoring via text message in asynchronous format.
Implementation of remote consulting in UK primary care following the COVID-19 pandemic: a mixed-methods longitudinal study^(25)^	2021 United Kingdom	Quantitative longitudinal of mixed-methods type n=41	Consultation via voice call in synchronous format.
What can general practice learn from primary care nurses’ and healthcare assistants’ experiences of the COVID-19 pandemic? A qualitative study^(26)^	2022 England	Qualitative of exploratory type n=24	Consultation via voice call in synchronous format.

*Note: HIV - Human Immunodeficiency Virus; PHC - Primary Health Care; UK - United Kingdom; USA - United States of America.*

### Synthesis of results

From the analysis of the articles, three thematic categories emerged, namely: Telenursing contributions in Primary Health Care; Gathering positive aspects and aspects that require improvement for telenursing practice in Primary Health Care; and Perspectives for consolidating telenursing in a post-pandemic scenario.

### Telenursing contributions in Primary Health Care

Based on the main objective of this review, in the first category, the main evidence from the total of 16 studies analyzed regarding teleconsultation contributions to nursing in PHC stands out. The studies highlighted a wide diversity of health demands, including acute and chronic problems. From this, various telenursing contributions in PHC were identified, as shown in Chart 3.

**Chart 3:** Evidence about telenursing contributions in Primary Health Care, 2022

Title	Health demands	Contributions of remote consultation in PHC
Telenursing in the home care service in COVID-19 pandemic: a cross-sectional study^(11)^	General care for older adults in HCS	The main interventions were related to maintaining healthcare, social isolation, maintaining physical and mental health and hygiene.
*Intervenção multiprofissional e telenfermagem no tratamento de obesos na pandemia de COVID-19: ensaio clínico pragmático* ^(12)^	Obesity	Multidisciplinary remote intervention and telenursing significantly reduced the risk variables for metabolic syndrome in obesity treatment.
The role of telehealth and clinical informatics in data driven primary care redesign^(13)^	CKD, DM, COPD, heart disease, obesity, asthma, mental disorders, lipid disorders and smoking	The implementation of telehealth supported chronic healthcare management and avoided emergency room visits or the need for hospitalization.
Patient experience of telemedicine for headache care during the COVID-19 pandemic: An American Migraine Foundation survey study^(14)^	Headache	The majority of patients classified their experience with teleconsultation as “very good” (62.1%) for both headache assessment and treatment. The remaining patients classified the experience as “good” (20.7%), “fair” (10.5%), “poor” (3.6%) and “other” (3.1%).
The impact of COVID-19 on chronic care according to providers: a qualitative study among primary care practices in Belgium^(15)^	DM and diseases related to COVID-19	The study shows that the pandemic affected continuity of chronic care, which increased the potential of digital and remote healthcare.
“At home, with care”: lessons from New York City home-based primary care practices managing COVID-19^(16)^	General care for older adults in home care	Practices prioritized open and honest communication with patients and balanced telehealth with the need for hands-on care.
Using telehealth to deliver primary care to adolescents during and after the COVID-19 pandemic: national survey study of us primary care professionals^(17)^	Adolescent health (acute and chronic illnesses, mental and behavioral health, and vaccinations)	The majority of participants agreed that telehealth increases access to care for adolescents (69%).
*La vídeo-consulta en atención primaria de salud: una experiencia de implantación* ^(18)^	Women’s health (skin lesions, pressure ulcers, test results, prenatal care, postpartum assessments and breastfeeding	Video consultation is presented as an emerging way of interacting with patients, saving time and resources, achieving high levels of resolution.
Nursing interventions increase influenza vaccination quality measures for home telehealth patients^(19)^	Vaccination	Interventions regarding remote patient monitoring resulted in an increase in vaccination rates, reaching 70.4% of patients aged 19 to 65 years and 81.7% of patients over 66 years.
Meeting the challenges of COVID-19: evaluation of nurse-led changes to telephonic assessment^(20)^	Screening for signs and symptoms of COVID-19	Telephone triages were seen as beneficial, valuable, and improved patient care.
*Gestão no cuidado às pessoas com HIV na Atenção Primária à Saúde em tempos do novo coronavírus* ^(21)^	HIV	The incorporation of non-in-person care technologies and the facilitation of routines stood out as strategies for expanding access.
Experiences of Australian primary healthcare nurses in using telehealth during COVID-19: a qualitative study^(22)^	General care for women’s health, maternal and child health, mental health and the aboriginal population	Telehealth has provided a means to continue delivery of PHC services during COVID-19.
Changes to telehealth practices in primary care in New Brunswick (Canada): a comparative study pre and during the COVID-19 pandemic^(23)^	General care aimed at family health	The use of telephone consultations increased by 122%, from 43.9% pre-pandemic to 97.6% during the pandemic. The same occurred with the virtual consultation, which went from 19.3% to 41.2%.
Implementation of digital monitoring services during the COVID-19 pandemic for patients with chronic diseases: Design Science Approach^(24)^	Elderly population diagnosed with COPD, heart disease, stroke, asthma, DM, cancer or rheumatic conditions	The total number of participants were able to manage patients remotely in a safe and complete way, with a high degree of satisfaction.
Implementation of remote consulting in UK primary care following the COVID-19 pandemic: a mixed-methods longitudinal study^(25)^	General care aimed at family health	The move to remote consultation has proven successful in reaching and targeting vulnerable patients (older adults and people with mental health disorders).
What can general practice learn from primary care nurses’ and healthcare assistants’ experiences of the COVID-19 pandemic? A qualitative study^(26)^	General care regarding primary care	Judicious implementation of telehealth can help preserve the hands-on, caring nature of nursing.

*Note: HCS - Home Care Service; PHC - Primary Health Care; CKD - Chronic Kidney Disease; DM - Diabetes Mellitus; COPD - Chronic Obstructive Pulmonary Disease; HIV - Human Immunodeficiency Virus; Stroke - Cerebral Vascular Accident; UK - United Kingdom.*

### Gathering positive aspects and aspects that require improvement for telenursing practice in Primary Health Care

In this category, it is proposed to compile the main positive aspects of telenursing in PHC as well as the main aspects that require improvement in practice, considering it a complementary and not a substitute strategy for assistance. Still taking into account the standardization of telenursing, among the six definitions and attributions of practice mediated by ICTs, nursing consultation, interconsultation, nursing monitoring and health education were identified in selected studies (Chart 4).

**Chart 4:** Main positive aspects and aspects that require improvement for telenursing practice in Primary Health Care, 2022

Positive aspects	Continuity of care^(11,15,20-22)^ Strengthens health guidelines^(11,17,25,26)^ Improving the bond between professionals and patients^(11,17)^ Strengthens multidisciplinary and interprofessional service^(16,26)^ Optimizes healthcare^(26)^ Enables the inclusion of new technologies in primary care^(23)^ Improves control of epidemiological data^(24)^ Reduces absenteeism^(23,26)^ Increases vaccination rates^(17,19,26)^ Reduces the risks of infection and disease transmission^(14,27)^ Reduces physical and geographic barriers^(14)^ Provides accessibility to healthcare services^(21,22)^ Strengthens health promotion, prevention and education actions^(12,17,19,25)^ Facilitates diagnosis, medication prescription and exam assessment^(14,20)^ Reduces time and travel costs^(14,17,18,22)^ Provides comfort to patients^(17)^ Prevents hospital overload^(13,20)^ Encourages self-care^(26)^ Improves mental healthcare provision^(16)^
Aspects that require improvement	Difficulty accessing and handling technological resources^(12,14,17,18,20,22)^ Exclusion of patients with problems with physical and cognitive limitations^(14,16,22)^ Need for a caregiver or family member during teleconsultation^(14,16)^ Privacy difficulties at home^(17)^ Need for investment and guidelines^(14,24)^ Prevents physical examination^(17,22,23,26)^ Reduction in screening for infectious diseases and chronic diseases^(21)^ Need for training, practice and professional experience^(21,22,25)^

It is clear that selected studies brought relevant data supported by alternations between pros and cons arising from the experiences of both professionals (nurses/multidisciplinary team) and patients. Furthermore, aspects that require improvement emerge from specific characteristics and demands of certain target audiences.

### Perspectives for consolidating telenursing in a post-pandemic scenario

From a post-pandemic perspective, it is suggested that telenursing, associated with in-person consultations, be implemented in a post-COVID-19 pandemic scenario^(12)^. It is also suggested that it reach not only priority groups, but the population on a large scale, as a way of assessing adherence of different population groups, as telenursing is considered a promising health strategy for healthcare services^(21)^.

Based on the experiences acquired with nursing consultations mediated by ICTs amidst the COVID-19 pandemic, PHC professionals showed a look towards the future, hoping that assistance can be interspersed between in-person and virtual consultations^(22)^, taking into account technological advances and the need for society to use the internet. For these reasons, financial investments from the public and private sectors are necessary, in addition to promoting telenursing as a guarantee of improved access to healthcare in crisis situations^(14,16)^.

Telenursing is seen as an opportunity for future improvement in PHC care, thus preserving the careful and practical nature of nursing, since practice encouraged interprofessional work and communication between health spheres. However, improvements need to come from technological investment, creation of guidelines and professional training^(26)^.

PHC professionals’ interest in maintaining telenursing beyond the COVID-19 pandemic is clear, providing the applicability of this care modality in a scenario without emerging or crisis conditions. The same professionals want ICT-mediated consultations to be carried out in balance with in-person consultations, making it necessary to carry out further studies related to the subject^(23)^.

Furthermore, in times of crisis, technology and digital media have great potential to maintain the employability of professionals, continuing healthcare for the population^(15)^. To achieve this, support and investment is needed from the government, companies, organizations and researchers^(17)^.

## DISCUSSION

The results of this review made it possible to answer the proposed guiding question, strengthening telenursing contributions in PHC amidst the COVID-19 pandemic, in addition to providing perspectives for improving practice and consolidating it in a post-pandemic scenario. Thus, using telenursing within PHC proved to be fundamental for continuity of care in the midst of the pandemic in both acute and chronic health situations, in addition to recurring demands of the population’s care routine^(27)^.

Added to this, remote care allowed both parties involved, professional and patient, to break down geographical and temporal barriers, resolving the distance between the population and healthcare services^(28)^. It turns out that, through telenursing, both nursing and the PHC multidisciplinary team were able to guarantee access to healthcare services and continuity of care for the entire population, among children, adolescents, adults and older adults, regardless of physical and social distancing, meeting the most varied health demands. In fact, telenursing has proven to be an important technological tool for maintaining communication between professionals and patients.

Nursing, together with the PHC interprofessional team, stood out amidst the COVID-19 pandemic as the most important player in combating the spread of the disease, ensuring continuity of assistance to the population amid social distancing and isolation restrictions. Through the implementation of teleconsultation on an emergency basis, guarantee of care was maintained between diagnoses and prevention and health recovery actions. With this, nursing remained on the front line of the pandemic, a moment in which professionals found themselves facing a reality of constant resilience, improvement and adaptation^(29)^.

In view of this, telenursing, through consultation, interconsultation, monitoring and health education mediated by ICTs, whether through synchronous interaction (voice and/or video consultations) or asynchronous (voice and/or textual messages), emerges as an innovation and transformation of healthcare standards in PHC with regard to care production provided by nursing to users. As a result, telenursing has driven changes in healthcare workflows and management. Furthermore, it influenced the conduct of each professional and patients’ perspective on their health, as it allowed both professionals and patients broad autonomy for care.

Furthermore, telenursing has contributed to the discovery of weaknesses and potentialities regarding healthcare, providing a partnership between professionals and patients in favor of the best path to be taken to improve care. Practice has become a strong ally for communication between PHC and the population, strengthening bonds and strengthening trust between those involved^(30)^.

However, given the rapid expansion of telenursing, it is clear that it is a practice that requires improvement, due to the circumstances of the COVID-19 pandemic scenario^(31)^. It was necessary to pay attention to issues of equity, considering the population that is in a vulnerable situation and that does not have access to adequate technological resources for telenursing practice^(27)^.

In fact, the studies in this review showed that access to technologies was a factor that made it difficult to reach a certain part of the population, because the reality is that not everyone has the resources to use technological resources appropriately. These difficulties are present from patients’ cognitive and/or physical capacity and socioeconomic situation to their availability of adequate technological resources for telenursing applicability. Added to this, the need for in-person consultations was present, both for professionals and patients, due to insecurities related to the physical examination.

To this end, telenursing is an opportunity to improve healthcare services and an evolution for relationships between professionals and patients, although it will require investments for the future so that the purposes are achieved and the quality of care is maintained^(31)^.

### Study limitations

This study has a limitation regarding time frame, as only studies published between January 2020 and August 2022 were included in the review, considering the recent COVID-19 pandemic period. Furthermore, the articles selected for this review are limited to the reality of only a few countries and continents, since there are different cultures and health systems.

We recognize that other studies, with other perspectives, will be of great importance to understand the phenomenon of digital health and telenursing within PHC, thus guaranteeing improvements to healthcare.

### Contributions to nursing, health or public policy

It is considered that the present study contributed to identifying the potentialities and weaknesses of telenursing within PHC. Furthermore, this study sought to provide scientific support for both care practice and new research on the subject, since nursing, together with the interprofessional team, needs to strengthen itself in the face of current needs of contemporary society and technological evolution.

## FINAL CONSIDERATIONS

This integrative review made it possible to identify telenursing contributions in PHC during the COVID-19 pandemic, making it possible to compile the main positive aspects and aspects that require improvement for practice, in addition to providing reflection on their consolidation in a post-pandemic scenario.

Even in the face of an epidemiological crisis, PHC underwent emergency modifications and adaptations, ensuring, through telenursing, the population’s access to healthcare services, maintaining education, health promotion and recovery actions, in addition to disease prevention actions. Furthermore, continuity of care was maintained, even in the face of the physical and social distancing imposed by the pandemic. Telenursing was and is being an essential technological resource to expand access to primary healthcare services, presenting more advantages than disadvantages, both for professionals and patients.

Furthermore, the main positive points of telenursing were continuity of care, strengthening of health guidelines, increase in vaccination rates, strengthening of health promotion, prevention and education actions, and reduction of time and travel costs. The main aspects that require improvement for practice were identified as the difficulty in accessing and handling technological resources and the impossibility of performing physical examination.

It is considered that digital health technologies have proven to be an integral part of current contemporary society, requiring a careful and critical look at technological advances.

## Supplementary Material









## Data Availability

*https://doi.org/10.48331/scielodata.GVWFPA*

## References

[1] Ministério da Economia (BR), Instituto de Pesquisa Econômica Aplicada (IPEA), Centro de Pesquisa em Ciência, Tecnologia e Sociedade (2021). Novas tecnologias e normatização ampliam espaço para telessaúde no Brasil.

[2] World Health Organization (WHO) (2021). Global strategy on digital health 2020-2025.

[3] Ministério da Saúde (BR) (2020). Estratégia de saúde digital para o Brasil 2020-2028.

[4] Santos WS, Sousa JH, Soares JC, Raasch M. (2020). Reflexões acerca do uso da telemedicina no Brasil: oportunidade ou ameaça?. Rev Gest Sist Saúde - RGSS.

[5] Organização Pan-Americana da Saúde (OPAS) (2020). Teleconsulta durante uma pandemia: kit de ferramentas de transformação digital-ferramentas de conhecimento.

[6] Conselho Federal de Enfermagem (COFEN) (2020). Resolução nº 634, de 26 de março de 2020. Autoriza e normatiza a teleconsulta de enfermagem como forma de combate à pandemia provocada pelo novo coronavírus (Sars-Cov-2).

[7] Conselho Federal de Enfermagem (COFEN) (2022). Resolução nº 696/2022, alterada pela Resolução nº 707/2022. Dispõe sobre a atuação da enfermagem na saúde digital, normatizando a telenfermagem.

[8] Nunciaroni AT, Cunha CLF, Borges FA, Souza IL, Koster I, Souza IS (2022). Enfermagem na APS: contribuições, desafios e recomendações para o fortalecimento da Estratégia Saúde da Família. APS Rev.

[9] Mendes KDS, Silveira RCCP, Galvão CM. (2019). Uso de gerenciador de referências bibliográficas na seleção dos estudos primários em revisão integrativa. Texto Contexto Enferm.

[10] McKenzie MJ, McKenzie JE, Bossuyt PM, Boutron I, Hoffmann TC, Mulrow CD (2021). A declaração PRISMA 2020: uma diretriz atualizada para relatar revisões sistemáticas. PLOS Med.

[11] Rodrigues MA, Santana RF, Hercules AB, Bela JC, Rodrigues JN. (2021). Telenursing in the Home Care Service in Covid-19 pandemic: a cross-sectional study. Online Braz J Nurs.

[12] Christinelli HCB, Westphal G, Costa MAR, Okawa RTP, Nardo N, Fernandes CAM. (2021). Intervenção multiprofissional e telenfermagem no tratamento de obesos na pandemia de Covid-19: ensaio clínico pragmático. Rev Bras Enferm.

[13] Brown JL, Hewner S. (2022). The role of telehealth and clinical informatics in data driven primary care redesign. J Inform Nurs.

[14] Chiang CC (2021). Patient experience of telemedicine for headache care during the Covid-19 pandemic: an American Migraine Foundation survey study. Headache.

[15] Danhieux K, Buffel V, Pairon A, Benkheil A, Remmen R, Wouters E (2020). The impact of Covid-19 on chronic care according to providers: a qualitative study among primary care practices in Belgium. BMC Fam Pract.

[16] Franzosa E, Gorbenko K, Brody AA, Leff B, Ritchie CS, Kinosian B (2021). “At home, with care”: lessons from New York City home-based primary care practices managing COVID-19. J Am Geriatr.

[17] Gilkey MB, Kong WY, Huang Q, Grabert BK, Thompson P, Brewer NT. (2021). Using Telehealth to Deliver Primary Care to Adolescents During and After the COVID-19 Pandemic: national survey study of US Primary Care Professionals. J Med Internet Res.

[18] Marrero NJ, Brito-Brito PR, Fernández-Gutiérrez DÁ, Sáez Rodríguez MJ, Martínez ACE, Galdona LI (2021). La vídeo-consulta en atención primaria de salud: una experiencia de implantación. Rev Ene Enferm.

[19] Rand ML. (2022). Nursing interventions increase influenza vaccination quality measures for home telehealth patients. J Nurs Care Qual.

[20] Squeri B, Gayton M, Huang J, Chavez S, Souffront K. (2022). Meeting the challenges of Covid-19: evaluation of nurse-led changes to telephonic assessment. Home Healthc Now.

[21] Celuppi IC, Meirelles BHS, Lanzoni GMM, Geremia DS, Metelski FK. (2022). Gestão no cuidado às pessoas com HIV na Atenção Primária à Saúde em tempos do novo coronavírus. Rev Saúde Pública.

[22] James S, Ashley C, Williams A, Desborough J, Mcinnes S, Calma K (2021). Experiences of Australian primary healthcare nurses in using telehealth during Covid-19: a qualitative study. BMJ Open.

[23] Johnson C, Dupuis JB, Goguen P, Grenier G. (2021). Changes to telehealth practices in primary care in New Brunswick (Canada): a comparative study pre and during the Covid-19 pandemic. PLOS ONE.

[24] Lapão LV, Peyroteo M, Maia M, Seixas J, Gregório J, Mira da Silva M (2021). Implementation of digital monitoring services during the Covid-19 pandemic for patients with chronic diseases: design science approach. J Med Internet Res.

[25] Murphy M, Scott LJ, Salisbury C, Turner A, Scott A, Denholmet R (2021). Implementation of remote consulting in UK primary care following the Covid-19 pandemic: a mixed-methods longitudinal study. Brit J Gen Pract.

[26] Russell A, Wildt G, Grut M, Greenfield S, Clarke J. (2022). What can general practice learn from primary care nurses’ and healthcare assistants’ experiences of the Covid-19 pandemic? a qualitative study. BMJ Open.

[27] Lana LD, Silva MCS, Tanaka AKSR, Vieira RW, Rosa LGF, Aires M. (2020). Teleconsulta de enfermagem aplicações para pessoas idosas na pandemia de COVID-19. enfermagem gerontologica no cuidado do idoso em tempos da Covid-19.

[28] Nejadshafiee M, Bahaadinbeigy K, Kazemi M, Nekoei-Moghadam M. (2020). Telenursing: a step for care management in disaster and emergencies. J Educ Health Promot.

[29] Rodrigues MA, Hercules ABS, Gnatta JR, Coelho JC, Mota ANB, Pierin AMG (2022). Teleconsultation as an advanced practice nursing during the Covid-19 pandemic based on Roy and Chick-Meleis. Rev Esc Enferm USP.

[30] Kord Z, Fereidouni Z, Mirzaee MS. (2024). Telenursing home care and Covid-19: a qualitative study. BMJ Support Palliat Care.

[31] Coutinho JSL, Souza SM, Macedo MAA, Domingos CS, Souza LM, Paz DD (2022). A assistência de enfermagem a partir da consulta remota: revisão de literatura. REAS.

