# Supplementary material for: Telenursing contributions in Primary Health Care in the COVID-19 pandemic context: an integrative review
Source: Rev Bras Enferm. 2024 Nov 22;77(5):e20240093. doi: 10.1590/0034-7167-2024-0093 (PMC11653881; doi:10.1590/0034-7167-2024-0093)
Supplement: Supplementary file 1 [file 0034-7167-reben-77-05-e20240093-suppl01.pdf]

PESQUISA REALIZADA EM 8 BASES DE DADOS CIENTÍFICAS

DESCRITORES UTILIZADOS: Consulta Remota, Telemedicina, Telenfermagem (Teleenfermería, Remote Consultation, Telemedicine, Telenursing); Enfermagem (Enfermeria, Nursing, Nurses); Atenção Primária à Saúde (Atención Primaria de Salud, Primary Health Care, Enfermagem de Atenção Primária, Enfermería de Atención Primaria, Primary Care Nursing, Enfermagem Primária, Enfermería Primaria, Primary Nursing); e Covid-19.

FILTROS UTILIZADOS: idiomas português, inglês e espanhol; corte temporal de 2020 a 2022.

BASE DE DADOS	CÓDIGO DE BUSCA	TOTAL DE ARTIGOS ENCONTRADOS
**PUBMED**	(("Remote Consultation"[Mesh] OR "Remote Consultation" OR "Teleconsultation" OR "Asynchronous Teleconsultation" OR "Synchronous Teleconsultation" OR "Telemedicine"[Mesh] OR "Telemedicine" OR "Telenursing"[Mesh] OR "Telenursing" OR "Telecare" OR "Telecure" OR "Telehealth" OR Tele*[Title/Abstract]) AND ("Nursing"[Mesh] OR "Nursing"[Title/Abstract] OR "Nurse"[Title/Abstract] OR "Nurses"[Mesh] OR "Nurses"[Title/Abstract] OR Nurs*[Title/Abstract]) AND ("Primary Health Care"[Mesh] OR "Primary Health Care" OR "Primary Healthcare" OR "Primary Care" OR "Basic Health Care" OR "Basic Care" OR "Basic Service" OR "Primary Care Nursing"[Mesh] OR "Primary Care Nursing" OR "Primary Nursing"[Mesh] OR "Primary Nursing") AND ("Coronavirus Infections"[Mesh] OR "Coronavirus Infections" OR "Coronavirus"[Mesh] OR "Coronavirus" OR "SARS Virus"[Mesh] OR "SARS Virus" OR "SARS-CoV" OR "COVID-19"[Mesh] OR "COVID-19" OR "SARS-CoV-2"[Mesh] OR "SARS-CoV-2" OR "SARSCoV2" OR "SARS2" OR "COVID19" OR "COVID-2019" OR "COVID 2019" OR "SARS COV 2" OR "2019-nCoV" OR "2019ncov" OR "nCoV 2019"))	129
**CINAHL**	(((MH "Remote Consultation"+) OR "Remote Consultation" OR "Teleconsultation" OR "Asynchronous Teleconsultation" OR "Synchronous Teleconsultation" OR (MH Telemedicine+) OR "Telemedicine" OR (MH "Telenursing"+) OR "Telenursing" OR "Telecare" OR "Telecure" OR "Telehealth" OR (TI Tele* OR AB Tele*)) AND ((MH "Nursing"+) OR (TI "Nursing" OR AB "Nursing") OR (TI "Nurse" OR AB "Nurse") OR (MH "Nurses"+) OR (TI "Nurses" OR AB "Nurses") OR (TI Nurs* OR AB Nurs*)) AND ((MH "Primary Health Care"+) OR "Primary Health Care" OR "Primary Healthcare" OR "Primary Care" OR "Basic Health Care" OR "Basic Care" OR "Basic Service" OR (MH "Primary Care Nursing"+) OR "Primary Care Nursing" OR (MH "Primary Nursing"+) OR "Primary Nursing") AND ((MH "Coronavirus Infections"+) OR "Coronavirus Infections" OR (MH "Coronavirus"+) OR "Coronavirus" OR (MH "SARS Virus"+) OR "SARS Virus" OR "SARS-CoV" OR (MH "COVID-19"+) OR "COVID-19" OR (MH "SARS-CoV-2"+) OR "SARS-CoV-2" OR "SARSCoV2" OR "SARS2" OR "COVID19" OR "COVID-2019" OR "COVID 2019" OR "SARS COV 2" OR "2019-nCoV" OR "2019ncov" OR "nCoV 2019"))	64

**LILACS**	(("Consulta Remota" OR "Consulta à Distância" OR "Teleconsulta" OR "Teleconsultoria" OR "Telemedicina" OR "Saúde Digital" OR "e-Saúde" OR "Telessaúde" OR "Teleassistência" OR "Telenfermagem" OR "Teleenfermería" OR "Telesalud" OR "Teleasistencia" OR "eSalud" OR "Telecuidado" OR "Remote Consultation" OR "Remote Consultation" OR "Teleconsultation" OR "Asynchronous Teleconsultation" OR "Synchronous Teleconsultation" OR "Telemedicine" OR "Telemedicine" OR "Telenursing" OR "Telenursing" OR "Telecare" OR "Telecure" OR "Telehealth" OR Tele*) AND ("Enfermagem" Enfermeir* OR "Enfermeria" OR Enfermer* OR "Nursing" OR "Nurse" OR "Nurses" OR Nurs*) AND ("Atenção Primária à Saúde" OR "Atenção Básica" OR "Atenção Primária" OR "Atendimento Básico" OR "Atendimento Primário" OR "Cuidado de Saúde Primário" OR "Cuidado Primário" OR "Cuidado de Saúde Básico" OR "Cuidado Básico" OR "Enfermagem de Atenção Primária" OR "Enfermagem Primária" OR "Enfermaria Primária" OR "Atención Primaria de Salud" OR "Atención Primaria" OR "Atención Básica" OR "Cuidado de la Salud Primarios" OR "Servicio Básico" OR "Servicios Básicos" OR "Enfermería de Atención Primaria" OR "Enfermería Primaria" OR "Primary Health Care" OR "Primary Healthcare" OR "Primary Care" OR "Basic Health Care" OR "Basic Care" OR "Basic Service" OR "Primary Care Nursing" OR "Primary Nursing") AND ("Infecções por Coronavirus" OR "Vírus da SARS" OR "Infecciones por Coronavirus" OR "Virus del SRAS" OR "Coronavirus Infections" OR "Coronavirus" OR "SARS Virus" OR "SARS-CoV" OR "COVID-19" OR "SARS-CoV-2" OR "SARSCoV2" OR "SARS2" OR "COVID19" OR "COVID-2019" OR "COVID 2019" OR "SARS COV 2" OR "2019-nCoV" OR "2019ncov" OR "nCoV 2019"))	14

**BDENF**	(("Consulta Remota" OR "Consulta à Distância" OR "Teleconsulta" OR "Teleconsultoria" OR "Telemedicina" OR "Saúde Digital" OR "e-Saúde" OR "Telessaúde" OR "Teleassistência" OR "Telenfermagem" OR "Teleenfermería" OR "Telesalud" OR "Teleasistencia" OR "eSalud" OR "Telecuidado" OR "Remote Consultation" OR "Remote Consultation" OR "Teleconsultation" OR "Asynchronous Teleconsultation" OR "Synchronous Teleconsultation" OR "Telemedicine" OR "Telemedicine" OR "Telenursing" OR "Telenursing" OR "Telecare" OR "Telecure" OR "Telehealth" OR Tele*) AND ("Enfermagem" Enfermeir* OR "Enfermeria" OR Enfermer* OR "Nursing" OR "Nurse" OR "Nurses" OR Nurs*) AND ("Atenção Primária à Saúde" OR "Atenção Básica" OR "Atenção Primária" OR "Atendimento Básico" OR "Atendimento Primário" OR "Cuidado de Saúde Primário" OR "Cuidado Primário" OR "Cuidado de Saúde Básico" OR "Cuidado Básico" OR "Enfermagem de Atenção Primária" OR "Enfermagem Primária" OR "Enfermaria Primária" OR "Atención Primaria de Salud" OR "Atención Primaria" OR "Atención Básica" OR "Cuidado de la Salud Primarios" OR "Servicio Básico" OR "Servicios Básicos" OR "Enfermería de Atención Primaria" OR "Enfermería Primaria" OR "Primary Health Care" OR "Primary Healthcare" OR "Primary Care" OR "Basic Health Care" OR "Basic Care" OR "Basic Service" OR "Primary Care Nursing" OR "Primary Nursing") AND ("Infecções por Coronavirus" OR "Vírus da SARS" OR "Infecciones por Coronavirus" OR "Virus del SRAS" OR "Coronavirus Infections" OR "Coronavirus" OR "SARS Virus" OR "SARS-CoV" OR "COVID-19" OR "SARS-CoV-2" OR "SARSCoV2" OR "SARS2" OR "COVID19" OR "COVID-2019" OR "COVID 2019" OR "SARS COV 2" OR "2019-nCoV" OR "2019ncov" OR "nCoV 2019"))	15
**SCIELO**	(("Consulta Remota" OR "Consulta à Distância" OR "Teleconsulta" OR "Teleconsultoria" OR "Telemedicina" OR "Saúde Digital" OR "e-Saúde" OR "Telessaúde" OR "Teleassistência" OR "Telenfermagem" OR "Teleenfermería" OR "Telesalud" OR "Teleasistencia" OR "eSalud" OR "Telecuidado" OR "Remote Consultation" OR "Remote Consultation" OR "Teleconsultation" OR "Asynchronous Teleconsultation" OR "Synchronous Teleconsultation" OR "Telemedicine" OR "Telemedicine" OR "Telenursing" OR "Telenursing" OR "Telecare" OR "Telecure" OR "Telehealth" OR Tele*) AND ("Enfermagem" Enfermeir* OR "Enfermeria" OR Enfermer* OR "Nursing" OR "Nurse" OR "Nurses" OR Nurs*) AND ("Atenção Primária à Saúde" OR "Atenção Básica" OR "Atenção Primária" OR "Atendimento Básico" OR "Atendimento Primário" OR "Cuidado de Saúde Primário" OR "Cuidado Primário" OR "Cuidado de Saúde Básico" OR "Cuidado Básico" OR "Enfermagem de Atenção Primária" OR "Enfermagem Primária" OR "Enfermaria Primária" OR "Atención Primaria de Salud" OR "Atención Primaria" OR "Atención Básica" OR "Cuidado de la Salud Primarios" OR "Servicio Básico" OR "Servicios Básicos" OR "Enfermería de Atención Primaria" OR "Enfermería Primaria" OR "Primary Health Care" OR "Primary Healthcare" OR "Primary Care" OR "Basic Health Care" OR "Basic Care" OR "Basic Service" OR "Primary Care Nursing" OR "Primary Nursing") AND ("Infecções por Coronavirus" OR "Vírus da SARS" OR "Infecciones por Coronavirus" OR "Virus del SRAS" OR "Coronavirus Infections" OR "Coronavirus" OR "SARS Virus" OR "SARS-CoV" OR "COVID-19" OR "SARS-CoV-2" OR "SARSCoV2" OR "SARS2" OR "COVID19" OR "COVID-2019" OR "COVID 2019" OR "SARS COV 2" OR "2019-nCoV" OR "2019ncov" OR "nCoV 2019"))	6

**SCOPUS**	(("Remote Consultation" OR "Teleconsultation" OR "Asynchronous Teleconsultation" OR "Synchronous Teleconsultation" OR "Telemedicine" OR "Telenursing" OR "Telecare" OR "Telecure" OR "Telehealth" OR Tele*) AND ("Nursing" OR "Nurse" OR "Nurses"[Mesh] OR "Nurses" OR Nurs*) AND ("Primary Health Care" OR "Primary Healthcare" OR "Primary Care" OR "Basic Health Care" OR "Basic Care" OR "Basic Service" OR "Primary Care Nursing" OR "Primary Nursing") AND ("Coronavirus Infections" OR "Coronavirus" OR "SARS Virus" OR "SARS-CoV" OR "COVID-19" OR "SARS-CoV-2" OR "SARSCoV2" OR "SARS2" OR "COVID19" OR "COVID-2019" OR "COVID 2019" OR "SARS COV 2" OR "2019-nCoV" OR "2019ncov" OR "nCoV 2019"))	105
**WOS**	(("Remote Consultation" OR "Teleconsultation" OR "Asynchronous Teleconsultation" OR "Synchronous Teleconsultation" OR "Telemedicine" OR "Telenursing" OR "Telecare" OR "Telecure" OR "Telehealth" OR Tele*) AND ("Nursing" OR "Nurse" OR "Nurses"[Mesh] OR "Nurses" OR Nurs*) AND ("Primary Health Care" OR "Primary Healthcare" OR "Primary Care" OR "Basic Health Care" OR "Basic Care" OR "Basic Service" OR "Primary Care Nursing" OR "Primary Nursing") AND ("Coronavirus Infections" OR "Coronavirus" OR "SARS Virus" OR "SARS-CoV" OR "COVID-19" OR "SARS-CoV-2" OR "SARSCoV2" OR "SARS2" OR "COVID19" OR "COVID-2019" OR "COVID 2019" OR "SARS COV 2" OR "2019-nCoV" OR "2019ncov" OR "nCoV 2019"))	64
**EMBASE**	(('Remote Consultation'/exp OR 'Remote Consultation' OR 'Teleconsultation' OR 'Asynchronous Teleconsultation' OR 'Synchronous Teleconsultation' OR 'Telemedicine'/exp OR 'Telemedicine' OR 'Telenursing'/exp OR 'Telenursing' OR 'Telecare' OR 'Telecure' OR 'Telehealth' OR Tele*:ti,ab) AND ('Nursing'/exp OR 'Nursing':ti,ab OR 'Nurse':ti,ab OR 'Nurses'/exp OR 'Nurses':ti,ab OR Nurs*:ti,ab) AND ('Primary Health Care'/exp OR 'Primary Health Care' OR 'Primary Healthcare' OR 'Primary Care' OR 'Basic Health Care' OR 'Basic Care' OR 'Basic Service' OR 'Primary Care Nursing'/exp OR 'Primary Care Nursing' OR 'Primary Nursing'/exp OR 'Primary Nursing') AND ('Coronavirus Infections'/exp OR 'Coronavirus Infections' OR 'Coronavirus'/exp OR 'Coronavirus' OR 'SARS Virus'/exp OR 'SARS Virus' OR 'SARS-CoV' OR 'COVID-19'/exp OR 'COVID-19' OR 'SARS-CoV-2'/exp OR 'SARS-CoV-2' OR 'SARSCoV2' OR 'SARS2' OR 'COVID19' OR 'COVID-2019' OR 'COVID 2019' OR 'SARS COV 2' OR '2019-nCoV' OR '2019ncov' OR 'nCoV 2019'))	96

SIGLAS:
BDENF - Base de Dados de Enfermagem
SCIELO - Scientific Electronic Library Online
CINAHL - Cumulative Index to Nursing & Allied Helth Literature
EMBASE - não há nome por extenso
PUBMED - Public Medical Literature Analysis and Retrieval System Online
SCOPUS - não há nome por extenso
WOS - Web of Science
LILACS - Literatura Latino-Americana e do Caribe em Ciências da Saúde

16 ARTIGOS SELECIONADOS

TOTAL DE ARTIGOS ENCONTRADOS = 493
TOTAL DE ARTIGOS DUPLICADOS = 198
TOTAL DE ARTIGOS LIDOS NA ÍNTEGRA = 62
TOTAL DE ARTIGOS SELECIONADOS = 16

QUESTÃO DE PESQUISA: "Quais as contribuições da telenfermagem na Atenção Primária à Saúde no contexto pandêmico da covid-19?"

Todos os artigos foram analisados respeitando os seguintes critérios de inclusão e exclusão:
CRITÉRIOS DE INCLUSÃO: artigos científicos primários de abordagem qualitativa e quantitativa, publicados no período de janeiro de 2020 a agosto de 2022, nos idiomas português, inglês e espanhol, que respondessem à questão de pesquisa.
CRITÉRIOS DE EXCLUSÃO: estudos na modalidade de revisões, editoriais, cartas, artigos de opinião, comentários, resumos de anais, publicações duplicadas, dossiês, trabalhos de conclusão de curso, documentos oficiais de programas nacionais e internacionais, relatos de experiência, estudos de reflexão, estudos teóricos, teses, dissertações, boletins epidemiológicos, relatórios de gestão e livros.

Nº DO ESTUDO IDENTIFICADO NA RIL	BASE DE DADOS	TÍTULO DO ARTIGO	LINK DE ACESSO
E1	BDENF	Telenursing in the home care service in COVID-19 pandemic: a cross-sectional study	https://fi-admin.bvsalud.org/document/view/bvvtw
E2	SCIELO	Multiprofessional intervention and telenursing in the treatment of obese people in the COVID-19 pandemic: a pragmatic clinical trial	https://doi.org/10.1590/0034-7167-2021-0059
E3	CINHAL	The Role of Telehealth and Clinical Informatics in Data Driven Primary Care Redesign	https://pubmed.ncbi.nlm.nih.gov/35733915/
E4	EMBASE	Patient experience of telemedicine for headache care during the Covid-19 pandemic: An American Migraine Foundation survey study	https://doi.org/10.1111/head.14110
E5	PUBMED	The impact of Covid-19 on chronic care according to providers: a qualitative study among primary care practices in Belgium	https://bmcprimcare.biomedcentral.com/articles/10.1186/s12875-020-01326-3
E6	PUBMED	"At home, with care": lessons from New York City home-based primary care practices managing COVID-19	https://pubmed.ncbi.nlm.nih.gov/33179761/
E7	SCOPUS	Using Telehealth to Deliver Primary Care to Adolescents During and After the COVID-19 Pandemic: National Survey Study of US Primary Care Professionals	https://www.jmir.org/2021/9/e31240
E8	CINHAL	La video-consulta en atención primaria de salud: una experiencia de implantación	http://scielo.isciii.es/scielo.php?script=sci_arttext&pid=S1988-348X2021000200002&lng=es
E9	SCOPUS	Nursing interventions increase influenza vaccination quality measures for home telehealth patients	https://pubmed.ncbi.nlm.nih.gov/34224534/
E10	CINHAL	Meeting the Challenges of Covid-19: Evaluation of Nurse-Led Changes to Telephonic Assessment	https://journals.lww.com/homehealthcarenurseonline/Abstract/2022/07000/Meeting_the_Challenges_of_COVID_19__Evaluation_of.7.aspx
E11	EMBASE	Gestão no cuidado às pessoas com HIV na Atenção Primária à Saúde em tempos do novo coronavírus	https://www.revistas.usp.br/rsp/article/view/196465
E12	WOS	Experiences of australian primary healthcare nurses in using telehealth during Covid-19: a qualitative study	https://pubmed.ncbi.nlm.nih.gov/34362804/
E13	SCOPUS	Changes to telehealth practices in primary care in New Brunswick (Canada): A comparative study pre and during the Covid-19 pandemic	https://doi.org/10.1371/journal.pone.0258839
		Implementation of digital monitoring services [illegible]	

E14	SCOPUS	Implementation of digital monitoring services during the Covid-19 pandemic for patients with chronic diseases: Design Science Approach	https://www.ncbi.nlm.nih.gov/pmc/articles/PMC8396539/
E15	PUBMED	Implementation of remote consulting in UK primary care following the Covid-19 pandemic: a mixed-methods longitudinal study	https://pubmed.ncbi.nlm.nih.gov/33558332/
E16	WOS	What can general practice learn from primary care nurses' and healthcare assistants' experiences of the Covid-19 pandemic? A qualitative study	https://pubmed.ncbi.nlm.nih.gov/35292497/

62 ARTIGOS LIDOS NA ÍNTEGRA

LEGENDA:
Artigo selecionado
Artigo excluído

BASE DE DADOS	TÍTULO DO ARTIGO	LINK DE ACESSO/DOI	AVALIAÇÃO
BDENF	Teleconsulta no serviço de atenção domiciliar na pandemia da COVID-19: estudo transversal	https://fi-admin.bvsalud.org/document/view/bvvtw	Responde a
SCIELO	Intervenção multiprofissional e teleenfermagem no tratamento de pessoas obesas na pandemia de COVID-19: um ensaio clínico pragmático	http://www.scielo.br/scielo.php?script=sci_arttext&pid=S0034-71672022001100214&lang=pt	Estudo quantitativo
	O Papel da Telessaúde e da Informática Clínica no Redesenho da Atenção Primária Orientada por Dados	https://search.ebscohost.com/login.aspx?direct=true&db=c8h&AN=154522952&lang=pt-br&site=ehost-live	Estudo quantitativo
	Resultados de hospitalização entre pacientes com COVID-19 em monitoramento remoto	https://search.ebscohost.com/login.aspx?direct=true&db=c8h&AN=157890783&	Não aborda a temática
	Expansão da telessaúde na atenção primária durante a pandemia de COVID-19: benefícios e barreiras	https://search.ebscohost.com/login.aspx?direct=true&db=c8h&AN=155110078&	Não aborda a temática
	Impacto na atividade assistencial e na saúde dos profissionais de Atenção Primária durante o confinamento	https://search.ebscohost.com/login.aspx?direct=true&db=c8h&AN=152354073&	Não aborda a temática
	"Há algo muito pessoal em ver o rosto de alguém": percepções do provedor de visitas de vídeo na atenção primária domiciliar durante o COVID-19	https://search.ebscohost.com/login.aspx?direct=true&db=c8h&AN=153419779&	Não aborda a temática
	Avaliação da comunicação de estudantes de enfermagem na teleconsulta simulada: um estudo transversal	https://search.ebscohost.com/login.aspx?direct=true&db=c8h&AN=156781830&	Não aborda a temática

CINAHL	Consultas remotas de asma na atenção primária	https://search.ebscohost.com/login.aspx?direct=true&db=c8h&AN=148521996&	Não aborda a temática
	Telessaúde para pacientes com diabetes	https://search.ebscohost.com/login.aspx?direct=true&db=c8h&AN=158121610&	Não aborda a temática
	La video-consulta en atención primaria de salud: una experiencia de implantación	https://search.ebscohost.com/login.aspx?direct=true&db=c8h&AN=153606812&	Estudo qualitativo
	Experiências dos pacientes de "longo COVID" na comunidade e recomendações para melhorar os serviços: uma pesquisa de melhoria da qualidade	https://search.ebscohost.com/login.aspx?direct=true&db=c8h&AN=154384635&	Não aborda a temática
	Experiências dos profissionais da atenção primária durante a primeira onda da pandemia de COVID-19 na Grécia: um estudo qualitativo	https://search.ebscohost.com/login.aspx?direct=true&db=c8h&AN=152227760&	Não aborda a temática
	Enfrentando os Desafios da COVID-19: Avaliação das Mudanças Lideradas por Enfermeiros na Avaliação Telefônica	https://search.ebscohost.com/login.aspx?direct=true&db=c8h&AN=157746222&	Estudo qualitativo
	Avaliação qualitativa da transformação rápida do sistema para visitas de vídeo de atenção primária em um centro médico acadêmico	https://search.ebscohost.com/login.aspx?direct=true&db=c8h&AN=146561390&	Não aborda a temática
	Lançamento de um serviço de telemedicina e linha direta de atendimento primário COVID-19 em todo o estado	10.3122/jabfm.2021.S1.200178	Não aborda a temática
	Utilização de telessaúde em instituições residenciais de cuidados a idosos durante a pandemia de COVID-19: um estudo de coorte retrospectivo na prática geral australiana	10.1177/1357633X221094406	Não aborda a temática
	Protocolo de pesquisa para explorar o uso de vídeo e telefone na clínica geral de Aotearoa Nova Zelândia: considerações para a futura telessaúde	10.1136/bmjhci-2020-100309	Não aborda a temática

WOS	Como o COVID-19 afetou as consultas de clínica geral e a renda: evidência de pesquisa populacional transversal de clínico geral da Irlanda	10.1136/bmjopen-2020-044685	Não aborda a temática
	Iniciando a medicação para transtorno do uso de opióides via telemedicina durante o COVID-19: implicações para as reformas propostas para a Lei Ryan Haight	10.1007/s11606-021-07174-w	Não aborda a temática
	O efeito adverso da pandemia de COVID-19 no uso de serviços de saúde entre pacientes com diabetes tipo 2 na Carélia do Norte, Finlândia	10.1186/s12913-022-08105-z	Não aborda a temática
	Experiências de enfermeiros de saúde primários australianos no uso de telessaúde durante o COVID-19: um estudo qualitativo	https://pubmed.ncbi.nlm.nih.gov/34362804/	Estudo qualitativo
	Implementando a triagem remota em grandes sistemas de saúde: uma síntese de evidências qualitativas	https://pubmed.ncbi.nlm.nih.gov/33319411/	Não aborda a temática
	Percepção dos profissionais de enfermagem sobre a autoridade temporária de prática plena durante um surto de COVID-19: um estudo qualitativo	10.1016/j.ijnurstu.2021.104141	Não aborda a temática
	Desafios para unidades de asma em resposta ao COVID-19: uma análise qualitativa de dinâmica de grupo	10.1080/02770903.2021.1917605	Não aborda a temática
	Mudanças na prática clínica no monitoramento da hipertensão no início da pandemia de COVID-19	10.1093/ajh/hpac049	Não aborda a temática
	O que a prática geral pode aprender com as experiências dos enfermeiros de cuidados primários e dos assistentes de saúde da pandemia de COVID-19? Um estudo qualitativo	https://bmjopen.bmj.com/content/12/3/e055955	Estudo qualitativo
	Implementação de visitas de vídeo durante o COVID-19: lições aprendidas de uma prática de atenção primária na cidade de Nova York	10.3389/fpubh.2020.00514	Não aborda a temática

	Impacto percebido do COVID-19 na atenção primária na Nova Inglaterra: um estudo qualitativo	10.3122/jabfm.2022.02.210317	Não aborda a temática
	Profissionais de enfermagem em diabetes à sombra da pandemia de COVID-19: desafios, insights e sugestões para melhoria	10.1111/jnu.12754	Não aborda a temática
	Percepções dos profissionais de saúde sobre qualidade, aceitação e satisfação com os serviços de saúde telecomportamentais durante a pandemia de COVID-19: estudo baseado em pesquisa	10.2196/23245	Não aborda a temática
	Programa de Simulação de Comunicação Estruturada Telefônica para Acompanhamento de Casos e Contatos de COVID-19 na Atenção Básica	10.3390/ijerph19073915	Não aborda a temática
	Ações e adaptações implementadas para a prestação de serviços de saúde materna, neonatal e infantil durante a fase inicial da pandemia de COVID-19 em Lagos, Nigéria: estudo qualitativo dos líderes das unidades de saúde	10.5334/aogh.3529	Não aborda a temática
	Reorganização dos serviços de atenção primária durante a covid-19 no cabo ocidental, áfrica do sul: perspectivas dos enfermeiros da atenção primária	10.4102/safp.v63i1.5358	Não aborda a temática
	Uma telemedicina de dois gumes para cuidados maternos durante o COVID-19: resultados de um estudo global de métodos mistos de profissionais de saúde	10.1136/bmjgh-2020-004575	Não aborda a temática
	Experiência do paciente de telemedicina para tratamento de dor de cabeça durante a pandemia de COVID-19: um estudo de pesquisa da American Migraine Foundation	https://search.ebscohost.com/login.aspx?direct=true&db=c8h&AN=150790359&	Estudo quantitativo
	Fatores associados à implementação bem-sucedida do aborto por telessaúde em 4 ambientes de prática clínica dos Estados Unidos	https://www.embase.com/search/results?subaction=viewrecord&id=L2011977276&from=export	Não aborda a temática

EMBASE	Como a telessaúde atende às necessidades de idosos vulneráveis	10.3122/jabfm.2022.03.210493	Não aborda a temática
	Gestão no cuidado às pessoas com HIV na atenção primária à saúde em tempos do novo coronavírus	https://www.revistas.usp.br/rsp/article/view/196465	Estudo qualitativo
	Mudanças nas práticas de telessaúde na atenção primária em New Brunswick (Canadá): um estudo comparativo pré e durante a pandemia de COVID-19	10.1371/jornal.pone.0258839	Duplicado
	Impacto do COVID-19 no acesso dos migrantes aos cuidados primários e implicações para a implementação de vacinas: um estudo qualitativo nacional	10.3399/BJGP.2021.0028	Não aborda a temática
	Experiências de gerenciamento de enfermeiras precoces dos hubs COVID-19 finlandeses: uma revisão em ação	10.3390/ijerph19084885	Não aborda a temática
	A experiência dos enfermeiros na utilização da videoconsulta num ambiente de cuidados digitais e o seu impacto no seu fluxo de trabalho e comunicação	10.1371/jornal.pone.0264876	Não aborda a temática
	Mudanças nas apresentações com características potencialmente indicativas de câncer na atenção primária durante a pandemia de COVID-19: um estudo de coorte retrospectivo	10.1136/bmjopen-2021-050131	Não aborda a temática
	Experiências de pacientes com doença pulmonar obstrutiva crônica que recebem serviços integrados de enfermagem de telessaúde durante o bloqueio do COVID-19	10.1186/s12912-022-00967-2	Não aborda a temática

PUBMED	Avaliação preliminar de uma abordagem de telessaúde para avaliar, tratar e dar alta a pacientes de baixa acuidade com suspeita de COVID-19	10.1016/j.jemermed.2020.08.007	Não aborda a temática
	"Em casa, com cuidado": lições das práticas de cuidados primários domiciliares da cidade de Nova York Gerenciando o COVID-19	https://search.ebscohost.com/login.aspx?direct=true&db=c8h&AN=148453896&	Estudo qualitativo
	Implementação de consultoria remota na atenção primária do Reino Unido após a pandemia de COVID-19: um estudo longitudinal de métodos mistos	https://pubmed.ncbi.nlm.nih.gov/33558332/	Estudo de métodos mistos
	Efeito de uma intervenção abrangente de telessaúde versus telemonitoramento e coordenação de cuidados em pacientes com controle persistentemente ruim do diabetes tipo 2: um ensaio clínico randomizado	10.1001/jamainternmed.2022.2947	Não aborda a temática
	Telemonitoramento de enfermeiros navegadores para pacientes com câncer com COVID-19: um estudo de caso francês	10.1007/s00520-020-05968-y	Não aborda a temática
	Não está sozinho em casa: aproveitando a telessaúde e a informática para criar um modelo enxuto para o atendimento domiciliar de pacientes com COVID-19	10.1089/tmr.2021.0020	Não aborda a temática
	Oftalmologia remota com adaptador de smartphone manipulado por enfermeiros para diagnóstico de patologias do pólo posterior do olho durante a pandemia de COVID-19	10.1177/1357633x21994017	Não aborda a temática
	O impacto do COVID-19 nos cuidados crônicos de acordo com os provedores: um estudo qualitativo entre as práticas de atenção primária na Bélgica	https://search.ebscohost.com/login.aspx?direct=true&db=c8h&AN=147410280&	Estudo qualitativo

	Cuidados de saúde virtuais para gerenciamento comunitário de pacientes com COVID-19 na Austrália: estudo de coorte observacional	10.2196/21064	Não aborda a temática
	Explorando o uso e os desafios da implementação de visitas virtuais durante a COVID-19 na atenção primária e lições para uso sustentado	10.1371/jornal.pone.0253665	Não aborda a temática
	Enfrentando os Desafios da COVID-19: Avaliação das Mudanças Lideradas por Enfermeiros na Avaliação Telefônica	10.1097/nhh.0000000000001081	Duplicado
ESCOPUS	Experiências de enfermeiras de clínica geral de mudar a prestação de cuidados durante o COVID-19. Implicações para a prática futura: protocolo de estudo qualitativo	10.1111/jan.15312	Não aborda a temática
	Intervenções de enfermagem aumentam como medidas de qualidade da prevenção contra influenza para pacientes de telessaúde domiciliar	10.1097/NCQ.0000000000000577	Duplicado
	Usando a telessaúde para prestar cuidados primários a adolescentes durante e após a pandemia de Covid-19: resultados de uma pesquisa nacional com profissionais de cuidados primários	https://search.ebscohost.com/login.aspx?direct=true&db=c8h&AN=152822540&	Estudo quantitativo
	Intervenções de enfermagem aumentam as medidas de qualidade da vacinação contra a gripe para pacientes de telessaúde domiciliar	https://search.ebscohost.com/login.aspx?direct=true&db=c8h&AN=153704417&	Estudo quantitativo
	Mudanças nas práticas de telessaúde na atenção primária em New Brunswick (Canadá): um estudo comparativo pré e durante a pandemia de COVID-19	https://journals.plos.org/plosone/article?id=10.1371/journal.pone.0258839	Estudo quantitativo
	Implementação de serviços de monitoramento digital durante a pandemia de COVID-19 para pacientes com doenças crônicas: abordagem da ciência do design	https://pubmed.ncbi.nlm.nih.gov/34313591/	Estudo qualitativo

LILACS (0)	NENHUMA PUBLICAÇÃO		

129 ARTIGOS ENCONTRADOS NA BASE DE DADOS PUBMED

Nº	TÍTULO DO ARTIGO	LINK DE ACESSO/DOI
1	Pandemia de COVID-19 e acompanhamento uro-oncológico: Uma estratégia de equipe multidisciplinar "virtual" e avaliação da satisfação dos pacientes	10.4081/aiua.2020.2.78
2	Telemedicina e vacinação COVID-19 em uma clínica de medicina de HIV da Rede de Segurança Urbana	10.1016/j.nurpra.2022.06.005
3	Prescrição antecipada em cuidados comunitários de fim de vida no Reino Unido e na Irlanda durante a pandemia de COVID-19: pesquisa online	10.1136/bmjspcare-2020-002394
4	Uma plataforma de base tecnológica para avaliação de risco, detecção e prevenção de quedas entre idosos domiciliares: protocolo para um estudo quase experimental	10.2196/25781
5	O papel dos cuidados primários durante a pandemia de COVID-19: uma visão geral europeia	10.1016/j.aprim.2021.102134
6	Experiências de pacientes com doença pulmonar obstrutiva crônica que recebem serviços integrados de enfermagem de telessaúde durante o bloqueio do COVID-19	10.1186/s12912-022-00967-2
7	Avaliação da disponibilidade de saúde sexual e reprodutiva para mulheres migrantes venezuelanas durante a pandemia de SARS-CoV-2 na fronteira noroeste do Brasil-Venezuela	10.1016/j.jmh.2022.100092
8	Testes de COVID-19 e determinantes sociais da saúde entre bairros desfavorecidos de Baltimore: um modelo de alcance de clínica de saúde móvel comunitária	10.1089/pop.2021.0066
9	Gerenciamento de casos na linha de frente do COVID-19: a importância do plano de atendimento individualizado em todos os ambientes de atendimento	10.1097/ncm.0000000000000484
10	Taxas de mortalidade e readmissão entre pacientes com COVID-19 após alta do ambiente de cuidados agudos com oxigênio suplementar	10.1001/jamannetworkopen.2021.3990
11	Treinamento de resolução de problemas para veteranos com comorbidades complexas: adaptações de entrega de tratamento durante o COVID-19	10.1080/07317115.2021.1963382
12	Resultados da doença de coronavírus 2019 em lares de idosos franceses que implementaram o confinamento de funcionários com residentes	10.1001/jamannetworkopen.2020.17533
13	Infecção por SARS-CoV-2 em ambientes ocupacionais na Catalunha	Não disponível
14	Enfermeiros Psiquiátricos como Líderes na Integração da Saúde Comportamental	10.1016/j.nurpra.2020.09.001

15	Gestão da população de cuidados primários para pacientes com COVID-19	10.1007/s11606-020-05981-1
16	Cuidados de fim de vida durante a COVID-19: oportunidades e desafios para a enfermagem comunitária	10.12968/bjcn.2021.26.1.44
17	Telessaúde Interdisciplinar Centrada na Família e na Pessoa: Implicações de Políticas e Práticas Após o Início da Pandemia de COVID-19	10.3928/00989134-20200811-03
18	Telessaúde e a pandemia de COVID-19	10.1016/j.jopan.2020.06.023
19	Implementando melhores práticas de controle de infecção e qualidade de vida em lares de idosos com o projeto ECHO: protocolo para um estudo controlado randomizado centrado no paciente	10.2196/34480
20	Experiências de cirurgiões veterinários, enfermeiros veterinários e proprietários de consultas de telemedicina felina durante a pandemia de COVID-19 de 2020	10.1002/vetr.1738
21	Avaliação preliminar de uma abordagem de telessaúde para avaliar, tratar e dar alta a pacientes de baixa acuidade com suspeita de COVID-19	10.1016/j.jemermed.2020.08.007
22	Gestão no cuidado às pessoas com HIV na atenção primária à saúde em tempos do novo coronavírus	10.11606/s1518-8787.2022056003876
23	Aplicando a estrutura RE-AIM para avaliar um programa holístico de hospital para casa centrado no cuidador: um estudo de viabilidade sobre Carer Matters	10.1186/s12913-022-08317-3
24	Lançamento de um serviço de telemedicina e linha direta de atendimento primário COVID-19 em todo o estado	10.3122/jabfm.2021.S1.200178
25	Experiência do paciente de telemedicina para tratamento de dor de cabeça durante a pandemia de COVID-19: um estudo de pesquisa da American Migraine Foundation	10.1111/cabeça.14110
26	Explorando os efeitos da pandemia de COVID-19 nas crianças e famílias atendidas por enfermeiras registradas de prática avançada com foco em pediatria	10.1016/j.pedhc.2022.01.003
27	Efeito de uma intervenção abrangente de telessaúde versus telemonitoramento e coordenação de cuidados em pacientes com controle persistentemente ruim do diabetes tipo 2: um ensaio clínico randomizado	10.1001/jamainternmed.2022.2947
28	Utilização de telessaúde em instituições residenciais de cuidados a idosos durante a pandemia de COVID-19: um estudo de coorte retrospectivo na prática geral australiana	10.1177/1357633x221094406

29	O impacto do COVID-19 nos cuidados crônicos de acordo com os provedores: um estudo qualitativo entre as práticas de atenção primária na Bélgica	10.1186/s12875-020-01326-3
30	Desenvolvendo uma compreensão do conhecimento, atitudes e práticas dos adolescentes em relação ao COVID-19	10.1177/1059840520957069
31	Protocolo de pesquisa para explorar o uso de vídeo e telefone na clínica geral de Aotearoa Nova Zelândia: considerações para a futura telessaúde	10.1136/bmjhci-2020-100309
32	Avaliações econômicas de consultas por videoconferência e telefone na atenção primária: uma revisão sistemática	10.1177/1357633x211043380
33	Expansão da telessaúde na atenção primária durante a pandemia de COVID-19: benefícios e barreiras	10.1097/jxx.0000000000000626
34	Monitoramento domiciliar reduziu internações de curta duração em pacientes suspeitos de COVID-19: projeto COVID-box	10.1183/13993003.00636-2021
35	Telemedicina Acadêmica Direta ao Consumidor	10.1089/tmr.2022.0001
36	Telemonitoramento de enfermeiros navegadores para pacientes com câncer com COVID-19: um estudo de caso francês	10.1007/s00520-020-05968-y
37	Gerenciamento de cuidados primários de exacerbações ou ataques de asma: impacto da pandemia de COVID-19	10.1007/s12325-022-02056-x
38	O impacto do COVID-19 grave na qualidade de vida e incapacidade relacionadas à saúde: uma perspectiva de acompanhamento precoce	10.5935/0103-507X.20220008-pt
39	Não está sozinho em casa: aproveitando a telessaúde e a informática para criar um modelo enxuto para o atendimento domiciliar de pacientes com COVID-19	10.1089/tmr.2021.0020
40	"Em casa, com cuidado": lições das práticas de cuidados primários domiciliares de Nova York Gerenciando o COVID-19	10.1111/jgs.16952
41	"Há algo muito pessoal em ver o rosto de alguém": percepções do provedor de visitas de vídeo na atenção primária domiciliar durante o COVID-19	10.1177/07334648211028393
42	Avaliação da comunicação de estudantes de enfermagem na teleconsulta simulada: um estudo transversal	10.1016/j.nedt.2022.105382

43	Utilidade e aceitação do monitoramento telefônico por um assistente virtual para pacientes com COVID-19 após a alta	10.1016/j.rceng.2021.01.007
44	Tirando o 'cuidado' das casas de repouso: o dilema moral da prestação de cuidados institucionais de longo prazo durante o COVID-19	10.1111/hsc.13651
45	Usando a telessaúde para prestar cuidados primários a adolescentes durante e após a pandemia de COVID-19: estudo de pesquisa nacional de profissionais de cuidados primários dos EUA	10.2196/31240
46	Oftalmologia remota com adaptador de smartphone manipulado por enfermeiros para diagnóstico de patologias do pólo posterior do olho durante a pandemia de COVID-19	10.1177/1357633x21994017
47	Cinco etapas para integrar a telessaúde nos currículos da APRN	10.1016/j.nurpra.2020.12.004
48	Trabalhar em uma casa de repouso durante a pandemia de COVID-19: como a pandemia mudou as práticas de trabalho? Um estudo qualitativo	10.1186/s12877-022-02822-0
49	Resposta rápida centrada em telessaúde aos surtos de COVID-19 em instalações de cuidados pós-agudos e de longo prazo	10.1089/tmj.2020.0236
50	Integração de visitas médicas virtuais em um serviço telefônico provincial de informações de saúde 8-1-1 durante a pandemia de COVID-19: um estudo descritivo do HealthLink BC Emergency iDoctor-in-assistance (HEiDi)	10.9778/cmajo.20200265
51	Lições aprendidas (resultados clínicos e economia de custos) da Virtual Stone Clinic e sua aplicação na era pós-COVID-19: resultados prospectivos em um período de 6 anos de um hospital universitário	10.1089/final.2020.0708
52	Iniciando a medicação para transtorno do uso de opióides via telemedicina durante o COVID-19: implicações para as reformas propostas para a Lei Ryan Haight	10.1007/s11606-021-07174-w
53	Cuidados de saúde virtuais para gerenciamento comunitário de pacientes com COVID-19 na Austrália: estudo de coorte observacional	10.2196/21064
54	O efeito adverso da pandemia de COVID-19 no uso de serviços de saúde entre pacientes com diabetes tipo 2 na Carélia do Norte, Finlândia	10.1186/s12913-022-08105-z
55	Experiências de enfermeiros de saúde primários australianos no uso de telessaúde durante o COVID-19: um estudo qualitativo	10.1136/bmjopen-2021-049095

56	Melhorando as habilidades de humanização por meio de computadores baseados em simulação usando consultas de vídeo de enfermagem simuladas	10.3390/saúde 10010037
57	Mudanças nas práticas de telessaúde na atenção primária em New Brunswick (Canadá): um estudo comparativo pré e durante a pandemia de COVID-19	10.1371/jornal.pone.0258839
58	Educar os profissionais de saúde sobre a COVID-19 com a telementoring ECHO	10.1016/j.ajic.2021.10.030
59	Protocolo de um estudo multifásico sobre telemedicina para idosos na atenção primária	10.1136/bmjopen-2021-057061
60	Reengenharia do processo de transição de alta de pacientes com COVID-19 usando telemedicina, monitoramento remoto de pacientes e monitoramento remoto ininterrupto de pacientes do departamento de emergência e unidades de internação	10.1089/tmj.2020.0459
61	Percepções do estudante de enfermagem de uma visita piloto de telessaúde gerontológica simulada	10.3928/01484834-20210616-10
62	Implementação de serviços de monitoramento digital durante a pandemia de COVID-19 para pacientes com doenças crônicas: abordagem da ciência do design	10.2196/24181
63	Facilitando o implante de válvula aórtica transcateter na era do COVID-19: Recomendações para programas	10.1177/1474515120934057
64	Implementando a triagem remota em grandes sistemas de saúde: uma síntese de evidências qualitativas	10.1002/nr.22093
65	A informática clínica acelera a adaptação do sistema de saúde à pandemia de COVID-19: exemplos do Colorado	10.1093/jamia/ocaa171
66	Expansão dos serviços de farmácia durante o COVID-19: farmacêuticos e extensores de farmácias preenchendo as lacunas por meio de serviços de telessaúde	10.1177/00185787211032360
67	Caracterização e identificação de variações nos tipos de consultas de atenção primária antes e durante a pandemia de COVID-19 na Catalunha: estudo de análise de big data	10.2196/29622
68	Reimaginando a Enfermagem Escolar: Lições Aprendidas de uma Enfermeira Escolar Virtual	10.1177/1942602x21996432
69	Desafios para unidades de asma em resposta ao COVID-19: uma análise qualitativa de dinâmica de grupo	10.1080/02770903.2021.1917605

70	Precisão do teste de diagnóstico da avaliação de telessaúde para demência e comprometimento cognitivo leve	10.1002/14651858.CD013786.pub2
71	Pós-alta após a cirurgia Virtual Care with Remote Automated Monitoring Technology (PVC-RAM): protocolo para um estudo controlado randomizado	10.9778/cmajo.20200176
72	Pós-alta após a cirurgia Virtual Care com tecnologia Remote Automated Monitoring-1 (PVC-RAM-1) versus tratamento padrão: estudo controlado randomizado	10.1136/bmj.n2209
73	Geriatria de ligação com lares de idosos em tempo de COVID. Um novo modelo de coordenação chegou para ficar	10.1016/j.regg.2021.01.002
74	Explorando o uso e os desafios da implementação de visitas virtuais durante a COVID-19 na atenção primária e lições para uso sustentado	10.1371/jornal.pone.0253665
75	Implementação de consultoria remota na atenção primária do Reino Unido após a pandemia de COVID-19: um estudo longitudinal de métodos mistos	10.3399/bjgp.2020.0948
76	Experiências de gerenciamento de enfermeiras precoces dos hubs COVID-19 finlandeses: uma revisão em ação	10.3390/ijerph19084885
77	Desafios na prestação de cuidados de reumatologia durante a pandemia de COVID-19	10.1007/s10067-020-05312-z
78	O treinamento de habilidades de enfrentamento da dor desbloqueia o cuidado da dor centrado no paciente durante o bloqueio do COVID-19	10.1016/j.pmn.2021.10.007
79	Videoconsultas aumentadas em lares de idosos durante a pandemia de covid-19	10.3399/bjgpo.2022.0073
80	Saúde mental dos profissionais de enfermagem durante a pandemia de COVID-19: um estudo transversal	10.11606/s1518-8787.2022056004122
81	Evidências e Recomendações sobre o Uso da Telemedicina para o Tratamento da Hipertensão Arterial: Um Documento de Posição de Especialista Internacional	10,1161/hipertensãoaha.120,15873
82	Um estudo de caso de dois líderes de enfermagem especializada em saúde virtual durante a pandemia global do COVID-19	10.12927/cjnl.2021.26457
83	Usando um sistema de localização em tempo real para avaliar o impacto da telemedicina em um departamento de emergência durante o COVID-19: estudo observacional	10.2196/29240
84	Construindo um programa de segurança do paciente de monitoramento remoto em tempo real para pacientes com COVID-19	10.1097/jmq.0000000000000046
85	Intervenções de enfermagem aumentam as medidas de qualidade da vacinação contra a gripe para pacientes de telessaúde domiciliar	10.1097/ncq.0000000000000577

86	Alta hospitalar precoce após ICP para pacientes com STEMI	10.1016/j.jacc.2021.09.1379
87	Experiências dos pacientes de "longo COVID" na comunidade e recomendações para melhorar os serviços: uma pesquisa de melhoria da qualidade	10.1177/21501327211041846
88	A experiência dos enfermeiros na utilização da videoconsulta num ambiente de cuidados digitais e o seu impacto no seu fluxo de trabalho e comunicação	10.1371/jornal.pone.0264876
89	Mudanças na prática clínica no monitoramento da hipertensão no início da pandemia de COVID-19	10.1093/ajh/hpac049
90	Raça, etnia e resultados de 60 dias após hospitalização com COVID-19	10.1016/j.jamda.2021.08.023
91	Transformando os papéis interprofissionais durante a assistência à saúde virtual: o papel em evolução do assistente médico, em relação aos padrões nacionais de competência da profissão de saúde	10.1177/21501327211004285
92	Como as parteiras implementaram as teleconsultas durante a crise de saúde do COVID-19: um estudo de métodos mistos	10.1136/bmjopen-2021-057292
93	O que a prática geral pode aprender com as experiências dos enfermeiros de cuidados primários e dos assistentes de saúde da pandemia de COVID-19? Um estudo qualitativo	10.1136/bmjopen-2021-055955
94	Quando as entrevistas presenciais não são possíveis: dicas e truques para entrevistas por vídeo, telefone, chat online e e-mail na pesquisa qualitativa	10.1093/eurjcn/zvab038
95	Cuidando de mulheres com transtornos por uso de substâncias durante a gravidez e pós-parto durante a pandemia de COVID-19: Lições aprendidas com estagiários de psicologia em um programa de tratamento ambulatorial integrado de ginecologia/uso de substâncias	10.1016/j.jsat.2020.108200
96	Determinantes da intenção dos profissionais da atenção primária pública catalã em usar consultas clínicas digitais (eConsulta) no contexto pós-COVID-19: ilusão de ótica ou transformação permanente?	10.2196/28944
97	Análise das Consequências da Pandemia de COVID-19 em Pessoas com Transtornos Mentais Graves	10.3390/ijerph18168549
98	Satisfação do paciente com a consulta telefônica de fim de tratamento liderada por enfermeira para câncer de mama durante a pandemia de COVID-19	10.1111/tbj.14034
99	O impacto da pandemia de COVID nos serviços de atenção primária ao diabetes no Reino Unido: uma pesquisa nacional transversal de pontos de vista dos profissionais de saúde que prestam cuidados ao diabetes	10.1016/j.pcd.2021.12.015

100	Efeito de uma intervenção baseada em dispositivos móveis na saúde mental em profissionais de saúde da linha de frente contra o COVID-19: Protocolo para um estudo controlado randomizado	10.1111/jan.14813
101	Assistência Farmacêutica em Pacientes Hospitalizados. (Gestão da crise pandémica COVID-19. Um novo desafio para os serviços farmacêuticos)	10.7399/fh.11513
102	Alavancando equipes interdisciplinares para planejamento pré-visita para melhorar as taxas de imunização pneumocócica entre práticas de subespecialidade de medicina interna	10.1177/21501319211060986
103	Um novo programa integrado de telemonitoramento em larga escala para COVID-19	10.1089/tmj.2020.0384
104	Parceria da Feira de Telessaúde COVID-19 para Capacitação em Enfermagem de Atenção Primária	10.1097/naq.0000000000000517
105	Integração vertical das práticas de GP com hospitais de agudos na Inglaterra e no País de Gales: avaliação rápida	10.3310/tlla3317
106	Implementação do telemonitoramento COVID-19: repercussões na formação acadêmica de Enfermagem.	10.1590/1983-1447.2021.20200395
107	Implementação de visitas de vídeo durante o COVID-19: lições aprendidas de uma prática de atenção primária na cidade de Nova York	10.3389/fpubh.2020.00514
108	Gerenciamento desafiador de distúrbios crônicos graves em situação de pandemia aguda: doença hepática crônica sob pandemia de COVID-19 como modelo de prova de princípio para orquestrar as medidas no contexto das 15h	10.1007/s13167-021-00231-8
109	Cuidados paliativos especializados em hospitais agudos: modelos de equipe multidisciplinar e força de trabalho no sudoeste da Inglaterra	10.1136/spcare-2022-003813
110	Experiências dos profissionais da atenção primária durante a primeira onda da pandemia de COVID-19 na Grécia: um estudo qualitativo	10.1186/s12875-021-01522-9
111	O impacto da pandemia de COVID-19 no futuro da telessaúde na atenção primária	10.1016/j.outlook.2021.09.004
112	Enfrentando os Desafios da COVID-19: Avaliação das Mudanças Lideradas por Enfermeiros na Avaliação Telefônica	10.1097/nhh.0000000000001081
113	Avaliação qualitativa da transformação rápida do sistema para visitas de vídeo de atenção primária em um centro médico acadêmico	10,7326/m20-1814

114	Desenvolvimento da ferramenta de estimativa de poupança e utilização de cuidados de longa duração de consulta eletrónica para modelar o impacto potencial da consulta eletrónica para residentes que vivem em cuidados de longa duração	10.1177/1357633x221074500
115	Envolvimento do farmacêutico em um programa abrangente de monitoramento remoto e telegestão	10.1093/ajhp/zxac025
116	Intervenções baseadas em tecnologia para residentes de asilos: um protocolo de revisão sistemática	10.1136/bmjopen-2021-056142
117	Impacto percebido do COVID-19 na atenção primária na Nova Inglaterra: um estudo qualitativo	10.3122/jabfm.2022.02.210317
118	Características das consultas telefônicas gratuitas relacionadas à COVID-19 por enfermeiras de saúde pública no Japão: um estudo retrospectivo	10.3390/saúde 9081022
119	O impacto da pandemia de COVID-19 na prestação de cuidados e qualidade de vida em cirurgia de câncer de pulmão	10.1002/jso.26902
120	Caindo nas rachaduras: avaliando o papel do pessoal de ligação cirúrgica não aguda durante a revisão narrativa do COVID-19-A	10.1016/j.surg.2021.08.040
121	Cuidados percebidos e bem-estar de pacientes com câncer e participantes de normas compatíveis na crise do COVID-19: resultados de uma pesquisa com participantes no registro holandês de perfis	10.1001/jamaoncol.2020.6093
122	Monitoramento remoto em pacientes com insuficiência cardíaca crônica: o monitoramento remoto não invasivo é o caminho a percorrer?	10.3390/s21030887
123	Mudança no fluxo de trabalho de enfermagem em uma unidade de internação COVID-19 após a implantação da telessaúde hospitalar: estudo observacional usando um sistema de localização em tempo real	10.2196/36882
124	Perspectivas do paciente da telemedicina hospitalar durante a pandemia de COVID-19: avaliação qualitativa	10.2196/32933
125	Aproveitando a Telemedicina para a Prestação de Cuidados de Saúde: Análise Bibliométrica e Cienciométrica	10.2196/18835
126	Implicações da política de telessaúde para enfermeiras registradas na atenção primária	10.1177/1357633x20940142
127	Percepções dos profissionais de saúde sobre qualidade, aceitação e satisfação com os serviços de saúde telecomportamentais durante a pandemia de COVID-19: estudo baseado em pesquisa	10.2196/23245

128	Condução bem-sucedida de um ensaio clínico de acidente vascular cerebral agudo durante o COVID	10.1371/jornal.pone.0243603
129	Relação entre alfabetização em eSaúde e estado psicológico durante a pandemia de COVID-19: uma pesquisa com residentes chineses	10.1111/jonm.13221

64 ARTIGOS ENCONTRADOS NA BASE DE DADOS CINAHL

Nº	TÍTULO DO ARTIGO	LINK DE ACESSO/DOI
1	Colaboração entre provedores para tratar pacientes com COVID-19 em casa abre leitos para pessoas com doenças mais graves	https://search.ebscohost.com/login.aspx?direct=true&db=c8h &AN=157053210&
2	Notícias Globais da AJPH	https://search.ebscohost.com/login.aspx?direct=true&db=c8h &AN=155395340&
3	Cuidados de fim de vida em uma pandemia: uma perspectiva da atenção primária	https://search.ebscohost.com/login.aspx?direct=true&db=c8h &AN=149688333&
4	Resultados da doença de coronavírus 2019 em lares de idosos franceses que implementaram o confinamento de funcionários com residentes	https://search.ebscohost.com/login.aspx?direct=true&db=c8h &AN=145136999&
5	Enfermeiros Psiquiátricos como Líderes na Integração da Saúde Comportamental	https://search.ebscohost.com/login.aspx?direct=true&db=c8h &AN=148185264&
6	Estágio curricular supervisionado em enfermagem na pandemia Covid-19: experiência no programa Brasil Conta Comigo	https://search.ebscohost.com/login.aspx?direct=true&db=c8h &AN=154327581&
**7**	Implementação do telemonitoramento COVID-19: repercussões na formação acadêmica de Enfermagem.	https://search.ebscohost.com/login.aspx?direct=true&db=c8h &AN=153772347&
8	COVID-19 e seu efeito na equipe de enfermagem na atenção primária	https://search.ebscohost.com/login.aspx?direct=true&db=c8h &AN=147312149&
9	O Papel da Telessaúde e da Informática Clínica na Redesenho da Atenção Primária Orientada por Dados	https://search.ebscohost.com/login.aspx?direct=true&db=c8h &AN=154522952&<br>lang=pt-br&site=ehost-live
10	Experiência do paciente de telemedicina para tratamento de dor de cabeça durante a pandemia de COVID-19: um estudo de pesquisa da American Migraine Foundation	https://search.ebscohost.com/login.aspx?direct=true&db=c8h &AN=150790359&
11	A consulta de enfermagem no enfrentamento da COVID-19: vivência na atenção primária à saúde	https://search.ebscohost.com/login.aspx?direct=true&db=c8h &AN=154835818&
12	Resultados de hospitalização entre pacientes com COVID-19 em monitoramento remoto	https://search.ebscohost.com/login.aspx?direct=true&db=c8h &AN=157890783&
13	O impacto do COVID-19 nos cuidados crônicos de acordo com os provedores: um estudo qualitativo entre as práticas de atenção primária na Bélgica	https://search.ebscohost.com/login.aspx?direct=true&db=c8h &AN=147410280&
14	Desenvolvendo uma compreensão do conhecimento, atitudes e práticas dos adolescentes em relação ao COVID-19	https://search.ebscohost.com/login.aspx?direct=true&db=c8h &AN=147065468&
15	Conhecimento e uso de equipamentos de proteção individual por profissionais de enfermagem durante pandemia da Covid-19	https://search.ebscohost.com/login.aspx?direct=true&db=c8h &AN=154327773&
16	Expansão da telessaúde na atenção primária durante a pandemia de COVID-19: benefícios e barreiras	https://search.ebscohost.com/login.aspx?direct=true&db=c8h &AN=155110078&
17	Manejo do cuidado e educação em saúde na atenção básica na pandemia do Coronavírus	https://search.ebscohost.com/login.aspx?direct=true&db=c8h &AN=145691194&
18	Um modelo expandido de cuidados intermediários de telemedicina COVID-19 usando quartos de hotel reaproveitados	https://search.ebscohost.com/login.aspx?direct=true&db=c8h &AN=146485147&
19	Impacto na atividade assistencial e na saúde dos profissionais de Atenção Primária durante o confinamento	https://search.ebscohost.com/login.aspx?direct=true&db=c8h &AN=152354073&
20	"Em casa, com cuidado": lições das práticas de cuidados primários domiciliares da cidade de Nova York Gerenciando o COVID-19	https://search.ebscohost.com/login.aspx?direct=true&db=c8h &AN=1484538896&
21	"Há algo muito pessoal em ver o rosto de alguém": percepções do provedor de visitas de vídeo na atenção primária domiciliar durante o COVID-19	https://search.ebscohost.com/login.aspx?direct=true&db=c8h &AN=153419779&
22	Considerações ao usar a telemedicina como enfermeira registrada de prática avançada	https://search.ebscohost.com/login.aspx?direct=true&db=c8h &AN=149243077&
23	Avaliação da comunicação de estudantes de enfermagem na teleconsulta simulada: um estudo	https://search.ebscohost.com/login.aspx?direct=true&db=c8h &AN=[illegible]

	transversal	&AN=156781830&
24	Usando a telessaúde para prestar cuidados primários a adolescentes durante e após a pandemia de Covid-19: resultados de uma pesquisa nacional com profissionais de cuidados primários	https://search.ebscohost.com/login.aspx?direct=true&db=c8h &AN=152822540&
25	Cinco etapas para integrar a telessaúde nos currículos da APRN	https://search.ebscohost.com/login.aspx?direct=true&db=c8h &AN=149243080&
26	Desenvolvimento de um serviço de teledermatologia na zona rural do estado de Washington usando o conjunto de habilidades de prática de enfermagem e ciência de implementação	https://search.ebscohost.com/login.aspx?direct=true&db=c8h &AN=152028128&
27	Consultas remotas de asma na atenção primária	https://search.ebscohost.com/login.aspx?direct=true&db=c8h &AN=148521996&
28	Educar os profissionais de saúde sobre a COVID-19 com a telementoring ECHO	https://search.ebscohost.com/login.aspx?direct=true&db=c8h &AN=155286039&
29	As necessidades dos sobreviventes de câncer em idosos durante o COVID-19: implicações para a enfermagem oncológica	https://search.ebscohost.com/login.aspx?direct=true&db=c8h &AN=153706823&amp
30	Lições aprendidas com a pandemia - podemos fazer melhor	https://search.ebscohost.com/login.aspx?direct=true&db=c8h &AN=157054809&amp
31	Percepções do estudante de enfermagem de uma visita piloto de telessaúde gerontológica simulada	https://search.ebscohost.com/login.aspx?direct=true&db=c8h &AN=151282222&amp
32	Cuidado do paciente na atenção primária: Contribuição da tecnologia durante uma pandemia	https://search.ebscohost.com/login.aspx?direct=true&db=c8h &AN=147249155&amp
33	Telessaúde para pacientes com diabetes	https://search.ebscohost.com/login.aspx?direct=true&db=c8h &AN=158121610&
34	Expansão dos serviços de farmácia durante o COVID-19: farmacêuticos e extensores de farmácias preenchendo as lacunas por meio de serviços de telessaúde	https://search.ebscohost.com/login.aspx?direct=true&db=c8h &AN=156993506&amp
35	O papel do enfermeiro no apoio a pacientes com asma durante a pandemia de COVID-19	https://search.ebscohost.com/login.aspx?direct=true&db=c8h &AN=148673478&
36	Tecnologia móvel para o cuidado de enfermagem durante a pandemia da covid-19	https://search.ebscohost.com/login.aspx?direct=true&db=c8h &AN=148440428&
37	O papel e a resposta dos serviços de saúde primários na prestação de cuidados paliativos em epidemias e pandemias: uma revisão rápida para informar a prática e a prestação de serviços durante a pandemia de COVID-19	https://search.ebscohost.com/login.aspx?direct=true&db=c8h &AN=145889977&
38	La video-consulta en atención primaria de salud: una experiencia de implantación	https://search.ebscohost.com/login.aspx?direct=true&db=c8h &AN=153606812&<br>lang=pt-br&site=ehost-live
39	Intervenções de enfermagem aumentam as medidas de qualidade da vacinação contra a gripe para pacientes de telessaúde domiciliar	https://search.ebscohost.com/login.aspx?direct=true&db=c8h &AN=153704417&
40	Experiências dos pacientes de "longo COVID" na comunidade e recomendações para melhorar os serviços: uma pesquisa de melhoria da qualidade	https://search.ebscohost.com/login.aspx?direct=true&db=c8h &AN=154384635&
41	Experiências dos pacientes de "longo COVID" na comunidade e recomendações para melhorar os serviços: uma pesquisa de melhoria da qualidade	https://search.ebscohost.com/login.aspx?direct=true&db=c8h &AN=152311401&
42	Teleenfermagem no serviço de atenção domiciliar na pandemia de COVID-19: um estudo transversal	https://search.ebscohost.com/login.aspx?direct=true&db=c8h &AN=153037967&
43	Transformando os papéis interprofissionais durante a assistência virtual à saúde: o papel em evolução do assistente médico, em relação aos padrões nacionais de competência da profissão de saúde	https://search.ebscohost.com/login.aspx?direct=true&db=c8h &AN=149494630&

44	Transformando os papéis interprofissionais durante a assistência virtual à saúde: o papel em evolução do assistente médico, em relação aos padrões nacionais de competência da profissão de saúde	https://search.ebscohost.com/login.aspx?direct=true&db=c8h&AN=149494630&
45	Determinantes da intenção dos profissionais da atenção primária pública catalã em usar consultas clínicas digitais (eConsulta) no contexto pós-COVID-19: ilusão de ótica ou transformação permanente?	https://search.ebscohost.com/login.aspx?direct=true&db=c8h&AN=151188870&
46	Conhecimento e uso de equipamentos de proteção individual por profissionais de enfermagem durante a pandemia de Covid-19	https://search.ebscohost.com/login.aspx?direct=true&db=c8h&AN=1543275545&
47	Ações de atenção primária em saúde para pessoas em situação de rua durante a covid-19: um relato de experiência	https://search.ebscohost.com/login.aspx?direct=true&db=c8h&AN=153589270&
48	Como realizar consultas remotas para pessoas com asma e DPOC: enfermeiros da atenção primária estão transformando suas formas de trabalho para garantir a segurança de pacientes e funcionários	https://search.ebscohost.com/login.aspx?direct=true&db=c8h&AN=1486734858&
49	Análise baseada em big data para caracterizar e identificar variações no tipo de consultas de Atenção Primária antes e durante a COVID na Catalunha	https://search.ebscohost.com/login.aspx?direct=true&db=c8h&AN=152822450&
50	O impacto da pandemia de COVID nos serviços de atenção primária ao diabetes no Reino Unido: uma pesquisa nacional transversal de pontos de vista dos profissionais de saúde que prestam cuidados ao diabetes	https://search.ebscohost.com/login.aspx?direct=true&db=c8h&AN=156764499&
51	Alavancando equipes interdisciplinares para planejamento pré-visita para melhorar as taxas de imunização pneumocócica entre práticas de subespecialidade de medicina interna	https://search.ebscohost.com/login.aspx?direct=true&db=c8h&AN=153991922&
52	Alavancando equipes interdisciplinares para planejamento pré-visita para melhorar as taxas de imunização pneumocócica entre práticas de subespecialidade de medicina interna	https://search.ebscohost.com/login.aspx?direct=true&db=c8h&AN=153991922&
53	Parceria da Feira de Telessaúde COVID-19 para Capacitação em Enfermagem de Atenção Primária	https://search.ebscohost.com/login.aspx?direct=true&db=c8h&AN=155621307&
54	Entendendo as redes de segurança em consultoria remota	https://search.ebscohost.com/login.aspx?direct=true&db=c8h&AN=148521999&
55	Experiências dos profissionais da atenção primária durante a primeira onda da pandemia de COVID-19 na Grécia: um estudo qualitativo	https://search.ebscohost.com/login.aspx?direct=true&db=c8h&AN=152227760&
56	O impacto da pandemia de COVID-19 no futuro da telessaúde na atenção primária	https://search.ebscohost.com/login.aspx?direct=true&db=c8h&AN=156225390&
57	Enfrentando os Desafios da COVID-19: Avaliação das Mudanças Lideradas por Enfermeiros na Avaliação Telefônica	https://search.ebscohost.com/login.aspx?direct=true&db=c8h&AN=157746222&<br>lang=pt-br&site=ehost-live
58	Avaliação qualitativa da transformação rápida do sistema para visitas de vídeo de atenção primária em um centro médico acadêmico	https://search.ebscohost.com/login.aspx?direct=true&db=c8h&AN=146561390&
59	Envolvimento do farmacêutico em um programa abrangente de monitoramento remoto e telegestão	https://search.ebscohost.com/login.aspx?direct=true&db=c8h&AN=157074085&
60	Acompanhamento do paciente com síndrome coronario agudo no caso de novo confinamento por COVID-19: aportación de la enfermera gestora de cuidados cardiológicos	https://search.ebscohost.com/login.aspx?direct=true&db=c8h&AN=151184430&
61	Aproveitando a Telemedicina para a Prestação de Cuidados de Saúde: Análise Bibliométrica e Cienciométrica	https://search.ebscohost.com/login.aspx?direct=true&db=c8h&AN=146783873&
62	Implicações da política de telessaúde para enfermeiras registradas na atenção primária	https://search.ebscohost.com/login.aspx?direct=true&db=c8h&AN=156121323&

63	Um novo programa de cuidados colaborativos para aumentar os cuidados domiciliares de enfermagem durante e após a pandemia de COVID-19	https://search.ebscohost.com/login.aspx?direct=true&db=c8h&AN=154895165&
64	Relação entre a alfabetização em eSaúde e o estado psicológico durante a pandemia de COVID-19: uma pesquisa com residentes chineses	https://search.ebscohost.com/login.aspx?direct=true&db=c8h&AN=150340194&

14 ARTIGOS ENCONTRADOS NA BASE DE DADOS LILACS

Nº	TÍTULO DO ARTIGO	LINK DE ACESSO/DOI
1	Diretrizes brasileiras e portuguesas de proteção à criança vulnerável à violência na pandemia de COVID-19	https://www.scielo.br/j/ean/a/3sbrMF4HvD4V7BvRVmsWyVf
2	Vulnerabilidades da saúde infantil durante a pandemia de COVID-19 no Brasil e em Portugal	http://www.revenf.bvs.br/scielo.php?script=sci_arttext&pid=S0104-11692021000100330
3	Análise de conteúdo do fórum de discussão da Aliança Global para Enfermagem e Obstetrícia: uma comunidade de prática online	http://www.scielo.br/scielo.php?script=sci_arttext&pid=S0104-11692021000100371
4	A consulta de enfermagem no enfrentamento da COVID-19: vivência na atenção primária à saúde	https://revistas.ufg.br/fen/article/view/65893
5	Reorganização do serviço ambulatorial de referência para condições crônicas durante a pandemia da COVID-19	http://www.revenf.bvs.br/scielo.php?script=sci_arttext&pid=S1414-81452022000100602
6	Monitoramento telefônico de dois casos de infecção pelo novo Coronavírus: relato de experiência	https://periodicos.ufpel.edu.br/ojs2/index.php/enfermagem/article/view/19946/12224
7	Conhecimento e uso de equipamentos de proteção individual por profissionais de enfermagem durante a pandemia de Covid-19	http://www.scielo.br/scielo.php?script=sci_arttext&pid=S0080-62342021000100573
8	Estratégias para continuidade a das imunizações durante a COVID-19 em Tucuruí, PA	http://revistas.mpmcomunicacao.com.br/index.php/revistanursing/article/view/1117/1323
9	Atenção Primária à Saúde frente à COVID-19: Relato de experiência de um Centro de Saúde	http://revista.cofen.gov.br/index.php/enfermagem/article/view/3666/836
10	Teleconsulta no serviço de atenção domiciliar na pandemia da COVID-19: estudo transversal	https://fi-admin.bvsalud.org/document/view/bvvtw
11	Vacinação contra influenza no enfrentamento da COVID-19: integração ensino-serviço para formação em enfermagem e saúde	https://www.scielo.br/scielo.php?script=sci_arttext&pid=S1414-81452021000500601
12	Implementação do telemonitoramento COVID-19: repercussões na formação acadêmica de Enfermagem.	http://www.revenf.bvs.br/scielo.php?script=sci_arttext&pid=S1983-14472021000100809
13	Estágio supervisionado curricular em enfermagem durante a pandemia de coronavírus: experiências na atenção básica	https://periodicos.ufpel.edu.br/ojs2/index.php/enfermagem/article/view/19050
14	Influência da cobertura da atenção básica no enfrentamento da COVID-19	https://periodicos.unemat.br/index.php/jhnpeps/article/view/4994

15 ARTIGOS ENCONTRADOS NA BASE DE DADOS BDENF

Nº	TÍTULO DO ARTIGO	LINK DE ACESSO/DOI
1	Diretrizes brasileiras e portuguesas de proteção à criança vulnerável à violência na pandemia de COVID-19	https://www.scielo.br/j/ean/a/3sbrMF4HvD4V7BvRVmsWyVf
2	Vulnerabilidades da saúde infantil durante a pandemia de COVID-19 no Brasil e em Portugal	http://www.revenf.bvs.br/scielo.php?script=sci_arttext&pid=S0104-11692021000100330
3	Análise de conteúdo do fórum de discussão da Aliança Global para Enfermagem e Obstetrícia: uma comunidade de prática online	http://www.scielo.br/scielo.php?script=sci_arttext&pid=S0104-11692021000100371
4	A consulta de enfermagem no enfrentamento da COVID-19: vivência na atenção primária à saúde	https://revistas.ufg.br/fen/article/view/65893
5	Reorganização do serviço ambulatorial de referência para condições crônicas durante a pandemia da COVID-19	http://www.revenf.bvs.br/scielo.php?script=sci_arttext&pid=S1414-81452022000100602
6	Monitoramento telefônico de dois casos de infecção pelo novo Coronavírus: relato de experiência	https://periodicos.ufpel.edu.br/ojs2/index.php/enfermagem/article/view/19946/12224
7	Conhecimento e uso de equipamentos de proteção individual por profissionais de enfermagem durante a pandemia de Covid-19	http://www.scielo.br/scielo.php?script=sci_arttext&pid=S0080-62342021000100573
8	Estratégias para continuidade a das imunizações durante a COVID-19 em Tucuruí, PA	http://revistas.mpmcomunicacao.com.br/index.php/revistanursing/article/view/1117/1323
9	Atenção Primária à Saúde frente à COVID-19: Relato de experiência de um Centro de Saúde	http://revista.cofen.gov.br/index.php/enfermagem/article/view/3666/836
10	Teleconsulta no serviço de atenção domiciliar na pandemia da COVID-19: estudo transversal	https://fi-admin.bvsalud.org/document/view/bvvtw
11	Vacinação contra influenza no enfrentamento da COVID-19: integração ensino-serviço para formação em enfermagem e saúde	https://www.scielo.br/scielo.php?script=sci_arttext&pid=S1414-81452021000500601
12	Implementação do telemonitoramento COVID-19: repercussões na formação acadêmica de Enfermagem.	http://www.revenf.bvs.br/scielo.php?script=sci_arttext&pid=S1983-14472021000100809
13	Estágio supervisionado curricular em enfermagem durante a pandemia de coronavírus: experiências na atenção básica	https://periodicos.ufpel.edu.br/ojs2/index.php/enfermagem/article/view/19050

14	Influência da cobertura da atenção básica no enfrentamento da COVID-19	https://periodicos.unemat.br/index.php/jhnpeps/article/view/4994
15	Influência da cobertura da atenção básica no enfrentamento da COVID-19	https://periodicos.unemat.br/index.php/jhnpeps/article/view/4994

06 ARTIGOS ENCONTRADOS NA BASE DE DADOS SCIELO

Nº	TÍTULO DO ARTIGO	LINK DE ACESSO/DOI
1	Diretrizes brasileiras e portuguesas de proteção à criança vulnerável à violência na pandemia de COVID-19	http://www.scielo.br/scielo.php?script=sci_arttext&pid=S1414-81452021000500208&lang=pt
2	Vulnerabilidades da saúde infantil durante a pandemia de COVID-19 no Brasil e em Portugal	http://www.scielo.br/scielo.php?script=sci_arttext&pid=S0104-11692021000100330&lang=pt
3	Intervenção multiprofissional e teleenfermagem no tratamento de pessoas obesas na pandemia de COVID-19: um ensaio clínico pragmático	http://www.scielo.br/scielo.php?script=sci_arttext&pid=S0034-71672022001100214&lang=pt
4	Conhecimento e uso de equipamentos de proteção individual por profissionais de enfermagem durante a pandemia de Covid-19	http://www.scielo.br/scielo.php?script=sci_arttext&pid=S0080-62342021000100573&lang=pt
5	Relato de experiência das contribuições da telessaúde em comunidades ribeirinhas do Amazonas na pandemia	http://www.scielo.br/scielo.php?script=sci_arttext&pid=S0034-71672022001100501&lang=pt
6	Implementação do telemonitoramento COVID-19: repercussões na formação acadêmica de Enfermagem.	http://www.scielo.br/scielo.php?script=sci_arttext&pid=S1983-14472021000200809&lang=pt

105 ARTIGOS ENCONTRADOS NA BASE DE DADOS SCOPUS

Nº	TÍTULO DO ARTIGO	LINK DE ACESSO/DOI
1	Programa de Simulação de Comunicação Estruturada Telefônica para Acompanhamento de Casos e Contatos de COVID-19 na Atenção Básica	10.3390/ijerph19073915
2	Uma pesquisa transversal sobre práticas de gerenciamento de medicamentos para doenças não transmissíveis na Europa durante a segunda onda da pandemia de COVID-19	10.3389/ffar.2021.685696
3	Avaliações interativas de saúde mental para chineses canadenses: um estudo piloto randomizado controlado em uma clínica de cuidados primários liderada por enfermeiros	10.1111/app.12400
4	Perspectivas de médicos de cuidados primários em Massachusetts sobre o uso de telemedicina com adultos com 65 anos ou mais durante a pandemia de COVID-19	10.1016/j.pmedr.2022.101729
5	Experiências de enfermeiras de clínica geral de mudar a prestação de cuidados durante o COVID-19. Implicações para a prática futura: protocolo de estudo qualitativo	10.1111/jan.15312
6	Telemedicina e vacinação COVID-19 em uma clínica de medicina de HIV da Rede de Segurança Urbana	10.1016/j.nurpra.2022.06.005
7	Uma plataforma de base tecnológica para avaliação de risco, detecção e prevenção de quedas entre idosos domiciliares: protocolo para um estudo quase experimental	10.2196/25781
8	O papel dos cuidados primários durante a pandemia de COVID-19: uma visão geral europeia	10.1016/j.aprim.2021.102134
9	Avaliação da disponibilidade de saúde sexual e reprodutiva para mulheres venezuelanas migrantes durante a pandemia de SARS-CoV- na fronteira noroeste do Brasil	10.1016/j.jmh.2022.100092
10	Testes de COVID-19 e determinantes sociais da saúde entre bairros desfavorecidos de Baltimore: um modelo comunitário de atendimento em clínica móvel de saúde	10.1089/pop.2021.0066
11	Gerenciamento de casos na linha de frente do COVID-19: a importância do plano de atendimento individualizado em todos os ambientes de atendimento	10.1097/NCM.0000000000000484
12	Ações e adaptações implementadas para a prestação de serviços de saúde materna, neonatal e infantil durante a fase inicial da pandemia de COVID-19 em Lagos, Nigéria: estudo qualitativo de líderes de unidades de saúde	10.5334/aogh.3529
13	Treinamento de resolução de problemas para veteranos com comorbidades complexas: adaptações de entrega de tratamento durante o COVID-19	10.1080/07317115.2021.1963382
14	Resultados da doença de coronavírus 2019 em lares de idosos franceses que implementaram o confinamento de funcionários com residentes	10.1001/jamannetworkopen.2020.17533
15	Acesso a serviços de saúde mental de refugiados e migrantes durante os primeiros seis meses da pandemia de covid-19: uma pesquisa de médicos de refugiados canadenses	10.3390/ijerph18105266
16	Enfermeiros Psiquiátricos como Líderes na Integração da Saúde Comportamental	10.1016/j.nurpra.2020.09.001

17	Gestão da população de cuidados primários para pacientes com COVID-19	10.1007/s11606-020-05981-1
18	Vulnerabilidades da saúde infantil durante a pandemia de covid-19 no Brasil e em Portugal	10.1590/1518-8345.4805.3422
19	Experiências de cirurgiões veterinários, enfermeiros veterinários e proprietários de consultas de telemedicina felina durante a pandemia de COVID-19 de 2020	10.1002/vetr.1738
20	Gestão no cuidado às pessoas com HIV na atenção primária à saúde em tempos do novo coronavírus	10.11606/S1518-8787.2022056003876
21	Lançamento de uma linha direta de atendimento primário COVID-19 em todo o estado e serviço de telemedicina	10.3122/JABFM.2021.S1.200178
22	Experiência do paciente de telemedicina para tratamento de dor de cabeça durante a pandemia de COVID-19: um estudo de pesquisa da American Migraine Foundation	10.1111/cabeça.14110
23	Tendências de prescrição de antibióticos na atenção primária fora de horas belga durante a pandemia de covid-19: estudo observacional usando dados de saúde coletados rotineiramente	10.3390/antibióticos10121488
24	Reorganização dos serviços de atenção primária durante a covid-19 no cabo ocidental, áfrica do sul: perspectivas dos enfermeiros da atenção primária	10.4102/safp.v63i1.5358
25	Utilização de telessaúde em instituições residenciais de cuidados a idosos durante a pandemia de COVID-19: um estudo de coorte retrospectivo na prática geral australiana	10.1177/1357633X221094406
26	O impacto do COVID-19 nos cuidados crônicos de acordo com os provedores: um estudo qualitativo entre as práticas de atenção primária na Bélgica	10.1186/s12875-020-01326-3
27	Desenvolvendo uma compreensão do conhecimento, atitudes e práticas dos adolescentes em relação ao COVID-19	10.1177/1059840520957069
28	Protocolo de pesquisa para explorar o uso de vídeo e telefone na clínica geral de Aotearoa Nova Zelândia: Considerações para a futura telessaúde	10.1136/bmjhci-2020-100309
29	Um roteiro da Rede de Pesquisa Baseada na Prática (PBRN) para avaliar o COVID-19 em centros de saúde comunitários: um relatório do OCHIN PBRN	10.3122/JABFM.2020.05.200053
30	Expansão da telessaúde na atenção primária durante a pandemia de COVID-19: benefícios e barreiras	10.1097/JXX.0000000000000626
31	Persistência e determinantes basais das taxas de soropositividade e reinfecção em profissionais de saúde até 12,5 meses após o COVID-19	10.1186/s12916-021-02032-2
32	O impacto do confinamento COVID-19 na cognição e saúde mental e uso de tecnologia entre idosos socialmente vulneráveis: estudo de coorte retrospectivo	10.2196/30598

33	“Em casa, com cuidado”: lições das práticas de cuidados primários domiciliares da cidade de Nova York Gerenciando o COVID-19	10.1111/jgs.16952
34	“Há algo muito pessoal em ver o rosto de alguém”: percepções do provedor de visitas de vídeo na atenção primária domiciliar durante o COVID-19	10.1177/07334648211028393
35	Avaliação da comunicação de estudantes de enfermagem na teleconsulta simulada: um estudo transversal	10.1016/j.nedt.2022.105382
36	Usando a telessaúde para prestar cuidados primários a adolescentes durante e após a pandemia de COVID-19: estudo de pesquisa nacional de nós, profissionais de cuidados primários	https://www.jmir.org/2021/9/e31240
37	Avaliação econômica da consultoria por e-mail e seu impacto no COVID-19. Uma experiência de cinco anos	https://www.scopus.com/inward/record.uri?eid=2-s2.0-85105256242&partnerID=40&md5=d4193e222520c3afdd9b56969fafd2a4
38	Fatores associados à implementação bem-sucedida do aborto por telessaúde em 4 ambientes de prática clínica dos Estados Unidos	10.1016/j.contracepção.2021.04.021
39	Cinco etapas para integrar a telessaúde nos currículos da APRN	10.1016/j.nurpra.2020.12.004
40	Como o COVID-19 afetou as consultas de clínica geral e a renda: evidência de pesquisa populacional transversal de clínico geral da Irlanda	10.1136/bmjopen-2020-044685
41	Iniciando a medicação para transtorno do uso de opióides via telemedicina durante o COVID-19: implicações para as reformas propostas para a Lei Ryan Haight	10.1007/s11606-021-07174-w
42	O efeito adverso da pandemia de COVID-19 no uso de serviços de saúde entre pacientes com diabetes tipo 2 na Carélia do Norte, Finlândia	10.1186/s12913-022-08105-z
43	Experiências de enfermeiros de saúde primários australianos no uso de telessaúde durante o COVID-19: um estudo qualitativo	10.1136/bmjopen-2021-049095
44	Mudanças nas práticas de telessaúde na atenção primária em New Brunswick (Canadá): um estudo comparativo pré e durante a pandemia de COVID-19	10.1371/jornal.pone.0258839
45	Teledermatologia para reduzir consultas presenciais na clínica geral durante a pandemia de COVID-19: Um projeto de melhoria da qualidade	10.1136/bmjoq-2021-001789
46	Educar os profissionais de saúde sobre a COVID-19 com a telementoring ECHO	10.1016/j.ajic.2021.10.030
47	Protocolo de um estudo multifásico sobre telemedicina para idosos na atenção primária	10.1136/bmjopen-2021-057061
48	Impacto do COVID-19 no acesso dos migrantes aos cuidados primários e implicações para a implementação de vacinas: um estudo qualitativo nacional	10.3399/BJGP.2021.0028
49	Lições aprendidas com a pandemia - podemos fazer melhor	10.1891/CL-2021-0010

50	O impacto do COVID-19 no desempenho dos prestadores de serviços de atenção primária à saúde em um sistema de pagamento por capitação: um estudo de caso da Polônia	10.3390/ijerph18041407
51	Percepções de estudantes de enfermagem de uma visita piloto de telessaúde gerontológica simulada	10.3928/01484834-20210616-10
52	Impacto nas atividades de saúde e na saúde dos profissionais da Atenção Primária durante o confinamento	10.35667/MetasEnf.2021.24.1003081780
53	Implementação de serviços de monitoramento digital durante a pandemia de COVID-19 para pacientes com doenças crônicas: abordagem da ciência do design	10.2196/24181
54	O valor do exame físico na era da telemedicina	10.4997/JRCPE.2021.122
55	Uma implementação just-in-time de um programa de extensão para resultados de saúde comunitária (echo) para apoio à atenção primária durante a pandemia de covid-19	10.3122/JABFM.2021.03.200563
56	Projetando para um trabalho em equipe multidisciplinar de atenção primária eficaz e seguro: usando o tempo de covid-19 como estudo de caso	10.3390/ijerph18168758
57	Expansão dos serviços de farmácia durante o COVID-19: farmacêuticos e extensores de farmácias preenchendo as lacunas por meio de serviços de telessaúde	10.1177/00185787211032360
58	Características de alta e transições de cuidados de pacientes hospitalizados com COVID-19	10.1016/j.hjdsi.2020.100512
59	Um aplicativo baseado na Web para a qualidade da espirometria em um sistema de saúde público. Acompanhamento de 10 anos	10.1016/j.arbres.2021.04.026
60	Desafios para unidades de asma em resposta ao COVID-19: uma análise qualitativa de dinâmica de grupo	10.1080/02770903.2021.1917605
61	Geriatria de ligação com lares de idosos em tempo de COVID. Um novo modelo de coordenação chegou para ficar	10.1016/j.regg.2021.01.002
62	Cuidados comunitários de fim de vida durante a pandemia de COVID-19: resultados de uma pesquisa de cuidados primários do Reino Unido	10.3399/BJGPO.2021.0095
63	Explorando o uso e os desafios da implementação de visitas virtuais durante a COVID-19 na atenção primária e lições para uso sustentado	10.1371/jornal.pone.0253665
64	COVID-19 Fontes de Desengajamento no Trabalho de Enfermeiros de Atenção Primária no Malawi	https://www.scopus.com/inward/record.uri?eid=2-s2.0-85101808834&partnerID=40&md5=28cbb995c5dbc756e58420ba5abf9f1d
65	Implementação de consultoria remota na atenção primária do Reino Unido após a pandemia de COVID-19: um estudo longitudinal de métodos mistos	10.3399/BJGP.2020.0948
66	Experiências de gerenciamento de enfermeiras precoces dos hubs COVID-19 finlandeses: uma revisão em ação	10.3390/ijerph19084885
67	Análise Integrando saúde pública e atenção primária: a resposta de seis países da Ásia-Pacífico à pandemia de COVID-19	10.3399/bjgp21X716417

68	Desenvolvimento de um serviço de teledermatologia na zona rural do estado de Washington usando o conjunto de habilidades de prática de enfermagem e ciência de implementação	10.1097/JDN.0000000000000625
69	Saúde mental dos profissionais de enfermagem durante a pandemia de COVID-19: um estudo transversal	10.11606/s1518-8787.2022056004122
70	Prontidão em Saúde e Enfermagem na Atenção Primária Utilizando a Teoria do Cuidado Burocrático: Transformando o Nunca no Agora	10.20467/HumanCaring-D-20-00046
71	Intervenções de enfermagem aumentam como medidas de qualidade da prevenção contra influenza para pacientes de telessaúde domiciliar	10.1097/NCQ.0000000000000577
72	Experiências dos pacientes de “longo COVID” na comunidade e recomendações para melhorar os serviços: uma pesquisa de melhoria da qualidade	10.1177/21501327211041846
73	A experiência dos enfermeiros na utilização da videoconsulta num ambiente de cuidados digitais e o seu impacto no seu fluxo de trabalho e comunicação	10.1371/jornal.pone.0264876
74	Preenchendo uma cavidade no atendimento odontológico	10.1109/MPULS.2021.3066720
75	Mudanças na prática clínica no monitoramento da hipertensão no início da pandemia de COVID-19	10.1093/ajh/hpac049
76	Entrevista para perspectivas sobre a era pós-COVID-19	https://www.scopus.com/inward/record.uri?eid=2-s2.0-85091125298&partnerID=40&md5=6f92ba9fac91976504404a0b03c5946d
77	Transformando os papéis interprofissionais durante a assistência à saúde virtual: o papel em evolução do assistente médico, em relação aos padrões nacionais de competência da profissão de saúde	10.1177/21501327211004285
78	No atendimento clínico, o que a Amazon entregará?	10.1056/NEJMp2113702
79	O que a prática geral pode aprender com as experiências dos enfermeiros de cuidados primários e dos assistentes de saúde da pandemia de COVID-19? Um estudo qualitativo	10.1136/bmjopen-2021-055955
80	Cuidando de mulheres com transtornos por uso de substâncias durante a gravidez e pós-parto durante a pandemia de COVID-19: Lições aprendidas com estagiários de psicologia em um programa de tratamento ambulatorial integrado de ginecologia/uso de substâncias	10.1016/j.jsat.2020.108200
81	Determinantes da intenção dos profissionais da atenção primária pública catalã em usar consultas clínicas digitais (eConsulta) no contexto pós-COVID-19: estudo de métodos mistos	10.2196/28944
82	Mudanças nas apresentações com características potencialmente indicativas de câncer na atenção primária durante a pandemia de COVID-19: um estudo de coorte retrospectivo	10.1136/bmjopen-2021-050131

83	Caracterização e identificação de variações nos tipos de consultas de atenção primária antes e durante a pandemia de covid-19 na Catalunha: estudo de análise de big data	10.2196/29622
84	O impacto da pandemia de COVID nos serviços de atenção primária ao diabetes no Reino Unido: uma pesquisa nacional transversal de pontos de vista dos profissionais de saúde que prestam cuidados ao diabetes	10.1016/j.pcd.2021.12.015
85	Alavancando equipes interdisciplinares para planejamento pré-visita para melhorar as taxas de imunização pneumocócica entre práticas de subespecialidade de medicina interna	10.1177/21501319211060986
86	Um novo programa integrado de telemonitoramento em larga escala para COVID-19	10.1089/tmj.2020.0384
87	Parceria da Feira de Telessaúde COVID-19 para Capacitação em Enfermagem de Atenção Primária	10.1097/NAQ.0000000000000517
88	Implementação do telemonitoramento COVID-19: repercussões na formação acadêmica de Enfermagem.	10.1590/1983-1447.2021.20200395
89	Implementação de visitas de vídeo durante o COVID-19: lições aprendidas de uma prática de atenção primária na cidade de Nova York	10.3389/fpubh.2020.00514
90	Insights da implantação rápida de um "hospital virtual" como padrão durante a pandemia de covid-19	10.7326/M20-4076
91	Gerenciamento desafiador de distúrbios crônicos graves em situação de pandemia aguda: doença hepática crônica sob pandemia de COVID-19 como modelo de prova de princípio para orquestrar as medidas no contexto das 15h	10.1007/s13167-021-00231-8
92	Experiências dos profissionais da atenção primária durante a primeira onda da pandemia de COVID-19 na Grécia: um estudo qualitativo	10.1186/s12875-021-01522-9
93	O impacto da pandemia de COVID-19 no futuro da telessaúde na atenção primária	10.1016/j.outlook.2021.09.004
94	Avaliação das pandemias de HIV-virológica virológica durante a atendimento amtorial interdisciplinar do HIV	10.1002/jac5.1422
95	Enfrentando os Desafios da COVID-19: Avaliação das Mudanças Lideradas por Enfermeiros na Avaliação Telefônica	10.1097/NHH.0000000000001081
96	Avaliação qualitativa da transformação rápida do sistema para visitas de vídeo de atenção primária em um centro médico acadêmico	10.7326/M20-1814
97	Gestão clínica de cuidados primários após automutilação durante a primeira onda de COVID-19 no Reino Unido: estudo de coorte baseado na população	10.1136/bmjopen-2021-052613
98	Envolvimento do farmacêutico em um programa abrangente de monitoramento remoto e telegestão	10.1093/ajhp/zxac025

99	Impacto percebido do COVID-19 na atenção primária na Nova Inglaterra: um estudo qualitativo	10.3122/jabfm.2022.02.210317
100	Características das consultas telefônicas gratuitas relacionadas à covid-19 por enfermeiros de saúde pública no japão: um estudo retrospectivo	10.3390/saúde 9081022
101	A telemedicina aumenta o acesso à iniciação da buprenorfina durante a pandemia de COVID-19	10.1016/j.jsat.2020.108272
102	Aproveitando a telemedicina para a prestação de cuidados de saúde: análise bibliométrica e cienciométrica.	10.2196/18835
103	Implicações da política de telessaúde para enfermeiras registradas na atenção primária	10.1177/1357633X20940142
104	Um novo programa de cuidados colaborativos para aumentar os cuidados domiciliares de enfermagem durante e após a pandemia de COVID-19	10.1016/j.jamda.2021.11.018
105	Relação entre alfabetização em eSaúde e estado psicológico durante a pandemia de COVID-19: uma pesquisa com residentes chineses	10.1111/jonm.13221

64 ARTIGOS ENCONTRADOS NA BASE DE DADOS WOS

Nº	TÍTULO DO ARTIGO	LINK DE ACESSO/DOI
1	Uma plataforma de base tecnológica para avaliação de risco, detecção e prevenção de quedas entre idosos domiciliares: protocolo para um estudo quase experimental	https://pubmed.ncbi.nlm.nih.gov/34387557/
2	O papel dos cuidados primários durante a pandemia de COVID-19: uma visão geral europeia	https://www.sciencedirect.com/science/article/pii/S0212656721001682
3	Avaliação da disponibilidade de saúde sexual e reprodutiva para mulheres migrantes venezuelanas durante a pandemia de SARS-CoV-2 na fronteira noroeste do Brasil-Venezuela	10.1016/j.jmh.2022.100092
4	Treinamento de resolução de problemas para veteranos com comorbidades complexas: adaptações de entrega de tratamento durante o COVID-19	10.1080/07317115.2021.1963382
5	Resultados da doença de coronavírus 2019 em lares de idosos franceses que implementaram o confinamento de funcionários com residentes	10.1001/jamannetworkopen.2020.17533
6	Infecção por SARS-CoV-2 em ambientes profissionais na Catalunha	Não disponível
7	Enfermeiros Psiquiátricos como Líderes na Integração da Saúde Comportamental	10.1016/j.nurpra.2020.09.001
8	Vulnerabilidades da saúde infantil durante a pandemia de COVID-19 no Brasil e em Portugal	10.1590/1518-8345.4805.3422
9	Experiências de cirurgiões veterinários, enfermeiros veterinários e proprietários de consultas de telemedicina felina durante a pandemia de COVID-19 de 2020	10.1002/vetr.1738
10	Gestão no cuidado às pessoas com HIV na atenção primária à saúde em tempos do novo coronavírus	10.11606/s1518-8787.2022056003876
11	Lançamento de um serviço de telemedicina e linha direta de atendimento primário COVID-19 em todo o estado	10.3122/jabfm.2021.S1.200178
12	Experiência do paciente de telemedicina para tratamento de dor de cabeça durante a pandemia de COVID-19: um estudo de pesquisa da American Migraine Foundation	10.1111/cabeça.14110
13	Utilização de telessaúde em instituições residenciais de cuidados a idosos durante a pandemia de COVID-19: um estudo de coorte retrospectivo na prática geral australiana	10.1177/1357633X221094406
14	O impacto do COVID-19 nos cuidados crônicos de acordo com os provedores: um estudo qualitativo entre as práticas de atenção primária na Bélgica	10.1186/s12875-020-01326-3
15	Desenvolvendo uma compreensão do conhecimento, atitudes e práticas dos adolescentes em relação ao COVID-19	10.1177/1059840520957069
16	Protocolo de pesquisa para explorar o uso de vídeo e telefone na clínica geral de Aotearoa Nova Zelândia: considerações para a futura telessaúde	10.1136/bmjhci-2020-100309

17	Expansão da telessaúde na atenção primária durante a pandemia de COVID-19: benefícios e barreiras	10.1097/JXX.0000000000000626
18	Avaliação da comunicação de estudantes de enfermagem na teleconsulta simulada: um estudo transversal	10.1016/j.nedt.2022.105382
19	"Em casa, com cuidado": lições das práticas de cuidados primários domiciliares de Nova York Gerenciando o COVID-19	10.1111/jgs.16952
20	"Há algo muito pessoal em ver o rosto de alguém": percepções do provedor de visitas de vídeo na atenção primária domiciliar durante o COVID-19	10.1177/07334648211028393
21	Usando a telessaúde para prestar cuidados primários a adolescentes durante e após a pandemia de COVID-19: estudo de pesquisa nacional de profissionais de cuidados primários dos EUA	10.2196/31240
22	Cinco etapas para integrar a telessaúde nos currículos da APRN	10.1016/j.nurpra.2020.12.004
23	Como o COVID-19 afetou as consultas de clínica geral e a renda: evidência de pesquisa populacional transversal de clínico geral da Irlanda	10.1136/bmjopen-2020-044685
24	Iniciando a medicação para transtorno do uso de opióides via telemedicina durante o COVID-19: implicações para as reformas propostas para a Lei Ryan Haight	10.1007/s11606-021-07174-w
25	O efeito adverso da pandemia de COVID-19 no uso de serviços de saúde entre pacientes com diabetes tipo 2 na Carélia do Norte, Finlândia	10.1186/s12913-022-08105-z
26	Experiências de enfermeiros de saúde primários australianos no uso de telessaúde durante o COVID-19: um estudo qualitativo	10.1136/bmjopen-2021-049095
27	Mudanças nas práticas de telessaúde na atenção primária em New Brunswick (Canadá): um estudo comparativo pré e durante a pandemia de COVID-19	10.1371/jornal.pone.0258839
28	Saúde mental dos profissionais de enfermagem durante a pandemia de COVID-19: um estudo transversal	10.11606/s1518-8787.2022056004122
29	Educar os profissionais de saúde sobre a COVID-19 com a telementoring ECHO	10.1016/j.ajic.2021.10.0300196-6553
30	Protocolo de um estudo multifásico sobre telemedicina para idosos na atenção primária	10.1136/bmjopen-2021-057061
31	Lições aprendidas com a pandemia - podemos fazer melhor	10.1891/CL-2021-0010
32	Percepções do estudante de enfermagem de uma visita piloto de telessaúde gerontológica simulada	10.3928/01484834-20210616-10
33	Implementação de serviços de monitoramento digital durante a pandemia de COVID-19 para pacientes com doenças crônicas: abordagem da ciência do design	10.2196/24181

34	Implementando a triagem remota em grandes sistemas de saúde: uma síntese de evidências qualitativas	10.1002/nr.22093
35	Expansão dos serviços de farmácia durante o COVID-19: farmacêuticos e extensores de farmácias preenchendo as lacunas por meio de serviços de telessaúde	10.1177/00185787211032360
36	COVID-19 Fontes de Desengajamento no Trabalho de Enfermeiros de Atenção Primária no Malawi	Não disponível
37	Implementação de consultoria remota na atenção primária do Reino Unido após a pandemia de COVID-19: um estudo longitudinal de métodos mistos	10.3399/BJGP.2020.0948
38	Desenvolvimento de um serviço de teledermatologia na zona rural do estado de Washington usando o conjunto de habilidades de prática de enfermagem e ciência de implementação	10.1097/JDN.0000000000000625
39	Percepção dos profissionais de enfermagem sobre a autoridade temporária de prática plena durante um surto de COVID-19: um estudo qualitativo	10.1016/j.ijnurstu.2021.104141
40	Conscientização sobre o COVID-19 e os problemas enfrentados por jovens adultos com diabetes mellitus tipo 1 em meio ao bloqueio nacional na Índia: um estudo de entrevista qualitativa	10.1016/j.pcd.2020.07.001
41	Percepção de usuários sobre violência e conflitos com profissionais de unidades básicas de saúde antes e durante a COVID-19. Um estudo qualitativo	10.3389/fpubh.2021.810014
42	Intervenções de enfermagem aumentam as medidas de qualidade da vacinação contra a gripe para pacientes de telessaúde domiciliar	10.1097/NCQ.0000000000000577
43	Experiências dos pacientes de "longo COVID" na comunidade e recomendada para melhorar os serviços: uma de melhoria da qualidade	10.1177/21501327211041846
44	Desafios para unidades de asma em resposta ao COVID-19: uma análise qualitativa de dinâmica de grupo	10.1080/02770903.2021.1917605
45	Mudanças na prática clínica no monitoramento da hipertensão no início da pandemia de COVID-19	10.1093/ajh/hpac049
46	O que a prática geral pode aprender com as experiências dos enfermeiros de cuidados primários e dos assistentes de saúde da pandemia de COVID-19? Um estudo qualitativo	10.1136/bmjopen-2021-055955
47	Cuidando de mulheres com transtornos por uso de substâncias durante a gravidez e pós-parto durante a pandemia de COVID-19: lições aprendidas com estagiários de psicologia em um programa de tratamento ambulatorial integrado de ginecologia/transtorno por uso de substâncias	10.1016/j.jsat.2020.108200
48	Determinantes da intenção dos profissionais da atenção primária pública catalã em usar consultas clínicas digitais (eConsulta) no contexto pós-COVID-19: estudo de métodos mistos	10.2196/28944

49	Caracterização e identificação de variações nos tipos de consultas de atenção primária antes e durante a pandemia de COVID-19 na Catalunha: estudo de análise de big data	10.2196/29622
50	O impacto da pandemia de COVID nos serviços de atenção primária ao diabetes no Reino Unido: uma pesquisa nacional transversal de pontos de vista dos profissionais de saúde que prestam cuidados ao diabetes	10.1016/j.pcd.2021.12.015
51	Alavancando equipes interdisciplinares para planejamento pré-visita para melhorar as taxas de imunização pneumocócica entre práticas de subespecialidade de medicina interna	10.1177/21501319211060986
52	Um novo programa integrado de telemonitoramento em larga escala para COVID-19	10.1089/tmj.2020.0384
53	Implementação de visitas de vídeo durante o COVID-19: lições aprendidas de uma prática de atenção primária na cidade de Nova York	10.3389/fpubh.2020.00514
54	Gerenciamento desafiador de distúrbios crônicos graves em situação de pandemia aguda: doença hepática crônica sob pandemia de COVID-19 como modelo de prova de princípio para orquestrar as medidas no contexto das 15h	10.1007/s13167-021-00231-8
55	Experiências dos profissionais da atenção primária durante a primeira onda da pandemia de COVID-19 na Grécia: um estudo qualitativo	10.1186/s12875-021-01522-9
56	O impacto da pandemia de COVID-19 no futuro da telessaúde no cuidado	10.1016/j.outlook.2021.09.004
57	Envolvimento do farmacêutico em um programa abrangente de monitoramento remoto e telegestão	10.1093/ajhp/zxac025
58	Impacto percebido do COVID-19 na atenção primária na Nova Inglaterra: um estudo qualitativo	10.3122/jabfm.2022.02.210317
59	Características das consultas telefônicas gratuitas relacionadas à COVID-19 por enfermeiras de saúde pública no Japão: um estudo retrospectivo	10.3390/saúde 9081022
60	Estado atual da pesquisa em psicoterapia para idosos vulneráveis que vivem em casa com depressão	10.1007/s00391-020-01805-3
61	Profissionais de enfermagem em diabetes à sombra da pandemia de COVID-19: desafios, insights e sugestões para melhoria	10.1111/jnu.12754
62	Aproveitando a Telemedicina para a Prestação de Cuidados de Saúde: Análise Bibliométrica e Cienciométrica	10.2196/18835

63	Implicações da política de telessaúde para enfermeiras registradas na atenção primária	10.1177/1357633X20940142
64	Percepções dos profissionais de saúde sobre qualidade, aceitação e satisfação com os serviços de saúde telecomportamentais durante a pandemia de COVID-19: estudo baseado em pesquisa	10.2196/23245

96 ARTIGOS ENCONTRADOS NA BASE DE DADOS EMBASE

Nº	TÍTULO DO ARTIGO	LINK DE ACESSO/DOI
1	Programa de Simulação de Comunicação Estruturada Telefônica para Acompanhamento de Casos e Contatos de COVID-19 na Atenção Básica	10.3390/ijerph19073915
2	Uma pesquisa transversal sobre práticas de gerenciamento de medicamentos para doenças não transmissíveis na Europa durante a segunda onda da pandemia de COVID-19	10.3389/ffar.2021.685696
3	Avaliações interativas de saúde mental para chineses canadenses: um estudo piloto randomizado controlado em uma clínica de cuidados primários liderada por enfermeiros	10.1111/app.12400
4	Prescrição antecipada em cuidados comunitários de fim de vida no Reino Unido e na Irlanda durante a pandemia de COVID-19: pesquisa online	10.1136/bmjspcare-2020-002394
5	O papel dos cuidados primários durante a pandemia de COVID-19: uma visão geral europeia	10.1016/j.aprim.2021.102134
6	Gerenciamento de casos na linha de frente do COVID-19: a importância do plano de atendimento individualizado em todos os ambientes de atendimento	10.1097/NCM.0000000000000484
7	Ações e adaptações implementadas para a prestação de serviços de saúde materna, neonatal e infantil durante a fase inicial da pandemia de COVID-19 em Lagos, Nigéria: estudo qualitativo dos líderes das unidades de saúde	10.5334/aogh.3529
8	Resultados da doença de coronavírus 2019 em lares de idosos franceses que implementaram o confinamento de funcionários com residentes	10.1001/jamannetworkopen.2020.17533
9	Acesso a serviços de saúde mental de refugiados e durante os primeiros seis meses da pandemia de covid-19: uma pesquisa de médicos de refugiados-19	10.3390/ijerph18105266
10	Cuidados de fim de vida durante a COVID-19: oportunidades e desafios para a enfermagem comunitária	10.12968/bjcn.2021.26.1.44
11	Gestão no cuidado às pessoas com HIV na atenção primária à saúde em tempos do novo coronavírus	10.11606/S1518-8787.2022056003876
12	Lançamento de uma linha direta de atendimento primário COVID-19 em todo o estado e serviço de telemedicina	10.3122/JABFM.2021.S1.200178
13	Experiência do paciente de telemedicina para tratamento de dor de cabeça durante a pandemia de COVID-19: um estudo de pesquisa da American Migraine Foundation	10.1111/cabeça.14110
14	Tendências de prescrição de antibióticos na atenção primária fora de horas belga durante a pandemia de covid-19: estudo observacional usando dados de saúde coletados rotineiramente	10.3390/antibióticos10121488
15	Explorando os efeitos da pandemia de COVID-19 nas crianças e famílias cuidadas por enfermeiras registradas de prática avançada com foco em pediatria	10.1016/j.pedhc.2022.01.003

16	Reorganização dos serviços de atenção primária durante a covid-19 no cabo ocidental, áfrica do sul: perspectivas dos enfermeiros da atenção primária	10.4102/safp.v63i1.5358
17	O impacto do COVID-19 nos cuidados crônicos de acordo com os provedores: um estudo qualitativo entre as práticas de atenção primária na Bélgica	10.1186/s12875-020-01326-3
18	Desenvolvendo uma compreensão do conhecimento, atitudes e práticas dos adolescentes em relação ao COVID-19	10.1177/1059840520957069
19	Protocolo de pesquisa para explorar o uso de vídeo e telefone na clínica geral de Aotearoa Nova Zelândia: Considerações para a futura telessaúde	10.1136/bmjhci-2020-100309
20	Um roteiro da Rede de Pesquisa Baseada na Prática (PBRN) para avaliar o COVID-19 em centros de saúde comunitários: um relatório do OCHIN PBRN	10.3122/JABFM.2020.05.200053
21	Expansão da telessaúde na atenção primária durante a pandemia de COVID-19: benefícios e barreiras	10.1097/JXX.0000000000000626
22	“Em casa, com cuidado”: lições das práticas de cuidados primários domiciliares da cidade de Nova York Gerenciando o COVID-19	10.1111/jgs.16952
23	“Há algo muito pessoal em ver o rosto de alguém”: percepções do provedor de visitas de vídeo na atenção primária domiciliar durante o COVID-19	10.1177/07334648211028393
24	Uma telemedicina de dois gumes para cuidados maternos durante o COVID-19: resultados de um estudo global de métodos mistos de profissionais de saúde	10.1136/bmjgh-2020-004575
25	Planejamento de cuidado baseado em equipe virtual com idosos em ambientes de cuidados formais: um protocolo de revisão de escopo	10.1136/bmjopen-2021-054900
26	Avaliação da comunicação de estudantes de enfermagem na teleconsulta simulada: um estudo transversal	10.1016/j.nedt.2022.105382
27	Usando a telessaúde para prestar cuidados primários a adolescentes durante e após a pandemia de COVID-19: estudo de pesquisa nacional de nós, profissionais de cuidados primários	10.2196/31240
28	Fatores associados à implementação bem-sucedida do aborto por telessaúde em 4 ambientes de prática clínica dos Estados Unidos	10.1016/j.contracepção.2021.04.021
29	Como a telessaúde atende às necessidades de idosos vulneráveis	10.3122/jabfm.2022.03.210493
30	Como o COVID-19 afetou as consultas de clínica geral e a renda: evidência de pesquisa populacional transversal de clínico geral da Irlanda	10.1136/bmjopen-2020-044685

31	Iniciando a medicação para transtorno do uso de opióides via telemedicina durante o COVID-19: implicações para as reformas propostas para a Lei Ryan Haight	10.1007/s11606-021-07174-w
32	O efeito adverso da pandemia de COVID-19 no uso de serviços de saúde entre pacientes com diabetes tipo 2 na Carélia do Norte, Finlândia	10.1186/s12913-022-08105-z
33	Experiências de enfermeiros de saúde primários australianos no uso de telessaúde durante o COVID-19: um estudo qualitativo	10.1136/bmjopen-2021-049095
34	Mudanças nas práticas de telessaúde na atenção primária em New Brunswick (Canadá): um estudo comparativo pré e durante a pandemia de COVID-19	10.1371/jornal.pone.0258839
35	Teledermatologia para reduzir consultas presenciais na clínica geral durante a pandemia de COVID-19: Um projeto de melhoria da qualidade	10.1136/bmjoq-2021-001789
36	Educar os profissionais de saúde sobre a COVID-19 com a telementoring ECHO	10.1016/j.ajic.2021.10.030
37	Protocolo de um estudo multifásico sobre telemedicina para idosos na atenção primária	10.1136/bmjopen-2021-057061
38	Impacto do COVID-19 no acesso dos migrantes aos cuidados primários e implicações para a implementação de vacinas: um estudo qualitativo nacional	10.3399/BJGP.2021.0028
39	O impacto do COVID-19 no desempenho dos prestadores de serviços de atenção primária à saúde em um sistema de pagamento por capitação: um estudo de caso da Polônia	10.3390/ijerph18041407
40	Percepções do estudante de enfermagem de uma visita piloto de telessaúde gerontológica simulada	10.3928/01484834-20210616-10
41	Implementação de serviços de monitoramento digital durante a pandemia de COVID-19 para pacientes com doenças crônicas: abordagem da ciência do design	10.2196/24181
42	O valor do exame físico na era da telemedicina	10.4997/JRCPE.2021.122
43	Uma implementação just-in-time de um programa de extensão para resultados de saúde comunitária (echo) para apoio à atenção primária durante a pandemia de covid-19	10.3122/JABFM.2021.03.200563
44	Expansão dos serviços de farmácia durante o COVID-19: farmacêuticos e extensores de farmácias preenchendo as lacunas por meio de serviços de telessaúde	10.1177/00185787211032360
45	Características de alta e transições de cuidados de pacientes hospitalizados com COVID-19	10.1016/j.hjdsi.2020.100512
46	Reimaginando a Enfermagem Escolar: Lições Aprendidas de uma Enfermeira Escolar Virtual	10.1177/1942602X21996432
47	Desafios para unidades de asma em resposta ao COVID-19: uma análise qualitativa de dinâmica de grupo	10.1080/02770903.2021.1917605

48	Geriatria de ligação com lares de idosos em tempo de COVID. Um novo modelo de coordenação chegou para ficar	10.1016/j.regg.2021.01.002
49	Implementação de consultoria remota na atenção primária do Reino Unido após a pandemia de COVID-19: um estudo longitudinal de métodos mistos	10.3399/BJGP.2020.0948
50	Experiências de gerenciamento de enfermeiras precoces dos hubs COVID-19 finlandeses: uma revisão em ação	10.3390/ijerph19084885
51	Análise Integrando saúde pública e atenção primária: a resposta de seis países da Ásia-Pacífico à pandemia de COVID-19	10.3399/bjgp21X716417
52	Desenvolvimento de um serviço de teledermatologia na zona rural do estado de Washington usando o conjunto de habilidades de prática de enfermagem e ciência de implementação	10.1097/JDN.0000000000000625
53	Saúde mental dos profissionais de enfermagem durante a pandemia de COVID-19: um estudo transversal	10.11606/s1518-8787.2022056004122
54	Usando um sistema de localização em tempo real para avaliar o impacto da telemedicina em um departamento de emergência durante o COVID-19: estudo observacional	10.2196/29240
55	Um papel para o educador de enfermagem em diabetes para apoiar telemedicinamente crianças com diabetes tipo 1 no monitoramento contínuo da glicose? A experiência de bloqueio COVID-19	10.1016/j.pcd.2022.03.011
56	Intervenções de enfermagem aumentam as medidas de qualidade da vacinação contra a gripe para pacientes de telessaúde domiciliar	10.1097/NCQ.0000000000000577
57	Experiências dos pacientes de "longo COVID" na comunidade e recomendações para melhorar os serviços: uma pesquisa de melhoria da qualidade	10.1177/21501327211041846
58	A experiência dos enfermeiros na utilização da videoconsulta num ambiente de cuidados digitais e o seu impacto no seu fluxo de trabalho e comunicação	10.1371/jornal.pone.0264876
59	Mudanças na prática clínica no monitoramento da hipertensão no início da pandemia de COVID-19	10.1093/ajh/hpac049
60	Transformando os papéis interprofissionais durante a assistência à saúde virtual: o papel em evolução do assistente médico, em relação aos padrões nacionais de competência da profissão de saúde	10.1177/21501327211004285
61	No atendimento clínico, o que a Amazon entregará?	10.1056/NEJMp2113702
62	O que a prática geral pode aprender com as experiências dos enfermeiros de cuidados primários e dos assistentes de saúde da pandemia de COVID-19? Um estudo qualitativo	10.1136/bmjopen-2021-055955
63	Cuidando de mulheres com transtornos por uso de substâncias durante a gravidez e pós-parto durante a pandemia de COVID-19: lições aprendidas com estagiários de psicologia em um programa de tratamento ambulatorial integrado de ginecologia/transtorno por uso de substâncias	10.1016/j.jsat.2020.108200

64	Determinantes da intenção dos profissionais da atenção primária pública em uso de consultas digitais (eConsulta) no contexto pós-COVID-19: estudo de métodos mistos	10.2196/28944
65	Satisfação com a Saúde nos Cuidados de Saúde entre Pacientes do 3º Centro Terciário de Alergia em Kirkuk com Pacientes do Centro de Atenção Primária à Saúde de Salam em Kirkuk	10.37506/IJFMT.V16I1.17436
66	Mudanças nas apresentações com características potencialmente indicativas de câncer na atenção primária durante a pandemia de COVID-19: um estudo de coorte retrospectivo	10.1136/bmjopen-2021-050131
67	Caracterização e identificação de variações nos tipos de consultas de atenção primária antes e durante a pandemia de covid-19 na Catalunha: estudo de análise de big data	10.2196/29622
68	O impacto da pandemia de COVID nos serviços de atenção primária ao diabetes no Reino Unido: uma pesquisa nacional transversal de pontos de vista dos profissionais de saúde que prestam cuidados ao diabetes	10.1016/j.pcd.2021.12.015
69	Alavancando equipes interdisciplinares para planejamento pré-visita para melhorar as taxas de imunização pneumocócica entre práticas de subespecialidade de medicina interna	10.1177/21501319211060986
70	Um novo programa integrado de telemonitoramento em larga escala para COVID-19	10.1089/tmj.2020.0384
71	Parceria da Feira de Telessaúde COVID-19 para Capacitação em Enfermagem de Atenção Primária	10.1097/NAQ.0000000000000517
72	Implementação do telemonitoramento COVID-19: repercussões na formação acadêmica de Enfermagem.	10.1590/1983-1447.2021.20200395
73	Gerenciamento desafiador de distúrbios crônicos graves em situação de pandemia aguda: doença hepática crônica sob pandemia de COVID-19 como modelo de prova de princípio para orquestrar as medidas no contexto das 15h	10.1007/s13167-021-00231-8
74	Viabilidade e aceitabilidade do detalhamento acadêmico virtual sobre prescrição de opióides	10.1016/j.ijmedinf.2020.104365
75	Experiências dos profissionais da atenção primária durante a primeira onda da pandemia de COVID-19 na Grécia: um estudo qualitativo	10.1186/s12875-021-01522-9
76	Uma nova transição: lições aprendidas durante a rápida implementação e evolução do tratamento com opióides baseado em grupo de telessaúde (t-GBOT) durante a pandemia de COVID-19	10.1016/j.hjdsi.2021.100559
77	Avaliação das taxas de supressão virológica durante a pandemia de COVID-19 com atendimento ambulatorial interdisciplinar de HIV	10.1002/jac5.1422
78	Enfrentando os Desafios da COVID-19: Avaliação das Mudanças Lideradas por Enfermeiros na Avaliação Telefônica	10.1097/NHH.0000000000001081

79	Gestão clínica de cuidados primários após automutilação durante a primeira onda de COVID-19 no Reino Unido: estudo de coorte baseado na população	10.1136/bmjopen-2021-052613
80	Envolvimento do farmacêutico em um programa abrangente de monitoramento remoto e telegestão	10.1093/ajhp/zxac025
81	Intervenções baseadas em tecnologia para residentes de asilos: um protocolo de revisão sistemática	10.1136/bmjopen-2021-056142
82	Impacto percebido do COVID-19 na atenção primária na Nova Inglaterra: um estudo qualitativo	10.3122/jabfm.2022.02.210317
83	Desencaminhamento rápido e triagem de pacientes em cuidados paliativos baseados na comunidade	10.1016/j.jpainsymman.2020.03.040
84	Medicina de emergência global: uma revisão abrangente da literatura de 2020	10.1111/acem.14356
85	Evidências globais sobre a rápida adoção da telemedicina na atenção primária durante os primeiros 2 anos da pandemia de COVID-19: um protocolo de revisão de escopo	10.1186/s13643-022-01934-3
86	Cuidados percebidos e bem-estar de pacientes com câncer e participantes de normas correspondentes na crise do COVID-19: resultados de uma pesquisa com participantes no registro holandês de perfis	10.1001/jamaoncol.2020.6093
87	Projeto e implementação de uma unidade de enfermagem especializada em COVID-19	10.1016/j.jamda.2021.02.001
88	Mudança no fluxo de trabalho de enfermagem em uma unidade de internação COVID-19 após a implantação da telessaúde de internação: estudo observacional usando um sistema de localização em tempo real	10.2196/36882
89	Protocolo de estudo para a adaptação e avaliação online do programa de coaching 'Reboot' (Recovery-boosting), para preparar enfermeiros de cuidados intensivos e auxiliar na recuperação após eventos clínicos estressantes	10.1186/s40814-022-01014-2
90	Prescrição de medicamentos em consultas presenciais versus consultas de telessaúde durante a pandemia de COVID-19 na clínica geral australiana: um estudo observacional retrospectivo	10.3399/BJGPO.2021.0132
91	A telemedicina aumenta o acesso à iniciação da buprenorfina durante a pandemia de COVID-19	10.1016/j.jsat.2020.108272
92	Aproveitando a telemedicina para a prestação de cuidados de saúde: análise bibliométrica e cienciométrica.	10.2196/18835
93	Implicações da política de telessaúde para enfermeiras registradas na atenção primária	10.1177/1357633X20940142

94	Expandindo os serviços de videoconsulta em ritmo e escala na Escócia durante a pandemia de covid-19: estudo de caso nacional de métodos mistos	10.2196/31374
95	Um novo programa de cuidados colaborativos para aumentar os cuidados domiciliares de enfermagem durante e após a pandemia de COVID-19	10.1016/j.jamda.2021.11.018
96	Relação entre alfabetização em eSaúde e estado psicológico durante a pandemia de COVID-19: uma pesquisa com residentes chineses	10.1111/jonm.13221
