# Supplementary figures and images for: Telenursing contributions in Primary Health Care in the COVID-19 pandemic context: an integrative review
Source: Rev Bras Enferm. 2024 Nov 22;77(5):e20240093. doi: 10.1590/0034-7167-2024-0093 (PMC11653881; doi:10.1590/0034-7167-2024-0093)

**Figura 1** – Fluxograma das etapas de seleção dos estudos, ancorado no PRISMA, 2022

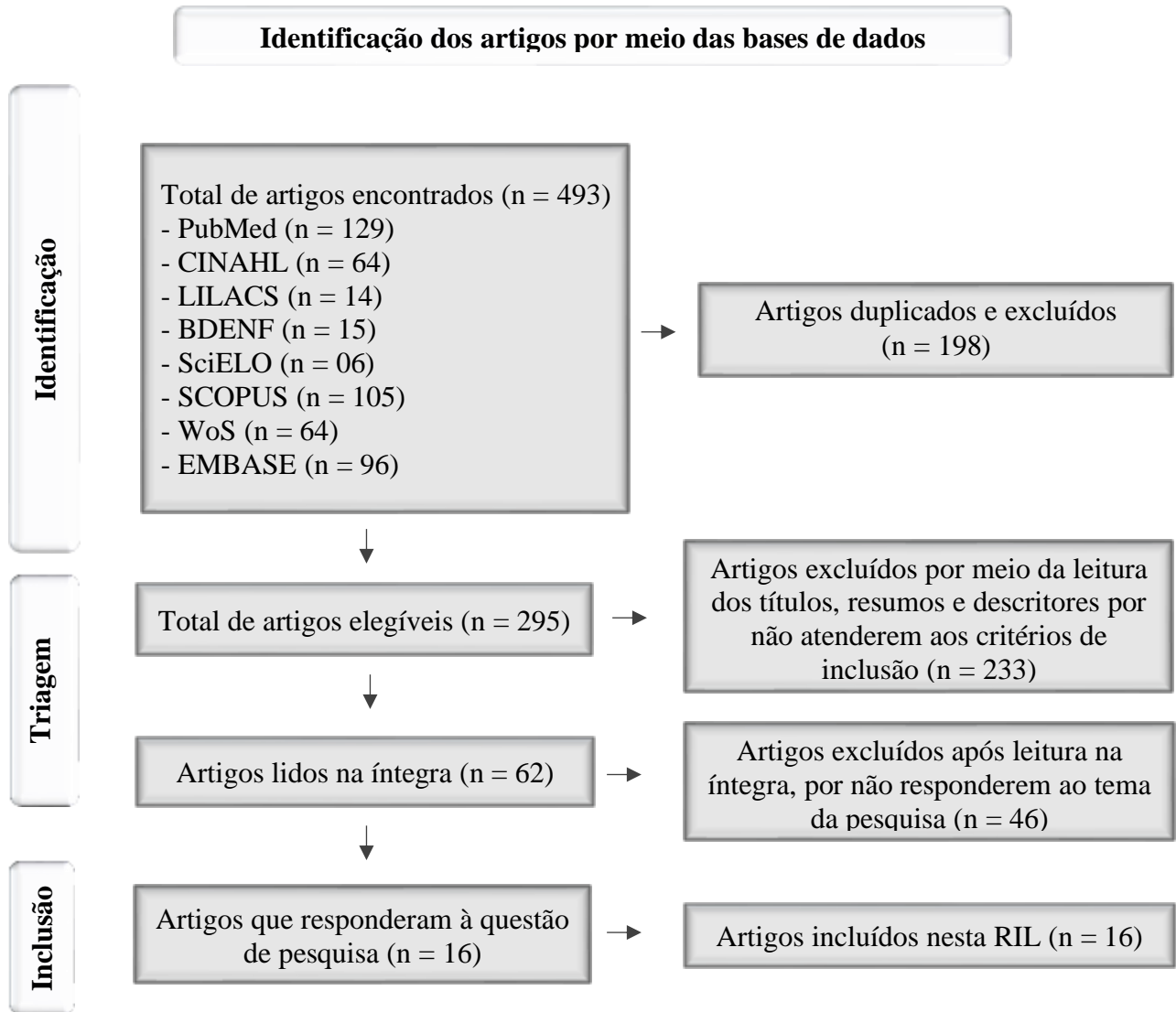

Supplement: Supplementary file 2 [file 0034-7167-reben-77-05-e20240093-suppl02.pdf]
