# Supplementary material for: Telenursing contributions in Primary Health Care in the COVID-19 pandemic context: an integrative review
Source: Rev Bras Enferm. 2024 Nov 22;77(5):e20240093. doi: 10.1590/0034-7167-2024-0093 (PMC11653881; doi:10.1590/0034-7167-2024-0093)
Supplement: Supplementary file 3 [file 0034-7167-reben-77-05-e20240093-suppl03.pdf]

README

Título do conjunto de dados:

- ESTRATEGIA_DE_BUSCA_DETALHADA
- FLUXOGRAMA_PRISMA
- SINTESE_DOS_ARTIGOS_SELECIONADOS

Artigo submetido à REBEn, intitulado: Contribuições da telenfermagem na atenção primária à saúde no contexto pandêmico da covid-19: revisão integrativa.

Informações de contato:

Pesquisador correspondente/principal/responsável pela coleta dos dados:

Patrícia Amidianski, Universidade Federal de Santa Catarina (UFSC), patricia.amidianski@gmail.com

Data de coleta dos dados:

A coleta de dados foi realizada em agosto de 2022.

Visão geral dos dados e arquivos:

O arquivo "ESTRATEGIA_DE_BUSCA_DETALHADA" (criado em 01/07/2022) contém dados detalhados referente a estratégia de busca, como: bases de dados científicas, descritores, filtros, códigos de busca, total de artigos encontrados (discriminados separadamente para cada base de dados utilizada), links de acesso dos artigos, total de artigos selecionados e total de artigos lidos na íntegra.

O arquivo "FLUXOGRAMA_PRISMA" (criado em 01/07/2022) contém dados organizados entre identificação, triagem e inclusão, de acordo com o modelo *Preferred Reporting Items for Systematic Reviews and Meta-Analyses* (PRISMA).

O arquivo "SINTESE_DOS_ARTIGOS_SELECIONADOS" (criado em 01/07/2022) contém dados detalhados acerca do conteúdo dos artigos selecionados, como: número do estudo, referência, país/ano, objetivo, delineamento metodológico, caracterização dos participantes e contribuições da teleconsulta para a assistência em enfermagem.

Os arquivos se relacionam acerca da complementação de dados referente ao tratamento quantitativo e qualitativo, desde o total de artigos encontrados até o total de

artigos selecionados para a revisão, apresentando descrições detalhadas de todo o processo.

Descrição dos métodos de coleta ou geração dos dados:

Trata-se de uma Revisão Integrativa da Literatura, organizada em seis etapas metodológicas, a saber: formulação da questão de pesquisa; estabelecimento de critérios para inclusão e exclusão de estudos; extração dos dados dos estudos; avaliação e análise crítica dos estudos a serem incluídos na revisão; interpretação dos resultados; e apresentação da revisão/síntese do conhecimento. Síntese abaixo:

Etapa1<br>Formulação da questão de pesquisa	Quais as contribuições da telenfermagem na Atenção Primária à Saúde no contexto pandêmico da Covid-19?
Etapa 2<br>Estabelecimento de critérios para inclusão e exclusão de estudos	Critérios de inclusão: artigos científicos primários de abordagem qualitativa e quantitativa, publicados no período de janeiro de 2020 a agosto de 2022, nos idiomas português, inglês e espanhol, que respondessem à questão de pesquisa.<br>Critérios de exclusão: estudos na modalidade de revisões, editoriais, cartas, artigos de opinião, comentários, resumos de anais, publicações duplicadas, dossiês, trabalhos de conclusão de curso, documentos oficiais de programas nacionais e internacionais, relatos de experiência, estudos de reflexão, estudos teóricos, teses, dissertações, boletins epidemiológicos, relatórios de gestão e livros.
Etapa 3<br>Extração dos dados dos estudos	Elaboração de um quadro para organização e síntese dos dados, contendo cinco tópicos, a saber: referência,

	ano/país, base de dados, objetivo principal e tipo de estudo.
Etapa 4<br>Avaliação e análise crítica dos estudos	Procurou-se explicações para os dados obtidos, utilizando-se questionamentos, como: qual o objetivo do estudo? Quem eram os participantes e em que ambiente estavam inseridos? O estudo atingiu seus objetivos? Qual a contribuição do estudo acerca da telenfermagem?
Etapa 5<br>Interpretação dos resultados	Resultou em três categorias temáticas, a saber: "Contribuições da telenfermagem na Atenção Primária à Saúde", "Compilado de pontos positivos e negativos da telenfermagem" e "Perspectivas de consolidação da telenfermagem em um cenário pós-pandêmico".
Etapa 6<br>Síntese do conhecimento	Os dados foram apresentados de modo descritivo, de acordo com as suas categorias, e discutidos à luz da literatura científica pertinente.

Em relação às fontes de dados, foram incluídas as seguintes bases de dados: *Public Medical Literature Analysis and Retrieval System Online* (PubMed), *Cumulative Index to Nursing & Allied Helth Literature* (CINAHL), Literatura Latino-Americana e do Caribe em Ciências da Saúde (LILACS), Base de Dados de Enfermagem (BDENF), *Scientific Electronic Library Online* (SciELO), SCOPUS, *Web of Science* (WoS) e EMBASE. A pesquisa contou com o auxílio do Portal de Periódicos CAPES e da Biblioteca Virtual em Saúde (BVS).

Os termos utilizados para a pesquisa foram selecionados por meio dos Descritores em Ciências da Saúde (DeCS) e do *Medical Subject Headings* (Mesh), a saber: Consulta Remota, Telemedicina, Telenfermagem (*Teleenfermería, Remote Consultation, Telemedicine*, *Telenursing*); Enfermagem (*Enfermeria, Nursing, Nurses*); Atenção Primária à Saúde (*Atención Primaria de Salud, Primary Health Care, Enfermagem de*

Atenção Primária, Enfermería de Atención Primaria, Primary Care Nursing, Enfermagem Primária, Enfermería Primaria, Primary Nursing); e Covid-19. Estratégia de busca detalhada no **arquivo "ESTRATEGIA_DE_BUSCA_DETALHADA"**.

A busca nas fontes de dados ocorreu em agosto de 2022 e os estudos encontrados foram exportados, organizados e armazenados com o auxílio do programa *Microsoft Excel®* e do gerenciador bibliográfico *EndNote® Online*. Ainda, esta revisão utilizou de forma adaptada o modelo *Preferred Reporting Items for Systematic Reviews and Meta-Analyses* (PRISMA) *Flow Diagram*, a fim de organizar os estudos em três etapas, incluindo identificação, triagem e inclusão.

Na primeira seleção, foram encontrados um total de 493 artigos. Deste total, foram excluídas 198 duplicatas, restando 295 artigos para leitura dos títulos, resumos e descritores. Posteriormente à leitura, foram excluídos mais 233 artigos por não atenderem aos critérios de inclusão. Ao final deste processo, foram selecionados 62 artigos como elegíveis, os quais foram lidos na íntegra. Por fim, foram excluídos mais 46 artigos por não responderem ao tema da pesquisa, sendo selecionados 16 artigos para análise. Fluxograma detalhado no **arquivo "FLUXOGRAMA_PRISMA"**.

<u>Descrição dos métodos usados para o processamento dos dados:</u>

Elaborou-se um quadro para a apresentação dos resultados, como forma de sintetizar as informações e compilar as principais evidências, contendo os seguintes tópicos: referência, ano/país, base de dados, objetivo principal e tipo de estudo. Dados detalhados no **arquivo "SINTESE_DOS_ARTIGOS_SELECIONADOS"**.

Para a avaliação e análise crítica dos estudos incluídos nesta revisão, procurou-se explicações para os dados obtidos, utilizando-se questionamentos, como: qual o objetivo do estudo? Quem eram os participantes e em que ambiente estavam inseridos? O estudo atingiu seus objetivos? Qual a contribuição do estudo acerca da telenfermagem?

Os dados obtidos foram interpretados com foco nas suas principais contribuições envoltas à temática da revisão, possibilitando assim a síntese dos achados e a categorização dos dados, momento em que estes foram divididos em três categorias temáticas.

Para a análise dos dados, realizou-se a leitura dos artigos em um processo dinâmico e intermitente, permitindo assim a síntese e a apresentação dos dados de forma organizada e coerente em relação aos objetivos propostos para esta revisão. Desta forma, para a síntese dos resultados, foram elaborados três quadros, evidenciando em seu teor os

principais resultados pertinentes às respectivas temáticas emergidas referentes aos estudos analisados.

Informações específicas dos dados:

Não possui.
