# Supplementary material for: Telenursing contributions in Primary Health Care in the COVID-19 pandemic context: an integrative review
Source: Rev Bras Enferm. 2024 Nov 22;77(5):e20240093. doi: 10.1590/0034-7167-2024-0093 (PMC11653881; doi:10.1590/0034-7167-2024-0093)
Supplement: Supplementary file 4 [file 0034-7167-reben-77-05-e20240093-suppl04.pdf]

Síntese dos 16 artigos selecionados para a Revisão Integrativa da Literatura, intitulada:

"Contribuições da telenfermagem na atenção primária à saúde no contexto pandêmico da covid-19: revisão integrativa"

Nº	Referência	País / Ano	Objetivo	Delineamento metodológico	Caracterização dos participantes	Contribuições da teleconsulta para a assistência em enfermagem
E1	RODRIGUES, *et al*. (2021)	Brasil 2021	Identificar as intervenções de enfermagem realizadas por teleconsulta ao idoso e seu cuidador no Serviço de Atenção Domiciliar (SAD) na pandemia da Covid-19.	Estudo qualitativo e quantitativo do tipo transversal.	- 140 pacientes idosos<br>Idade: 65 a 110 anos<br>Gênero: 90 mulheres e 50 homens<br>- 106 cuidadores<br>Idade e gênero não informados.	A telenfermagem demonstrou ser uma medida que atende a continuidade do cuidado dado a necessidade de isolamento social durante a pandemia da Covid-19. Recomenda-se a adoção de um instrumento de teleconsulta de enfermagem que contenham os diagnósticos e as intervenções.
E2	CHRISTINELLI, *et al*. (2021)	Brasil 2021	Analisar os efeitos de uma intervenção remota multiprofissional e da telenfermagem no tratamento da obesidade.	Estudo quantitativo do tipo ensaio clínico pragmático de intervenção.	- 22 pacientes adultas<br>Idade: média de 39 anos<br>Gênero: 22 mulheres	Os efeitos da intervenção remota multiprofissional e da telenfermagem diminuíram significativamente as variáveis de risco para a síndrome metabólica no tratamento da obesidade. Ainda, a telenfermagem possibilita um cuidado integral e de qualidade, proporcionando a diminuição na demanda de atendimento nos serviços de saúde.
E3	BROWN; HEWNER (2022)	EUA 2022	Avaliar o impacto da implementação de um programa de telessaúde gerenciado por enfermeiros por meio da telessaúde, antes e durante a pandemia.	Estudo quantitativo do tipo observacional.	- 12.299 pacientes adultos<br>Idade: 18 a 64 anos<br>Gênero: mulheres e homens (quantidade não informada).	A análise de tendências demonstra como o gerenciamento remoto de cuidados de enfermagem na pandemia da Covid-19 reduziram a utilização do departamento de emergência em populações adultas com altas necessidades sociais.
E4	CHIANG *et al*. (2021)	EUA 2021	Investigar a experiência do paciente com a telemedicina no tratamento da cefaleia durante a pandemia da Covid-19.	Estudo quantitativo.	- 1.160 pacientes adultos<br>Idade: média de 49,5 anos<br>Gênero: 1017 mulheres e 138 homens	O estudo demonstrou que a telemedicina facilitou o tratamento da dor de cabeça para muitos pacientes durante a pandemia de Covid-19.
E5	DANHIEUX *et al*. (2020)	Bélgica 2020	Examinar como o conteúdo e a prestação de cuidados crônicos estão sendo afetados pela pandemia.	Estudo qualitativo.	- 21 profissionais, entre Nutricionistas, Enfermeiras e Médicos<br>Idade: não informado<br>Gênero: 14 mulheres e 6 homens	A telenfermagem proporcionou às práticas da APS uma ferramenta potencial para monitorar e apoiar pacientes com doenças crônicas à distância.

E6	FRANZOSA *et al*. (2021)	EUA 2021	Determinar as estratégias usadas pelas práticas de atenção primária domiciliar da cidade de Nova York.	Estudo qualitativo do tipo transversal.	- 13 profissionais, entre Enfermeiras, Médicos, Gerenciador de Programa e Assistentes Sociais<br>Idade: não informado<br>Gênero: 6 mulheres e 7 homens	A telessaúde foi considerada valiosa. As visitas por vídeo podem ser desafiadoras devido às limitações físicas e cognitivas dos pacientes, sendo necessário adaptações, procurando manter a confiança entre a equipe e os pacientes.
E7	GILKEY *et al*. (2021)	EUA 2021	Caracterizar a experiência recente dos profissionais da APS usando a telessaúde à adolescentes, bem como seu apoio a ela após o término da pandemia de COVID-19.	Estudo quantitativo.	- 1.047 profissionais, entre Enfermeiras e Médicos<br>Idade: não informado<br>Gênero: 515 mulheres, 492 homens e 40 outros (não especificado)	As vantagens mais comuns da telessaúde foram prevenir a exposição à Covid-19, deixar as famílias à vontade e reduzir o transporte, tempo e os encargos, além de poder obter informações sobre os ambientes domésticos das famílias. A telessaúde aumentou e melhorou o acesso ao atendimento para os adolescentes.
E8	MARRERO *et al*. (2021)	Espanha 2021	Descrever o conceito de videoconsulta, a sua utilização na APS, o papel do enfermeiro na sua utilização, bem como a experiência de implementação.	Estudo qualitativo do tipo descritivo.	- 324 profissionais, entre: Enfermeiras, Fisioterapeutas, Parteiras e Médicos<br>Idade: não informado<br>Gênero: mulheres e homens (quantidade não informada)	A videoconsulta apresenta-se como uma forma emergente de interagir com os pacientes no primeiro nível de cuidados à saúde que, uma vez que permite aos pacientes e profissionais poupar tempo e recursos, atingindo elevados níveis de resolução de problemas.
E9	RAND (2022)	EUA 2022	Determinar se as intervenções clínicas e tecnológicas da telessaúde domiciliar, recentemente desenvolvidas, podem melhorar as taxas de vacinação entre os veteranos.	Estudo quantitativo do tipo coorte concorrente.	- 513 pacientes adultos<br>Idade: 40 a 99 anos<br>Gênero: 498 homens e 15 mulheres	Intervenções de enfermagem podem aumentar as medidas de qualidade da vacinação contra influenza para pacientes de telessaúde de cuidados primários. Ainda, este estudo demonstra o efeito positivo da telessaúde nas taxas de vacinação sazonal. A telessaúde tem potencial de se envolver na divulgação da vacinação.
E10	SQUERI *et al*. (2022)	EUA 2022	Avaliar as atitudes dos médicos em relação às mudanças na triagem telefônica de enfermagem.	Estudo qualitativo.	- 15 Médicos Assistentes<br>Idade: não informado<br>Gênero: 15 homens	As melhorias na triagem por telefone da enfermeira eram vistas pelos médicos como benéficas, valiosas e aprimoradas no atendimento ao paciente. Ainda, a assistência remota, melhorou a pontualidade do atendimento, o autogerenciamento e reduziram a procura por serviços de emergência.

E11	CELUPPI *et al*. (2022)	Brasil 2022	Compreender as práticas de gestão no cuidado às pessoas que vivem com o HIV na APS de uma capital brasileira, em tempos de pandemia da Covid-19.	Estudo qualitativo do tipo exploratório.	- 12 Enfermeiros<br>Idade e gênero não informados.	Destacou-se a assistência remota, como estratégia para ampliação do acesso. A implementação da teleconsulta na APS permitiu garantir acesso aos usuários que necessitavam dos serviços de saúde. Podendo ser considerada uma importante ferramenta de organização assistencial para o enfrentamento da pandemia.
E12	JAMES *et al*. (2021)	Austrália 2021	Explorar as experiências de enfermeiros de cuidados primários de saúde australianos no uso de telessaúde durante a Covid-19.	Estudo qualitativo.	- 25 Enfermeiros<br>Idade: não informado<br>Gênero: 24 mulheres e 1 homem	A telessaúde foi considerada como uma iniciativa positiva que melhorou o acesso dos pacientes aos cuidados. Também foi descrita como uma forma de evitar visitas domiciliares ou pacientes de alto risco nas clínicas. Destacou-se a melhoria da acessibilidade em termos de financiamento de serviços de telessaúde durante a pandemia.
E13	JOHNSON *et al*. (2021)	Canadá 2021	Avaliar as mudanças no uso da tecnologia de telessaúde na APS e outras práticas organizacionais durante a pandemia de Covid-19.	Estudo quantitativo do tipo comparativo.	- 114 profissionais, entre:<br>Enfermeiros e Médicos<br>Idade e gênero não informados.	A redução das faltas às consultas foi um benefício comum relatado pelos participantes com o aumento do uso de tecnologias de telessaúde.
E14	LAPÃO *et al*. (2021)	Portugal 2021	Apoiar a prestação de cuidados de saúde primários, com uma plataforma de saúde digital.	Estudo qualitativo do tipo prospecção tecnológica.	- 53 profissionais, entre<br>Enfermeiros e Médicos<br>Idade e gênero: não informado<br>- Pacientes idosos (quantidade não informada)<br>Idade: >60 anos<br>Gênero: não informado	Ter uma plataforma digital para apoiar os cuidados de saúde primários durante a pandemia da Covid-19, facilitou as consultas online entre as equipes e seus os seus pacientes, garantindo cuidados adequados, promovendo a adesão ao tratamento e fornecendo aconselhamento e apoio psicológico.
E15	MURPHY *et al*. (2021)	Reino Unido 2021	Investigar a rápida implementação da consultoria remota e explorar o impacto nos meses iniciais da pandemia da Covid-19.	Estudo de método misto.	- 41 profissionais, entre<br>Enfermeiros, Médicos e Gerentes de Saúde<br>Idade e gênero: não informado<br>- 350.966 pacientes<br>Idade: 0 a 85 anos<br>Gênero: 175.952 homens, 175.009 mulheres e 5 outros (não especificado)	Os enfermeiros usaram videoconsultas para treinar pacientes e/ou cuidadores sobre cuidados com feridas ou administração de medicamentos.<br>A videoconsulta mostrou-se útil para avaliação dinâmica, como marcha e monitorização respiratória, e foi útil com crianças, para avaliá-las visualmente e tranquilizar os pais.<br>As enfermeiras descobriram que a videoconsulta funcionou bem para revisões de condições crônicas. A consultoria por telefone deu aos profissionais maior controle de seu dia de trabalho.

E16	RUSSEL *et al*. (2022)	Inglaterra 2022	Explorar as experiências e percepções dos enfermeiros da atenção primária e dos assistentes de saúde sobre a clínica geral e as mudanças feitas a ela durante a pandemia.	Estudo qualitativo do tipo exploratório.	- 24 profissionais, entre Enfermeiros, Assistentes de Saúde e Auxiliar de Enfermagem<br>Idade: 20 a 69 anos<br>Gênero: 22 mulheres e 2 homens	A implementação criteriosa da telessaúde pode ajudar a preservar a natureza prática e cuidadosa da enfermagem. Prescrições de rotina, revisões de medicamentos, elementos não práticos de doenças crônicas e consultas de rotina puderam ser gerenciados de forma eficaz usando a telessaúde. Ainda, a teleconsulta forneceu flexibilidade para pacientes.
