# Supplementary material for: Telenursing contributions in Primary Health Care in the COVID-19 pandemic context: an integrative review
Source: Rev Bras Enferm. 2024 Nov 22;77(5):e20240093. doi: 10.1590/0034-7167-2024-0093 (PMC11653881; doi:10.1590/0034-7167-2024-0093)
Supplement: Translation

# Translation: Contribuições da telenfermagem na Atenção Primária à Saúde no contexto pandêmico da COVID-19: revisão integrativa

**DOI:** 10.1590/0034-7167-2024-0093pt

**Authors:** Patrícia Amidianski, Evangelia Kotzias Atherino dos Santos, Alacoque Lorenzini Erdmann, Carmem Regina Delziovo, Maria Solange Ferreira Alves, Marli Terezinha Stein Backes

**Affiliations:** I Universidade Federal de Santa Catarina. Florianópolis, Santa Catarina, Brasil; II Secretaria de Estado da Saúde de Santa Catarina. Florianópolis, Santa Catarina, Brasil

**Keywords:** Telenfermagem, Consulta Remota, Enfermagem, Atenção Primária à Saúde, COVID-19

## Abstract

**Objetivo:**

identificar as contribuições da telenfermagem na Atenção Primária à Saúde no contexto pandêmico da COVID-19.

**Métodos:**

revisão integrativa da literatura, conduzida entre janeiro e agosto de 2022 nas bases de dados PubMed, CINAHL, LILACS, BDENF, Scopus, WoS, EMBASE e SciELO. Foram encontrados 493 estudos no total, 62 lidos na íntegra, e, desses, 16 foram selecionados. Para análise, realizou-se uma leitura dinâmica dos estudos e síntese dos principais resultados.

**Resultados:**

os principais resultados evidenciaram a prática da telenfermagem como um desafio para os profissionais e para a população. Entre as contribuições e os pontos positivos e aspectos que necessitam de aprimoramento, a prática mostrou-se promissora ao ser pressuposta em um cenário pós-pandêmico.

**Considerações finais:**

por meio da telenfermagem, foi garantido o acesso da população à Atenção Primária à Saúde diante da pandemia de COVID-19. No entanto, faz-se necessário um olhar crítico para os atuais avanços tecnológicos na área da saúde.

## INTRODUÇÃO

A prática da telessaúde vem sendo encorajada pela Organização Mundial da Saúde (OMS) desde 2005 como meio estratégico de melhoria para os sistemas de saúde, entre eles o Sistema Único de Saúde (SUS)^(1)^. Em âmbito mundial, torna-se importante destacar a atual transformação digital na área da saúde, uma vez que a “Estratégia Global de Saúde Digital 2020-2025”, proposta pela OMS, surge para fortalecer os sistemas de saúde por meio da utilização de tecnologias digitais, fortalecendo, assim, o conceito de saúde universal^(2)^. Já no Brasil, o Ministério da Saúde desenvolveu a “Estratégia de Saúde Digital para o Brasil 2020-2028”, que busca atualizar a Política Nacional de Informação e Informática em Saúde (PNIIS), além de reafirmar políticas, diretrizes, portarias, ações e iniciativas no âmbito da saúde digital no SUS^(3)^.

Partindo da evolução tecnológica em relação à saúde, é preciso destacar o advento da pandemia de COVID-19, a qual acelerou consideravelmente a evolução da saúde digital. Declarada em março de 2020 pela OMS, a pandemia de COVID-19 veio acompanhada de inúmeras restrições como forma de combate à disseminação do vírus SARS-CoV-2 entre a população, implementando medidas, como distanciamento e isolamento social. Com o rápido avanço da pandemia, os sistemas de saúde entraram em colapso e foram necessárias estratégias emergenciais para assegurar o acesso da população aos serviços de saúde^(4)^.

A partir disso, a telenfermagem foi amplamente incentivada e praticada em escala mundial como forma de lidar com a alta demanda nos serviços de saúde. Trata-se de uma modalidade assistencial em formato eletrônico/digital que possibilita realizar consultas de enfermagem, monitoramentos, consultorias, interconsultas, ações de educação em saúde e acolhimento, mediadas pelas Tecnologias da Informação e Comunicação (TICs)^(5)^.

No Brasil, entre as diversas áreas da saúde, a enfermagem ganhou respaldo ético e legal por meio da Resolução COFEN (Conselho Federal de Enfermagem) nº 634/2020 para exercer a teleconsulta de enfermagem, a qual foi autorizada e normatizada como estratégia de combate à pandemia de COVID-19, incluindo o uso das TICs às competências da profissão^(6)^. Devido à permanência da pandemia de COVID-19, no ano de 2022, o COFEN normatizou a telenfermagem por meio da Resolução COFEN nº 696/2022, alterada pela Resolução COFEN nº 707/2022^(7)^.

Nesta conjuntura de acontecimentos, torna-se importante destacar o papel da enfermagem no contexto da APS, considerada a porta de entrada para o SUS. A enfermagem ganhou destaque, principalmente, com o surgimento da pandemia e com a necessidade de se reinventar no ambiente e nos fluxos de trabalho de forma digital. As orientações em saúde, o acolhimento qualificado e as consultas em formato digital tornaram-se práticas valiosas para o combate à pandemia, além de ser uma importante estratégia para a continuidade da assistência à saúde^(8)^.

## OBJETIVO

Identificar as contribuições da telenfermagem na APS no contexto pandêmico da COVID-19.

## MÉTODOS

### Aspectos éticos

Este estudo foi realizado em bases de domínio público, o que dispensa a submissão a um Comitê de Ética em Pesquisa.

### Referencial metodológico

Utilizou-se o referencial metodológico de Mendes, Silveira e Galvão^(9)^, os quais consideram a revisão de literatura uma metodologia capaz de dar suporte científico para tomadas de decisão e práticas clínicas na área da saúde.

### Tipo de estudo

Trata-se de revisão integrativa da literatura que buscou identificar, analisar e sintetizar resultados de evidências científicas, a fim de gerar conhecimento em relação à temática e possibilitar novas contribuições para a melhoria da prática clínica^(9)^.

### Procedimentos metodológicos

Para este estudo, trilharam-se as seis etapas metodológicas propostas por Mendes, Silveira e Galvão^(9)^, a saber: formulação da questão de pesquisa; estabelecimento de critérios para inclusão e exclusão de estudos; extração dos dados dos estudos; avaliação e análise crítica dos estudos a serem incluídos na revisão; interpretação dos resultados; e apresentação da revisão/síntese do conhecimento.

Primeiramente, formulou-se a seguinte questão norteadora: quais as contribuições da telenfermagem na APS no contexto pandêmico da COVID-19? Para a formulação adequada, utilizou-se o acrônimo PICO^(9)^ da seguinte forma: P (População) = população em geral; I (tópico de Interesse) = telenfermagem na APS no cenário pandêmico da COVID-19; C (Comparação) = não se aplica; O (*Outcome*/Desfecho) = contribuições da prática.

Para os critérios de inclusão, consideraram-se artigos científicos primários de abordagem qualitativa e quantitativa, publicados no período de janeiro de 2020 a agosto de 2022, nos idiomas português, inglês e espanhol, que respondessem à questão de pesquisa. Já para os critérios de exclusão, foram considerados estudos na modalidade de revisões, editoriais, cartas, artigos de opinião, comentários, resumos de anais, publicações duplicadas, dossiês, trabalhos de conclusão de curso, documentos oficiais de programas nacionais e internacionais, relatos de experiência, estudos de reflexão, estudos teóricos, teses, dissertações, boletins epidemiológicos, relatórios de gestão e livros.

### Fonte de dados

Em relação às fontes de dados, foram incluídas as bases de dados científicas *Public Medical Literature Analysis and Retrieval System Online* (PubMed), *Cumulative Index to Nursing & Allied Helth Literature* (CINAHL), Literatura Latino-Americana e do Caribe em Ciências da Saúde (LILACS), Base de Dados de Enfermagem (BDENF), Scopus, *Web of Science* (WoS) e EMBASE, bem como a biblioteca virtual de revistas científicas *Scientific Electronic Library Online* (SciELO). Ainda, a pesquisa contou com o auxílio do Portal de Periódicos da Coordenação de Aperfeiçoamento de Pessoal de Nível Superior (CAPES) e da Biblioteca Virtual em Saúde (BVS).

Os termos utilizados para a pesquisa foram selecionados por meio dos Descritores em Ciências da Saúde (DeCS) e do *Medical Subject Headings* (Mesh), a saber: Consulta Remota, Telemedicina, Telenfermagem (*Teleenfermería, Remote Consultation, Telemedicine, Telenursing*); Enfermagem (*Enfermeria, Nursing, Nurses*); Atenção Primária à Saúde (*Atención Primaria de Salud, Primary Health Care, Enfermagem de Atenção Primária, Enfermería de Atención Primaria, Primary Care Nursing, Enfermagem Primária, Enfermería Primaria, Primary Nursing*); e COVID-19. Para a combinação dos descritores, utilizaram-se os operadores booleanos *AND* e *OR*.

**Quadro 1:** Estratégias de busca para as bases de dados incluídas na revisão, 2022

Bases de dados	Estratégias de busca
PubMed	((“Remote Consultation”[Mesh] OR “Remote Consultation” OR “Teleconsultation” OR “Asynchronous Teleconsultation” OR “Synchronous Teleconsultation” OR “Telemedicine”[Mesh] OR “Telemedicine” OR “Telenursing”[Mesh] OR “Telenursing” OR “Telecare” OR “Telecure” OR “Telehealth” OR Tele^*^[Title/Abstract]) AND (“Nursing”[Mesh] OR “Nursing”[Title/Abstract] OR “Nurse”[Title/Abstract] OR “Nurses”[Mesh] OR “Nurses”[Title/Abstract] OR Nurs^*^[Title/Abstract]) AND (“Primary Health Care”[Mesh] OR “Primary Health Care” OR “Primary Healthcare” OR “Primary Care” OR “Basic Health Care” OR “Basic Care” OR “Basic Service” OR “Primary Care Nursing”[Mesh] OR “Primary Care Nursing” OR “Primary Nursing”[Mesh] OR “Primary Nursing”) AND (“Coronavirus Infections”[Mesh] OR “Coronavirus Infections” OR “Coronavirus”[Mesh] OR “Coronavirus” OR “SARS Virus”[Mesh] OR “SARS Virus” OR “SARS-CoV” OR “COVID-19”[Mesh] OR “COVID-19” OR “SARS-CoV-2”[Mesh] OR “SARS-CoV-2” OR “SARSCoV2” OR “SARS2” OR “COVID19” OR “COVID-2019” OR “COVID 2019” OR “SARS COV 2” OR “2019-nCoV” OR “2019ncov” OR “nCoV 2019”))
CINAHL	(((MH “Remote Consultation”+) OR “Remote Consultation” OR “Teleconsultation” OR “Asynchronous Teleconsultation” OR “Synchronous Teleconsultation” OR (MH Telemedicine+) OR “Telemedicine” OR (MH “Telenursing”+) OR “Telenursing” OR “Telecare” OR “Telecure” OR “Telehealth” OR (TI Tele^*^ OR AB Tele^*^)) AND ((MH “Nursing”+) OR (TI “Nursing” OR AB “Nursing”) OR (TI “Nurse” OR AB “Nurse”) OR (MH “Nurses”+) OR (TI “Nurses” OR AB “Nurses”) OR (TI Nurs^*^ OR AB Nurs^*^)) AND ((MH “Primary Health Care”+) OR “Primary Health Care” OR “Primary Healthcare” OR “Primary Care” OR “Basic Health Care” OR “Basic Care” OR “Basic Service” OR (MH “Primary Care Nursing”+) OR “Primary Care Nursing” OR (MH “Primary Nursing”+) OR “Primary Nursing” ) AND ((MH “Coronavirus Infections”+) OR “Coronavirus Infections” OR (MH “Coronavirus”+) OR “Coronavirus” OR (MH “SARS Virus”+) OR “SARS Virus” OR “SARS-CoV” OR (MH “COVID-19”+) OR “COVID-19” OR (MH “SARS-CoV-2”+) OR “SARS-CoV-2” OR “SARSCoV2” OR “SARS2” OR “COVID19” OR “COVID-2019” OR “COVID 2019” OR “SARS COV 2” OR “2019-nCoV” OR “2019ncov” OR “nCoV 2019” ))
LILACS	((“Consulta Remota” OR “Consulta à Distância” OR “Teleconsulta” OR “Teleconsultoria” OR “Telemedicina” OR “Saúde Digital” OR “e-Saúde” OR “Telessaúde” OR “Teleassistência” OR “Telenfermagem” OR “Teleenfermería” OR “Telesalud” OR “Teleasistencia” OR “eSalud” OR “Telecuidado” OR “Remote Consultation” OR “Remote Consultation” OR “Teleconsultation” OR “Asynchronous Teleconsultation” OR “Synchronous Teleconsultation” OR “Telemedicine” OR “Telemedicine” OR “Telenursing” OR “Telenursing” OR “Telecare” OR “Telecure” OR “Telehealth” OR Tele^*^) AND (“Enfermagem” Enfermeir^*^ OR “Enfermeria” OR Enfermer^*^ OR “Nursing” OR “Nurse” OR “Nurses” OR Nurs^*^) AND (“Atenção Primária à Saúde” OR “Atenção Básica” OR “Atenção Primária” OR “Atendimento Básico” OR “Atendimento Primário” OR “Cuidado de Saúde Primário” OR “Cuidado Primário” OR “Cuidado de Saúde Básico” OR “Cuidado Básico” OR “Enfermagem de Atenção Primária” OR “Enfermagem Primária” OR “Enfermaria Primária” OR “Atención Primaria de Salud” OR “Atención Primaria” OR “Atención Básica” OR “Cuidado de la Salud Primarios” OR “Servicio Básico” OR “Servicios Básicos” OR “Enfermería de Atención Primaria” OR “Enfermería Primaria” OR “Primary Health Care” OR “Primary Healthcare” OR “Primary Care” OR “Basic Health Care” OR “Basic Care” OR “Basic Service” OR “Primary Care Nursing” OR “Primary Nursing”) AND (“Infecções por Coronavirus” OR “Vírus da SARS” OR “Infecciones por Coronavirus” OR “Virus del SRAS” OR “Coronavirus Infections” OR “Coronavirus” OR “SARS Virus” OR “SARS-CoV” OR “COVID-19” OR “SARS-CoV-2” OR “SARSCoV2” OR “SARS2” OR “COVID19” OR “COVID-2019” OR “COVID 2019” OR “SARS COV 2” OR “2019-nCoV” OR “2019ncov” OR “nCoV 2019”))
BDENF	((“Consulta Remota” OR “Consulta à Distância” OR “Teleconsulta” OR “Teleconsultoria” OR “Telemedicina” OR “Saúde Digital” OR “e-Saúde” OR “Telessaúde” OR “Teleassistência” OR “Telenfermagem” OR “Teleenfermería” OR “Telesalud” OR “Teleasistencia” OR “eSalud” OR “Telecuidado” OR “Remote Consultation” OR “Remote Consultation” OR “Teleconsultation” OR “Asynchronous Teleconsultation” OR “Synchronous Teleconsultation” OR “Telemedicine” OR “Telemedicine” OR “Telenursing” OR “Telenursing” OR “Telecare” OR “Telecure” OR “Telehealth” OR Tele^*^) AND (“Enfermagem” Enfermeir^*^ OR “Enfermeria” OR Enfermer^*^ OR “Nursing” OR “Nurse” OR “Nurses” OR Nurs^*^) AND (“Atenção Primária à Saúde” OR “Atenção Básica” OR “Atenção Primária” OR “Atendimento Básico” OR “Atendimento Primário” OR “Cuidado de Saúde Primário” OR “Cuidado Primário” OR “Cuidado de Saúde Básico” OR “Cuidado Básico” OR “Enfermagem de Atenção Primária” OR “Enfermagem Primária” OR “Enfermaria Primária” OR “Atención Primaria de Salud” OR “Atención Primaria” OR “Atención Básica” OR “Cuidado de la Salud Primarios” OR “Servicio Básico” OR “Servicios Básicos” OR “Enfermería de Atención Primaria” OR “Enfermería Primaria” OR “Primary Health Care” OR “Primary Healthcare” OR “Primary Care” OR “Basic Health Care” OR “Basic Care” OR “Basic Service” OR “Primary Care Nursing” OR “Primary Nursing”) AND (“Infecções por Coronavirus” OR “Vírus da SARS” OR “Infecciones por Coronavirus” OR “Virus del SRAS” OR “Coronavirus Infections” OR “Coronavirus” OR “SARS Virus” OR “SARS-CoV” OR “COVID-19” OR “SARS-CoV-2” OR “SARSCoV2” OR “SARS2” OR “COVID19” OR “COVID-2019” OR “COVID 2019” OR “SARS COV 2” OR “2019-nCoV” OR “2019ncov” OR “nCoV 2019”))
SciELO	((“Consulta Remota” OR “Consulta à Distância” OR “Teleconsulta” OR “Teleconsultoria” OR “Telemedicina” OR “Saúde Digital” OR “e-Saúde” OR “Telessaúde” OR “Teleassistência” OR “Telenfermagem” OR “Teleenfermería” OR “Telesalud” OR “Teleasistencia” OR “eSalud” OR “Telecuidado” OR “Remote Consultation” OR “Remote Consultation” OR “Teleconsultation” OR “Asynchronous Teleconsultation” OR “Synchronous Teleconsultation” OR “Telemedicine” OR “Telemedicine” OR “Telenursing” OR “Telenursing” OR “Telecare” OR “Telecure” OR “Telehealth” OR Tele^*^) AND (“Enfermagem” Enfermeir^*^ OR “Enfermeria” OR Enfermer^*^ OR “Nursing” OR “Nurse” OR “Nurses” OR Nurs^*^) AND (“Atenção Primária à Saúde” OR “Atenção Básica” OR “Atenção Primária” OR “Atendimento Básico” OR “Atendimento Primário” OR “Cuidado de Saúde Primário” OR “Cuidado Primário” OR “Cuidado de Saúde Básico” OR “Cuidado Básico” OR “Enfermagem de Atenção Primária” OR “Enfermagem Primária” OR “Enfermaria Primária” OR “Atención Primaria de Salud” OR “Atención Primaria” OR “Atención Básica” OR “Cuidado de la Salud Primarios” OR “Servicio Básico” OR “Servicios Básicos” OR “Enfermería de Atención Primaria” OR “Enfermería Primaria” OR “Primary Health Care” OR “Primary Healthcare” OR “Primary Care” OR “Basic Health Care” OR “Basic Care” OR “Basic Service” OR “Primary Care Nursing” OR “Primary Nursing”) AND (“Infecções por Coronavirus” OR “Vírus da SARS” OR “Infecciones por Coronavirus” OR “Virus del SRAS” OR “Coronavirus Infections” OR “Coronavirus” OR “SARS Virus” OR “SARS-CoV” OR “COVID-19” OR “SARS-CoV-2” OR “SARSCoV2” OR “SARS2” OR “COVID19” OR “COVID-2019” OR “COVID 2019” OR “SARS COV 2” OR “2019-nCoV” OR “2019ncov” OR “nCoV 2019”))
Scopus	((“Remote Consultation” OR “Teleconsultation” OR “Asynchronous Teleconsultation” OR “Synchronous Teleconsultation” OR “Telemedicine” OR “Telenursing” OR “Telecare” OR “Telecure” OR “Telehealth” OR Tele^*^) AND (“Nursing” OR “Nurse” OR “Nurses”[Mesh] OR “Nurses” OR Nurs^*^) AND (“Primary Health Care” OR “Primary Healthcare” OR “Primary Care” OR “Basic Health Care” OR “Basic Care” OR “Basic Service” OR “Primary Care Nursing” OR “Primary Nursing”) AND (“Coronavirus Infections” OR “Coronavirus” OR “SARS Virus” OR “SARS-CoV” OR “COVID-19” OR “SARS-CoV-2” OR “SARSCoV2” OR “SARS2” OR “COVID19” OR “COVID-2019” OR “COVID 2019” OR “SARS COV 2” OR “2019-nCoV” OR “2019ncov” OR “nCoV 2019”))
WoS	((“Remote Consultation” OR “Teleconsultation” OR “Asynchronous Teleconsultation” OR “Synchronous Teleconsultation” OR “Telemedicine” OR “Telenursing” OR “Telecare” OR “Telecure” OR “Telehealth” OR Tele^*^) AND (“Nursing” OR “Nurse” OR “Nurses”[Mesh] OR “Nurses” OR Nurs^*^) AND (“Primary Health Care” OR “Primary Healthcare” OR “Primary Care” OR “Basic Health Care” OR “Basic Care” OR “Basic Service” OR “Primary Care Nursing” OR “Primary Nursing”) AND (“Coronavirus Infections” OR “Coronavirus” OR “SARS Virus” OR “SARS-CoV” OR “COVID-19” OR “SARS-CoV-2” OR “SARSCoV2” OR “SARS2” OR “COVID19” OR “COVID-2019” OR “COVID 2019” OR “SARS COV 2” OR “2019-nCoV” OR “2019ncov” OR “nCoV 2019”))
EMBASE	((‘Remote Consultation’/exp OR ‘Remote Consultation’ OR ‘Teleconsultation’ OR ‘Asynchronous Teleconsultation’ OR ‘Synchronous Teleconsultation’ OR ‘Telemedicine’/exp OR ‘Telemedicine’ OR ‘Telenursing’/exp OR ‘Telenursing’ OR ‘Telecare’ OR ‘Telecure’ OR ‘Telehealth’ OR Tele^*^:ti,ab) AND (‘Nursing’/exp OR ‘Nursing’:ti,ab OR ‘Nurse’:ti,ab OR ‘Nurses’/exp OR ‘Nurses’:ti,ab OR Nurs^*^:ti,ab) AND (‘Primary Health Care’/exp OR ‘Primary Health Care’ OR ‘Primary Healthcare’ OR ‘Primary Care’ OR ‘Basic Health Care’ OR ‘Basic Care’ OR ‘Basic Service’ OR ‘Primary Care Nursing’/exp OR ‘Primary Care Nursing’ OR ‘Primary Nursing’/exp OR ‘Primary Nursing’) AND (‘Coronavirus Infections’/exp OR ‘Coronavirus Infections’ OR ‘Coronavirus’/exp OR ‘Coronavirus’ OR ‘SARS Virus’/exp OR ‘SARS Virus’ OR ‘SARS-CoV’ OR ‘COVID-19’/exp OR ‘COVID-19’ OR ‘SARS-CoV-2’/exp OR ‘SARS-CoV-2’ OR ‘SARSCoV2’ OR ‘SARS2’ OR ‘COVID19’ OR ‘COVID-2019’ OR ‘COVID 2019’ OR ‘SARS COV 2’ OR ‘2019-nCoV’ OR ‘2019ncov’ OR ‘nCoV 2019’))

### Coleta e organização dos dados

A coleta de dados ocorreu em agosto de 2022, e os estudos encontrados foram exportados, organizados e armazenados com o auxílio do *software Microsoft Excel* ^®^. Ainda, esta revisão utilizou de forma adaptada o modelo *Preferred Reporting Items for Systematic Reviews and Meta-Analyses* (PRISMA) *Flow Diagram* ^(10)^.

Na primeira seleção, foram encontrados 493 artigos. Desse total, foram excluídas 198 duplicatas, restando 295 artigos para leitura dos títulos, resumos e descritores. Posteriormente à leitura, foram excluídos mais 233 artigos, por não atenderem aos critérios de inclusão. Ao final desse processo, foram selecionados 62 artigos como elegíveis, os quais foram lidos na íntegra. Por fim, foram excluídos mais 46 artigos por não responderem ao tema da pesquisa, sendo selecionados 16 artigos para análise (Figura 1).

**Figura 1:** Fluxograma das etapas de seleção dos estudos ancorado no PRISMA, 2022

Doravante, na terceira etapa, houve a extração dos dados dos estudos selecionados, que foram sintetizados e serão apresentados no quadro sinóptico ao decorrer desta revisão. Já na quarta etapa, para a avaliação e análise crítica dos estudos incluídos nesta revisão, procuraram-se explicações para os dados obtidos, utilizando-se questionamentos, como: qual o objetivo do estudo? Quem eram os participantes e em que ambiente estavam inseridos? O estudo atingiu seus objetivos? Quais os principais resultados? Quais as contribuições da telenfermagem?

Na quinta etapa, os dados obtidos foram interpretados com foco nas suas principais contribuições envoltas à temática desta revisão, possibilitando, assim, a síntese dos achados e a categorização dos dados, momento em que esses foram divididos em três categorias temáticas, mencionadas posteriormente nos resultados.

Por fim, na sexta e última etapa, para a apresentação da síntese do conhecimento, os dados foram apresentados de modo descritivo, de acordo com as suas categorias, e discutidos à luz da literatura científica pertinente.

### Análise dos dados

Para a análise dos dados, realizou-se a leitura dos artigos na íntegra, permitindo, assim, a síntese e a apresentação dos dados, de forma organizada e coerente, em relação aos objetivos propostos para esta revisão. Durante esse processo, como forma de organizar os dados durante a leitura, foram elaborados dois quadros e uma figura, evidenciando em seu teor os principais resultados pertinentes às respectivas temáticas emergidas dos estudos analisados.

## RESULTADOS

### Síntese dos estudos selecionados

Os 16 artigos selecionados para esta revisão foram distribuídos nas bases de dados Scopus (4; 25%), PubMed/MEDLINE (3; 18,75%), CINAHL (3; 18,75%), WoS (2; 12,5%), EMBASE (2; 12,5%), BDENF (1; 6,25%), SciELO (1; 6,25%) e LILACS (0). Ainda, os países em que os estudos foram publicados incluíram os Estados Unidos da América (EUA) (6; 37,5%), Brasil (3; 18,75%), Bélgica (1; 6,25%), Espanha (1; 6,25%), Austrália (1; 6,25%), Canadá (1; 6,25%), Portugal (1; 6,25%), Reino Unido (1; 6,25%) e Inglaterra (1; 6,25%).

Quanto ao idioma dos estudos, a maioria foi publicada em inglês (12; 75%), três, em português (18,75%), e um, em espanhol (6,25%). Referente aos anos de publicação, um artigo foi publicado em 2020 (6,25%), dez, em 2021 (62,5%), e cinco, em 2022 (31,25%). Alusivo ao delineamento dos estudos, oito são de abordagem qualitativa (50%), seis, de abordagem quantitativa (37,5), um, de método misto (6,25%), e um dos estudou utilizou tanto a abordagem quantitativa quanto a qualitativa (6,25%).

No que diz respeito aos participantes das pesquisas, em sua totalidade, os mesmos foram divididos entre profissionais de saúde, pacientes (crianças, adultos, mulheres e idosos, com idade de 0 a 110 anos, entre homens e mulheres) e cuidadores. Desta forma, nove estudos contaram com profissionais de saúde como participantes (56,25%); quatro estudos incluíram pacientes como participantes (25%); dois estudos contaram tanto com profissionais de saúde quanto com pacientes (12,5%); e um estudo incluiu pacientes e cuidadores (6,25%).

Na totalidade dos artigos selecionados, cabe salientar que a maioria se referiu à equipe multiprofissional da APS. Em uma descrição abrangente dos participantes, os profissionais de saúde compreenderam enfermeiros, médicos, nutricionistas, coordenadores de enfermagem, diretores médicos, assistentes sociais, coordenadores do serviço social, fisioterapeutas, parteiras, médicos assistentes, auxiliares de enfermagem, gerenciadores de programa, profissionais da prática avançada e assistentes de saúde.

Ademais, elaborou-se um quadro para a apresentação dos resultados das revisões como forma de sintetizar as informações e compilar as principais evidências, organizado com quatro tópicos, a saber: título; ano/país; delineamento/número de participantes; e intervenções na prática da telenfermagem (Quadro 2).

**Quadro 2:** Descrição dos estudos incluídos na revisão integrativa, 2022

Título	Ano / país	Delineamento / número de participantes	Intervenções na prática da telenfermagem^(5)^
*Telenursing in the home care service in COVID-19 pandemic: a cross-sectional study* ^(11)^	2021 Brasil	Qualitativo e quantitativo do tipo transversal n=246	Consulta via chamada de voz em formato síncrono.
Intervenção multiprofissional e telenfermagem no tratamento de obesos na pandemia de COVID-19: ensaio clínico pragmático^(12)^	2021 Brasil	Quantitativo do tipo ensaio clínico pragmático de intervenção n=22	Interconsulta, monitoramento e ações de educação em saúde via mensagens de texto, áudio e vídeo em formato assíncrono.
*The role of telehealth and clinical informatics in data driven primary care redesign* ^(13)^	2022 EUA	Quantitativo do tipo observacional n=12.299	Consulta e interconsulta via chamada de voz em formato síncrono.
*Patient experience of telemedicine for headache care during the COVID-19 pandemic: An American Migraine Foundation survey study* ^(14)^	2021 EUA	Quantitativo do tipo descritivo-exploratório n=1.160	Consulta e interconsulta via chamada de voz em formato síncrono.
*The impact of COVID-19 on chronic care according to providers: a qualitative study among primary care practices in Belgium* ^(15)^	2020 Bélgica	Qualitativo do tipo descritivo-exploratório n=21	Consulta e interconsulta via chamada de voz em formato síncrono.
*“At home, with care”: lessons from New York City home-based primary care practices managing COVID-19* ^(16)^	2021 EUA	Qualitativo do tipo transversal n=13	Consulta, interconsulta e monitoramento via chamada de voz em formato síncrono.
*Using telehealth to deliver primary care to adolescents during and after the COVID-19 pandemic: national survey study of us primary care professionals* ^(17)^	2021 EUA	Quantitativo do tipo descritivo-exploratório n=1.047	Consulta via chamada de voz e vídeo em formato síncrono.
*La vídeo-consulta en atención primaria de salud: una experiencia de implantación* ^(18)^	2021 Espanha	Qualitativo do tipo exploratório-descritivo n=324	Consulta via chamada de voz e vídeo em formato síncrono.
*Nursing interventions increase influenza vaccination quality measures for home telehealth patients* ^(19)^	2022 EUA	Quantitativo do tipo coorte concorrente n=513	Interconsulta e monitoramento via mensagens de texto em formato assíncrono.
*Meeting the challenges of COVID-19: evaluation of nurse-led changes to telephonic assessment* ^(20)^	2022 EUA	Qualitativo do tipo exploratório-descritivo n=15	Consulta via chamada de voz e vídeo em formato síncrono.
Gestão no cuidado às pessoas com HIV na Atenção Primária à Saúde em tempos do novo coronavírus^(21)^	2022 Brasil	Qualitativo do tipo exploratóriodescritivo n=12	Consulta via chamada de voz em formato síncrono e interconsulta e monitoramento via mensagem de texto em formato assíncrono.
*Experiences of Australian primary healthcare nurses in using telehealth during COVID-19: a qualitative study* ^(22)^	2021 Austrália	Qualitativo do tipo exploratóriodescritivo n=25	Consulta via chamada de voz e vídeo em formato síncrono.
*Changes to telehealth practices in primary care in New Brunswick (Canada): a comparative study pre and during the COVID-19 pandemic* ^(23)^	2021 Canadá	Quantitativo do tipo comparativo n=114	Consulta via chamada de voz e vídeo em formato síncrono e interconsulta via mensagem de texto em formato assíncrono.
*Implementation of digital monitoring services during the COVID-19 pandemic for patients with chronic diseases: Design Science Approach* ^(24)^	2021 Portugal	Qualitativo do tipo prospecção tecnológica n=53	Consulta via chamada de voz e vídeo em formato síncrono e monitoramento via mensagem de texto em formato assíncrono.
*Implementation of remote consulting in UK primary care following the COVID-19 pandemic: a mixed-methods longitudinal study* ^(25)^	2021 Reino Unido	Quantitativo do tipo longitudinal de métodos mistos n=41	Consulta via chamada de voz em formato síncrono.
*What can general practice learn from primary care nurses’ and healthcare assistants’ experiences of the COVID-19 pandemic? A qualitative study* ^(26)^	2022 Inglaterra	Qualitativo do tipo exploratório n=24	Consulta via chamada de voz em formato síncrono.

*Nota: HIV - Human Immunodeficiency Virus/Vírus da Imunodeficiência Humana; APS - Atenção Primária à Saúde; UK - United Kingdom/Reino Unido; EUA - Estados Unidos da América.*

### Síntese dos resultados

A partir da análise dos artigos, emergiram três categorias temáticas, a saber: Contribuições da telenfermagem na Atenção Primária à Saúde; Compilado de aspectos positivos e aspectos que necessitam de aprimoramento para a prática da telenfermagem na Atenção Primária à Saúde; e Perspectivas de consolidação da telenfermagem em um cenário pós-pandêmico.

### Contribuições da telenfermagem na Atenção Primária à Saúde

Com base no objetivo principal desta revisão, na primeira categoria, destacam-se as principais evidências do total de 16 estudos analisados a respeito das contribuições da teleconsulta para a enfermagem na APS. Os estudos evidenciaram uma ampla diversidade de demandas em saúde, entre problemas agudos e crônicos. A partir disso, foram identificadas variadas contribuições da telenfermagem na APS, conforme mostra o Quadro 3.

**Quadro 3:** Evidências acerca das contribuições da telenfermagem na Atenção Primária à Saúde, 2022

Título	Demandas em saúde	Contribuições da consulta remota na APS
*Telenursing in the home care service in COVID-19 pandemic: a cross-sectional study* ^(11)^	Cuidados em geral à pessoa idosa em SAD	As principais intervenções foram relacionadas à manutenção dos cuidados de saúde, isolamento social, manutenção da saúde física e mental e higiene.
Intervenção multiprofissional e telenfermagem no tratamento de obesos na pandemia de COVID-19: ensaio clínico pragmático^(12)^	Obesidade	A intervenção remota multiprofissional e da telenfermagem diminuíram significativamente as variáveis de risco para a síndrome metabólica no tratamento da obesidade.
*The role of telehealth and clinical informatics in data driven primary care redesign* ^(13)^	DRC, DM, DPOC, cardiopatias, obesidade, asma, transtornos mentais, distúrbio lipídico e tabagismo	A implementação da telessaúde apoiou o gerenciamento de cuidados crônicos de saúde e evitou visitas a emergências ou necessidade de internação.
*Patient experience of telemedicine for headache care during the COVID-19 pandemic: An American Migraine Foundation survey study* ^(14)^	Cefaleia	A maioria dos pacientes classificou a experiência com a teleconsulta como “muito boa” (62,1%) tanto para avaliação quanto para o tratamento de cefaleia. Os demais pacientes classificaram a experiência como “boa” (20,7%), “razoável” (10,5%), “ruim” (3,6%) e “outros” (3,1%).
*The impact of COVID-19 on chronic care according to providers: a qualitative study among primary care practices in Belgium* ^(15)^	DM e doenças relacionas à COVID-19	O estudo mostra que a pandemia afetou a continuidade dos cuidados crônicos, o que elevou o potencial da assistência à saúde de forma digital e remota.
*“At home, with care”: lessons from New York City home-based primary care practices managing COVID-19* ^(16)^	Cuidados em geral à pessoa idosa em atenção domiciliar	As práticas priorizavam a comunicação aberta e honesta com os pacientes, além de equilibrar a telessaúde com a necessidade de cuidados práticos.
*Using telehealth to deliver primary care to adolescents during and after the COVID-19 pandemic: national survey study of us primary care professionals* ^(17)^	Saúde do adolescente (doenças agudas e crônicas, saúde mental e comportamental e vacinação)	A maioria dos participantes concordou que a telessaúde aumenta o acesso aos cuidados dos adolescentes (69%).
*La vídeo-consulta en atención primaria de salud: una experiencia de implantación* ^(18)^	Saúde da mulher (lesões de pele, úlceras por pressão, resultados de exames, pré-natal, avaliações puerperais e aleitamento materno	A videoconsulta é apresentada como uma forma emergente de interagir com os pacientes, economiza tempo e recursos, alcançando altos níveis de resolubilidade.
*Nursing interventions increase influenza vaccination quality measures for home telehealth patients* ^(19)^	Vacinação	As intervenções acerca do monitoramento remoto dos pacientes resultaram no aumento das taxas de vacinação, alcançando 70,4% de pacientes de 19 a 65 anos e 81,7% de pacientes com mais de 66 anos.
*Meeting the challenges of COVID-19: evaluation of nurse-led changes to telephonic assessment* ^(20)^	Triagem de sinais e sintomas da COVID-19	As triagens telefônicas foram vistas como benéficas, valiosas e melhoraram o atendimento ao paciente.
Gestão no cuidado às pessoas com HIV na Atenção Primária à Saúde em tempos do novo coronavírus^(21)^	HIV	Destacaram-se a incorporação de tecnologias de cuidado não presencial e a facilitação de rotinas como estratégias para ampliação do acesso.
*Experiences of Australian primary healthcare nurses in using telehealth during COVID-19: a qualitative study* ^(22)^	Cuidados em geral à saúde da mulher, materno-infantil, saúde mental e à população aborígine	A telessaúde forneceu um meio para continuar a prestação de serviços de APS durante a COVID-19.
*Changes to telehealth practices in primary care in New Brunswick (Canada): a comparative study pre and during the COVID-19 pandemic* ^(23)^	Cuidados em geral voltados à saúde da família	O uso de consultas telefônicas aumentou 122%, passando de 43,9%, pré-pandemia, para 97,6%, durante a pandemia. O mesmo ocorreu com a consulta virtual, que passou de 19,3% para 41,2%.
*Implementation of digital monitoring services during the COVID-19 pandemic for patients with chronic diseases: Design Science Approach* ^(24)^	População idosa diagnosticada com DPOC, cardiopatias, AVC, asma, DM, câncer ou condições reumáticas	O total de participantes conseguiu gerir remotamente os pacientes de forma segura e completa, com elevado grau de satisfação.
*Implementation of remote consulting in UK primary care following the COVID-19 pandemic: a mixed-methods longitudinal study* ^(25)^	Cuidados em geral voltados à saúde da família	A mudança para a consulta remota mostrou-se bem-sucedida para o alcance e foco de pacientes vulneráveis (idosos e pessoas com transtornos mentais).
*What can general practice learn from primary care nurses’ and healthcare assistants’ experiences of the COVID-19 pandemic? A qualitative study* ^(26)^	Cuidados em geral acerca da atenção primária	A implementação criteriosa da telessaúde pode ajudar a preservar a natureza prática e atenciosa da enfermagem.

*Nota: SAD - Serviço de Atenção Domiciliar; APS - Atenção Primária à Saúde; DRC - Doença Renal Crônica; DM - Diabete Mellitus; DPOC - Doença Pulmonar Obstrutiva Crônica; HIV - Human Immunodeficiency Virus/Vírus da Imunodeficiência Humana; AVC - Acidente Vascular Cerebral; UK - United Kingdom/Reino Unido.*

### Compilado de aspectos positivos e aspectos que necessitam de aprimoramento da prática da telenfermagem na Atenção Primária à Saúde

Nessa categoria, propõe-se compilar os principais aspectos positivos da telenfermagem na APS, bem como os principais aspectos que necessitam de aprimoramento da prática, considerando-a uma estratégia complementar e não substitutiva da assistência. Ainda tendo em vista a normatização da telenfermagem, entre as seis definições e atribuições da prática mediadas por TICs, foram identificados nos estudos selecionados a consulta de enfermagem, a interconsulta, o monitoramento de enfermagem e a educação em saúde (Quadro 4).

**Quadro 4:** Principais aspectos positivos e aspectos que necessitam de aprimoramento para a prática da telenfermagem na Atenção Primária à Saúde, 2022

Aspectos positivos	Continuidade do cuidado^(11,15,20-22)^ Fortalece as orientações em saúde^(11,17,25,26)^ Melhora do vínculo entre profissionais e pacientes^(11,17)^ Fortalece o serviço multiprofissional e interprofissional^(16,26)^ Otimiza a assistência em saúde^(26)^ Possibilita a inclusão de novas tecnologias à atenção primária^(23)^ Melhora o controle de dados epidemiológicos^(24)^ Reduz o absenteísmo^(23,26)^ Aumenta os índices de vacinação^(17,19,26)^ Reduz os riscos de infecção e transmissão de doenças^(14,27)^ Reduz as barreiras físicas e geográficas^(14)^ Fornece acessibilidade aos serviços de saúde^(21,22)^ Fortalece as ações de promoção, prevenção e educação em saúde^(12,17,19,25)^ Facilita o diagnóstico, a prescrição de medicamentos e a avaliação de exames^(14,20)^ Reduz os custos com tempo e deslocamento^(14,17,18,22)^ Oferece conforto ao paciente^(17)^ Evita a sobrecarga hospitalar^(13,20)^ Estimula o autocuidado^(26)^ Melhora da oferta de cuidados à saúde mental^(16)^
Aspectos que necessitam de aprimoramento	Dificuldade de acesso e manuseio dos recursos tecnológicos^(12,14,17,18,20,22)^ Exclusão de pacientes com problemas com limitações físicas e cognitivas^(14,16,22)^ Necessidade de um cuidador ou familiar junto à teleconsulta^(14,16)^ Dificuldades de privacidade no domicílio^(17)^ Necessidade de investimento e diretrizes^(14,24)^ Impossibilita o exame físico^(17,22,23,26)^ Redução do rastreamento de doenças infectocontagiosas e de doenças crônicas^(21)^ Necessidade de capacitação, prática e experiência profissional^(21,22,25)^

Percebe-se que os estudos selecionados trouxeram dados relevantes sustentados pelas alternâncias entre os prós e contras provenientes das experiências tanto dos profissionais (enfermeiros/equipe multiprofissional) quanto dos pacientes. Além disso, os aspectos que necessitam de aprimoramento emergem de características e demandas específicas de determinados públicos-alvo.

### Perspectivas de consolidação da telenfermagem em um cenário pós-pandêmico

Em uma perspectiva pós-pandêmica, sugere-se que a telenfermagem, associada às consultas presenciais, seja implementada em um cenário pós-pandêmico da COVID-19^(12)^. Também sugere-se que alcance não somente grupos prioritários, mas a população em larga escala, como forma de avaliar a adesão de diferentes grupos populacionais, pois considera-se a telenfermagem uma estratégia de saúde promissora para os serviços de saúde^(21)^.

A partir das experiências adquiridas com a consulta de enfermagem mediadas pelas TICs em meio à pandemia de COVID-19, os profissionais da APS mostraram um olhar voltado para o futuro, almejando que a assistência possa ser intercalada entre consultas presenciais e virtuais^(22)^, levando em consideração o avanço tecnológico e a necessidade do uso da internet pela sociedade. Por esses motivos, tornam-se necessários investimentos financeiros por parte dos setores públicos e privados, além da promoção da telenfermagem como garantia de melhoria do acesso aos cuidados em saúde em situações de crise^(14,16)^.

Vislumbra-se a telenfermagem como uma oportunidade de melhoria futura para a assistência na APS, preservando, assim, a natureza cuidadosa e prática da enfermagem, uma vez que a prática incentivou o trabalho interprofissional e a comunicação entre as esferas da saúde. No entanto, as melhorias necessitam partir do investimento tecnológico, criação de diretrizes e capacitação profissional^(26)^.

Fica claro o interesse dos profissionais da APS em manter a telenfermagem para além da pandemia de COVID-19, proporcionando a aplicabilidade dessa modalidade assistencial em um cenário sem condições emergentes ou de crise. Os mesmos profissionais almejam que as consultas mediadas por TICs sejam realizadas em equilíbrio com as consultas presenciais, fazendo-se necessário realizar mais estudos relacionados ao assunto^(23)^.

Ademais, em tempos de crise, a tecnologia e os meios digitais têm grande potencial para manter a empregabilidade dos profissionais, dando continuidade à assistência à saúde da população^(15)^. Para isso, é necessário apoio e investimento por parte do governo, das empresas, das organizações e dos pesquisadores^(17)^.

## DISCUSSÃO

Os resultados desta revisão permitiram responder à questão norteadora proposta, fortalecendo as contribuições da telenfermagem na APS em meio à pandemia de COVID-19, além de proporcionar perspectivas de aprimoramento da prática e de sua consolidação em um cenário pós-pandêmico. Desta forma, utilizar a telenfermagem no âmbito da APS mostrou-se fundamental para a continuidade do cuidado em meio à pandemia tanto em situações agudas quanto em situações crônicas de saúde, além de demandas recorrentes da rotina assistencial da população^(27)^.

Somado a isso, o cuidado de forma remota proporcionou a ambos os envolvidos, profissional-paciente, o rompimento de barreiras geográficas e temporais, solucionando a distância entre a população e os serviços de saúde^(28)^. Ocorre que, por meio da telenfermagem, tanto a enfermagem quanto a equipe multiprofissional da APS conseguiram garantir o acesso aos serviços de saúde e a continuidade do cuidado a toda a população, entre crianças, adolescentes, adultos e idosos, independentemente do distanciamento físico e social, atendendo às mais variadas demandas de saúde. De fato, a telenfermagem se mostrou uma importante ferramenta tecnológica para manter a comunicação entre profissionais e pacientes.

A enfermagem, juntamente com a equipe interprofissional da APS, destacou-se em meio à pandemia de COVID-19 como protagonista no combate à disseminação da doença, garantindo a continuidade da assistência à população em meio às restrições de distanciamento e isolamento social. Por meio da implementação da teleconsulta em caráter emergencial, a garantia do cuidado foi mantida entre diagnósticos e ações de prevenção e recuperação da saúde. Com isso, a enfermagem se manteve na linha de frente à pandemia, momento esse em que os profissionais se viram diante de uma realidade de constante resiliência, aprimoramento e adaptação^(29)^.

Diante disso, a telenfermagem, por meio da consulta, interconsulta, monitoramento e educação em saúde mediadas por TICs, seja por meio de interação síncrona (consultas de voz e/ou vídeo) ou assíncrona (mensagens de voz e/ou textual), surge como uma inovação e transformação dos padrões assistenciais de saúde na APS no que diz respeito à produção do cuidado proporcionado pela enfermagem ao usuário. Com isso, a telenfermagem impulsionou mudanças nos fluxos de trabalho e gerenciamento em saúde. Além disso, influenciou as condutas de cada profissional e a perspectiva do paciente acerca de sua saúde, pois permitiu tanto ao profissional quanto ao paciente ampla autonomia para o cuidado.

Ainda, a telenfermagem vem contribuindo para a descoberta de fragilidades e potencialidades acerca da assistência em saúde, proporcionando parceria entre os profissionais e pacientes em prol do melhor caminho a ser traçado para a melhoria do cuidado. A prática tornou uma forte aliada para a comunicação entre a APS e a população, estreitando vínculos e fortalecendo a confiança entre os envolvidos^(30)^.

No entanto, diante da rápida expansão da telenfermagem, é percebido que se trata de uma prática que demanda aprimoramento, devido às circunstâncias do cenário pandêmico da COVID-19^(31)^. Foi preciso atentar para as questões de equidade, considerando a população que se encontra em situação de vulnerabilidade e que não possui acesso aos recursos tecnológicos adequados para a prática da telenfermagem^(27)^.

De fato, os estudos desta revisão mostraram que o acesso às tecnologias foi um fator que dificultou o alcance de certa parte da população, pois a realidade é que nem todos possuem subsídios para utilizar os recursos tecnológicos de forma adequada. Essas dificuldades se mostram presentes desde a capacidade cognitiva e/ou física do paciente e situação socioeconômica até a sua disponibilidade de recursos tecnológicos adequados para a aplicabilidade da telenfermagem. Somado a isso, mostrou-se presente, tanto para os profissionais quanto para os pacientes, a necessidade de consultas presenciais, devido a inseguranças relacionadas ao exame físico.

Para tanto, a telenfermagem está sendo uma oportunidade de melhoria dos serviços de saúde e uma evolução para as relações entre profissional e paciente, embora necessite de investimentos para o futuro para que os propósitos sejam alcançados e a qualidade da assistência seja mantida^(31)^.

### Limitações do estudo

Este estudo apresenta limitação referente ao recorte temporal, pois foram incluídos na revisão apenas estudos publicados entre janeiro de 2020 e agosto de 2022, considerando o recente período pandêmico da COVID-19. Além disso, os artigos selecionados para esta revisão limitam-se à realidade somente de alguns países e continentes, uma vez que existem culturas e sistemas de saúde distintos.

Reconhecemos que outros estudos, com outras perspectivas, serão de grande importância para entender o fenômeno da saúde digital e da telenfermagem no âmbito da APS, garantindo, assim, melhorias para a assistência à saúde.

### Contribuições para a área da enfermagem, saúde ou política pública

Considera-se que o presente estudo contribuiu para identificar as potencialidades e fragilidades da telenfermagem no âmbito da APS. Além disso, este estudo buscou proporcionar respaldo científico tanto para a prática assistencial quanto para novas pesquisas sobre o assunto, uma vez que a enfermagem, juntamente com a equipe interprofissional, necessita se fortalecer diante das necessidades atuais da sociedade contemporânea e da evolução tecnológica.

## CONSIDERAÇÕES FINAIS

A presente revisão integrativa possibilitou identificar as contribuições da telenfermagem na APS no contexto pandêmico da COVID-19, sendo possível compilar os principais aspectos positivos e aspectos que necessitam de aprimoramento para a prática, além de proporcionar a reflexão acerca da sua consolidação em um cenário pós-pandêmico.

Mesmo diante de uma crise epidemiológica, a APS passou por modificações e adaptações emergenciais, garantindo, por meio da telenfermagem, o acesso da população aos serviços de saúde, mantendo ações de educação, promoção e recuperação da saúde, além de ações de prevenção de doenças. Ainda, foi mantida a continuidade do cuidado, mesmo diante do distanciamento físico e social impostos pela pandemia. A telenfermagem foi e está sendo um recurso tecnológico essencial para ampliar o acesso aos serviços de saúde primários, apresentando mais vantagens do que desvantagens, tanto para os profissionais quanto para os pacientes.

Ademais, observaram-se como principais pontos positivos da telenfermagem a continuidade do cuidado, o fortalecimento das orientações em saúde, o aumento dos índices de vacinação, o fortalecimento das ações de promoção, prevenção e educação em saúde, e a redução de custos com tempo e deslocamento. Já os principais aspectos que necessitam de aprimoramento para a prática foram identificados como a dificuldade de acesso e manuseio dos recursos tecnológicos e impossibilidade em realizar o exame físico.

Considera-se que as tecnologias digitais em saúde se mostraram parte integrante da atual sociedade contemporânea, sendo necessário um olhar cuidadoso e crítico para os avanços tecnológicos.

## Data Availability

https://doi.org/10.48331/scielodata.GVWFPA
